# Development of a Mild and Versatile Directed Cycloaddition Approach to Pyridines

**DOI:** 10.1002/chem.201403916

**Published:** 2014-08-21

**Authors:** Sylvestre P J T Bachollet, Jérôme F Vivat, Dean C Cocker, Harry Adams, Joseph P A Harrity

**Affiliations:** [a] Department of Chemistry, University of SheffieldBrook Hill, Sheffield, S3 7HF (UK)

**Keywords:** boranes, cycloadditions, pyridines, regioselectivity, triazines

## Abstract

The aza-Diels–Alder cycloaddition of 1,2,4-triazines with alkynes offers a rapid and convenient method for the synthesis of highly substituted pyridines, but often requires harsh conditions and long reaction times. The present study offers a solution to these limitations by use of a temporary tether established by a Lewis acid–base complexation of in situ generated alkynylboranes and triazines bearing a Lewis basic donor. The cycloaddition reactions take place within 20 min at 40 °C and provide direct access to a broad range of pyridines with complete and predictable regiocontrol. The carbon—boron bond can be further functionalised by cross-coupling allowing further functionality to be introduced after cycloaddition.

## Introduction

Pyridines are a fundamentally important class of aromatic molecules.[1] They are present in many bioactive compounds and they play a key role in a number of biological processes. From a synthetic viewpoint, the ready quaternisation of the basic pyridine ring limits the functionalisation of this aromatic system by electrophilic substitution processes. Ring substitution is, therefore, often dictated by the availability of a halide substituent, or related group that allows elaboration by substitution or transition-metal-catalysed coupling. An alternative approach to pyridines is by means of ring synthesis and a number of approaches are now established.[2] In this regard, the inverse electron demand aza-Diels–Alder reaction of triazines constitutes a useful and much studied method, however this process has largely focused on the use of enamine dienophiles as alkyne surrogates because alkynes themselves only participate in [4+2] cycloadditions with triazines under very harsh conditions. Moreover, such processes are often poorly regioselective and are relatively low yielding.[3]

**Scheme 1:**
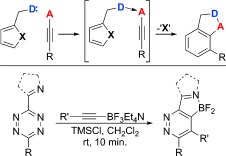
Directed cycloaddition reactions.

With regard to inverse electron demand aza-Diels–Alder reactions, we have recently become interested in the use of directed cycloadditions for the mild and regiocontrolled synthesis of aromatic and heteroaromatic compounds.[4] Central to our design was the use of an alkyne bearing a Lewis acid acceptor that would promote pre-association with a diene bearing a complementary Lewis base (Scheme 1). The resulting complex would provide a platform for rate enhancements in the ensuing cycloaddition, and this rate enhancement was exemplified by the reaction of tetrazines with in situ generated alkynyldifluoroboranes at ambient temperatures.

In considering an appropriate alkyne-substituted Lewis acid, boron-based acceptors are of particular interest as they deliver organoboron products of potential value for further organic synthesis.[5] We report herein the employment of this concept in a mild and versatile route to pyridine boronic acid derivatives by means of directed triazine cycloadditions.

## Results and Discussion

**Scheme 2:**
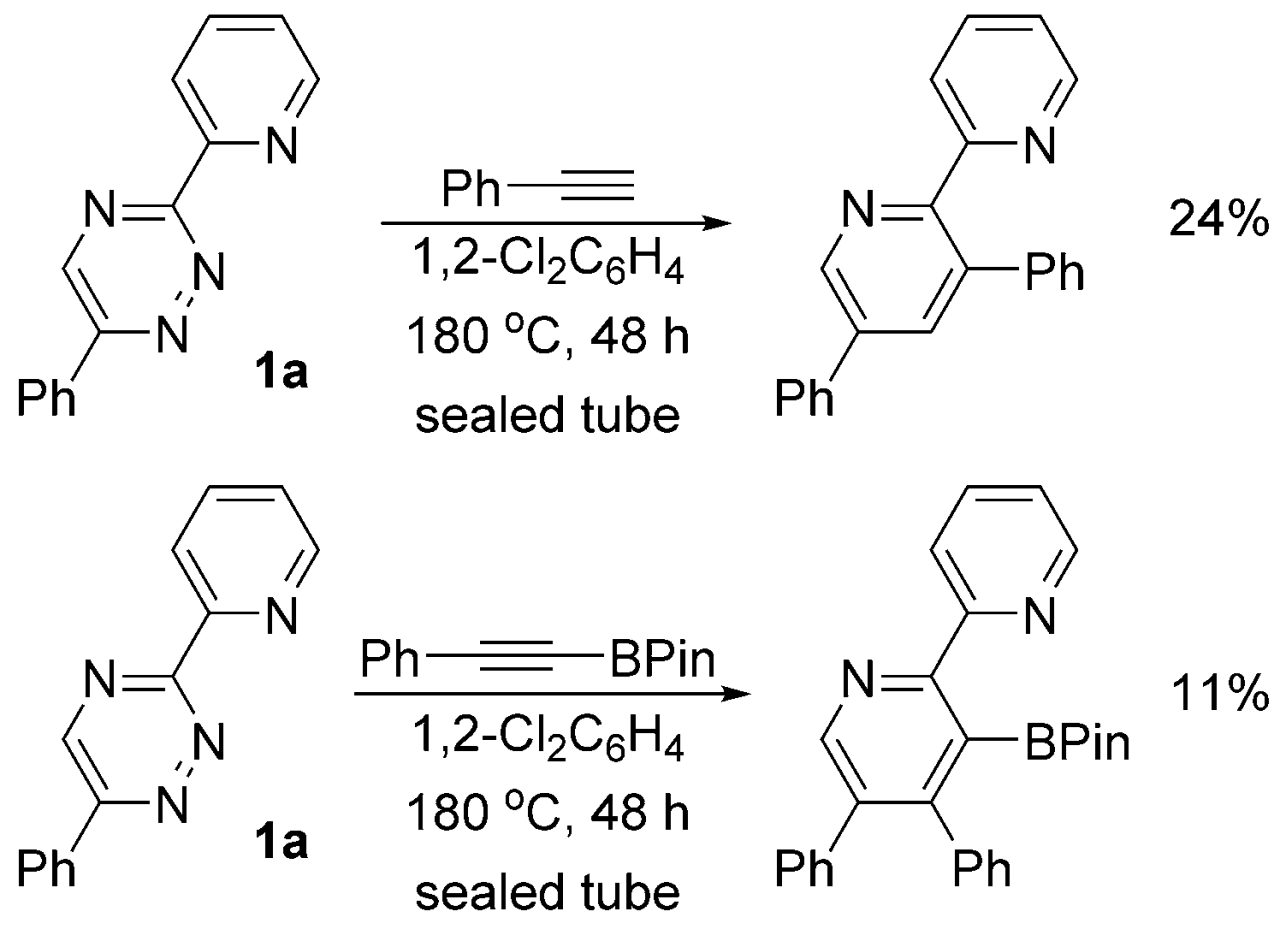
Triazine cycloaddition reactions of alkynes. Pin=pinacol.

To establish a typical reactivity profile for non-activated triazines and alkynes, we opted to explore the cycloaddition reactions of readily available alkynes and triazine. Indeed, we found that triazine **1 a** was particularly reluctant to undergo efficient reaction with phenylacetylene, providing the corresponding product in low yield after prolonged heating, albeit with high regiocontrol.[6] Moreover, we attempted a similar reaction with an alkynylboronate and found that this approach generated the corresponding pyridine boronic acid derivative, again in very low yield, but with high regioselectivity (Scheme 2).[7]

**Figure 1:**
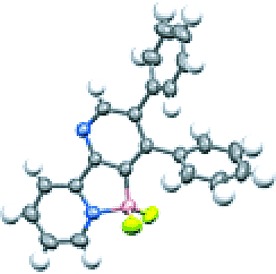
X-ray crystal structure representation of 3, H atoms omitted for clarity.

The poor reactivity of diene **1 a** with alkynes made it an ideal choice for evaluating the potential of our proposed directed cycloaddition, and we set out to explore the reaction of this compound with alkynytrifluoroborate **2 a**, our results are depicted in Table 1. Fluorophilic Lewis acids are known to transform alkynyltrifluoroborate salts into the corresponding difluoroboranes,[8], [9] and so we employed BF_3_**⋅**OEt_2_ to promote formation of our BF_2_-appended alkyne in situ. Remarkably, simply stirring this Lewis acid and substrate combination in CH_2_Cl_2_ at room temperature provided the desired cycloadduct (entry 1). The yield could be improved by increasing the temperature and the concentration of alkynydifluoroborane (entries 2 and 3). Finally, TMSCl was also found to be a competent fluorophile, albeit slightly less effective than BF_3_**⋅**OEt_2_ in this case (entry 4). Confirmation of the Lewis acid–base interaction between the pyridyl and BF_2_ substituents in the product, as well as the regioselectivity, was confirmed by X-ray crystallography. Figure 1 shows the expected tetrahedral geometry around the B atom.

**Table 1:** Directed cycloaddition of 1 and alkynyltrifluoroborates.[a]

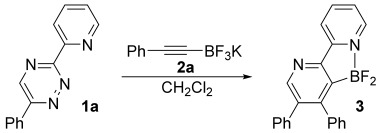				
Entry	Lewis acid^[a]^ [(equiv)]	*T* [°C]	*t* [min]	Yield3[%]
1	BF_3_**⋅**OEt_2_ (2)	25	16 h	29
2	BF_3_**⋅**OEt_2_ (2)	40	10	43
3	BF_3_**⋅**OEt_2_ (3)	40	10	84
4	Me_3_SiCl (3)	40	10	73

[a] A 1:1 stoichiometry of Lewis acid and alkyne was used in all cases.

**Figure 2:**
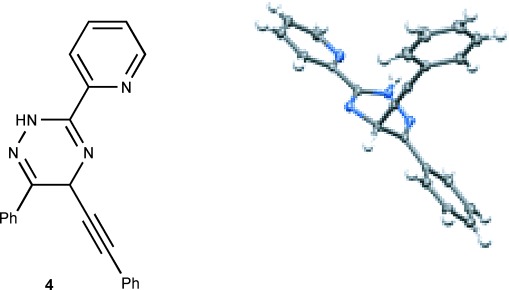
X-ray crystal structure representation of 4.

A minor side product observed in the cycloadditions of **1 a** and **2 a** was the product of direct acetylide addition at the heteroaromatic ring. This compound was isolated in 12 % yield under the optimal conditions (Table 1, entry 3), and its structure was also verified by X-ray crystallography (Figure 2).[10]

**Figure 3:**
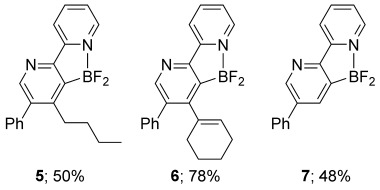
Pyridine products from the directed cycloaddition of 1 a. Conditions: 1 a (1 equiv), alkyne (3 equiv) and BF_3_⋅OEt_2_ (3 equiv) heated at 40 °C in CH_2_Cl_2_ for 10 min.

Notwithstanding the propensity for competing direct addition processes, the optimal conditions of the cycloaddition were found to be quite general across a small selection of alkynes, allowing the corresponding pyridines **5**–**7** to be generated in moderate to high yield (Figure 3).

Having established reaction conditions for the mild cycloaddition of triazine **1 a** with alkynyltrifluoroborates, we set out to explore the scope of this chemistry for the preparation of bipyridyldifluoroboranes, our results are shown in Table 2. We began by employing an isomer of triazine **1 a** and were pleased to find that pyridines **8** and **9** were formed in high yield (entries 1 and 2). Expanding to more heavily substituted triazines provided the opportunity to access fully substituted pyridines under mild conditions (entries 3–6). This approach is completely regioselective because of the nature of the directed reaction; therefore, this approach represents a powerful method for assembling highly functionalised products with entirely predictable regiocontrol. Finally, less heavily substituted pyridines can also be accessed by this strategy, compounds **14** and **15** were both prepared from triazine **1 e** in good yield.

**Table 2:** Directed cycloaddition of triazines and alkynyltrifluoroborates.

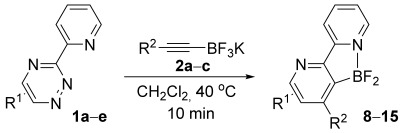					
Entry	Triazine	Alkyne (R^2^)	Product	Lewis acid^[a]^ [(equiv)]	Yield3 a[%]
1	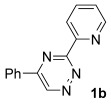	Ph; **2 a**	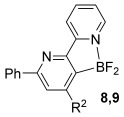	Me_3_SiCl (3)	R=Ph; 75 (**8**)
2		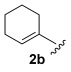		Me_3_SiCl (3)	R=C_6_H_9_; 83 (**9**)
3	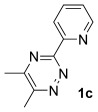	Ph; **2 a**	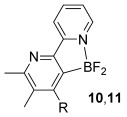	BF_3_**⋅**OEt_2_ (3)	R=Ph; 72 (**10**)
4		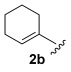		BF_3_**⋅**OEt_2_ (3)	R=C_6_H_9_; 76 (**11**)
5	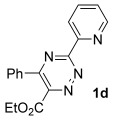	Ph; **2 a**	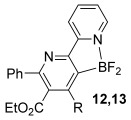	BF_3_**⋅**OEt_2_ (3)	R=Ph; 82 (**12**)
6		Bu; **2 c**		BF_3_**⋅**OEt_2_ (3)	R=Bu; 62 (**13**)
7	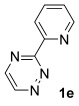	Ph; **2 a**	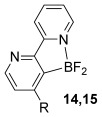	BF_3_**⋅**OEt_2_ (3)	R=Ph; 50 (**14**)
8		Bu; **2 c**		Me_3_SiCl (3)	R=Bu; 62 (**15**)

**Scheme 3:**
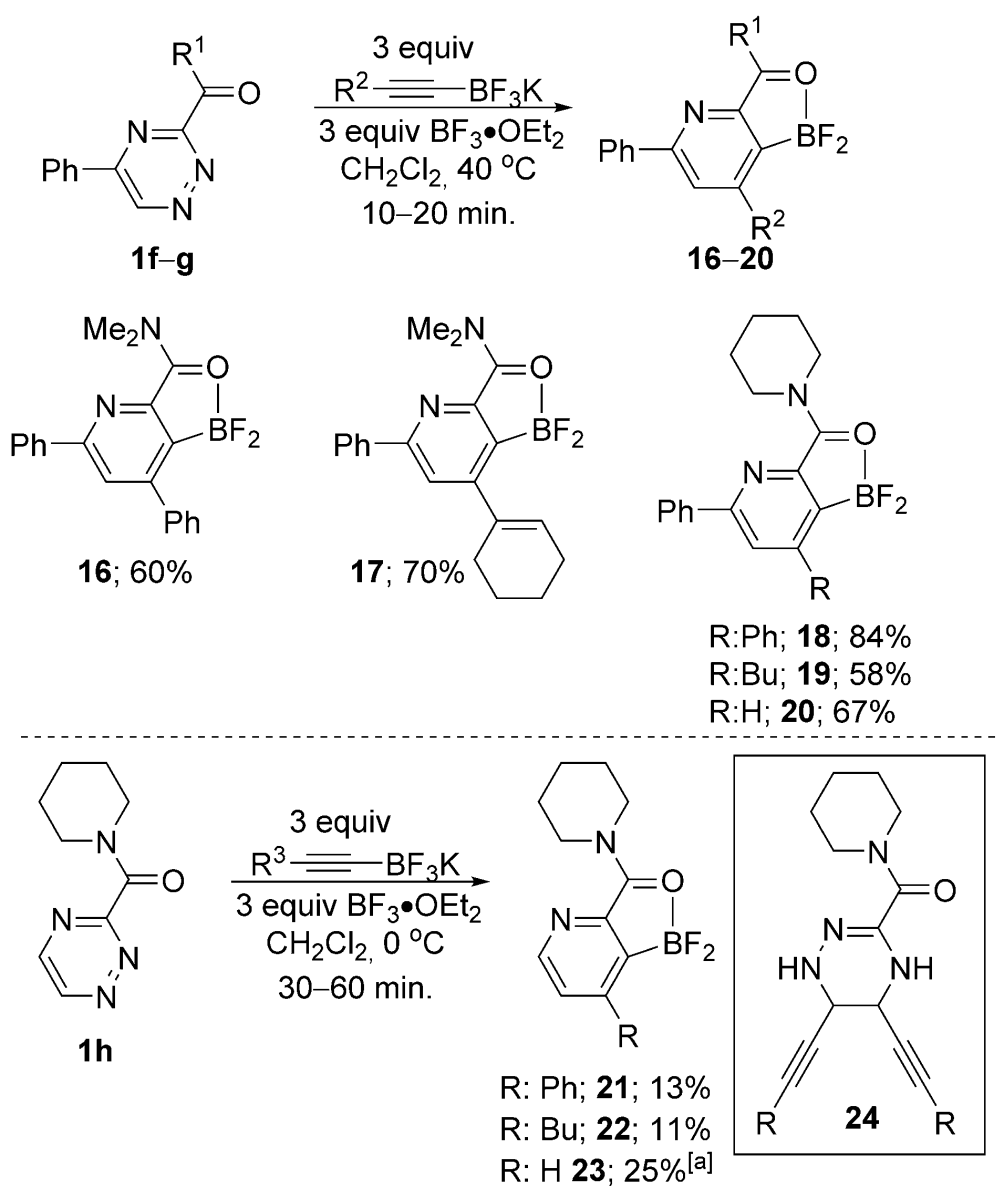
Alternative directing groups. [a] The reaction was conducted at 40 °C for 20 min.

Having had broad success with pyridyl directing groups, we decided to establish whether other Lewis bases could direct the cycloaddition reaction. Indeed, we were pleased to find that amides also functioned as competent directing groups, providing access to pyridines **16**–**20** in good overall yield (Scheme 3). Interestingly, the less substituted triazine substrate **1 h** was significantly less efficient, providing poor yields of the corresponding pyridines even when the reaction was conducted at low temperature. In this case, the crude mixtures were relatively complex, but the major side product in each case, **24**, appeared to result from alkyne addition to the ring.[11]

**Scheme 4:**
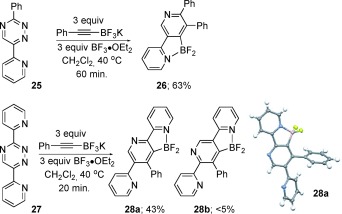
Incorporation of a directing group at C6.

A further issue that we wished to clarify was the importance of the positioning of the directing group. In principle, the Lewis basic donor could also be incorporated at the 6-position of the triazine giving rise to isomeric pyridine products. As shown in Scheme 4, the cycloaddition of **25** was found to proceed in good yield, although the reaction required a longer time period and returned a small amount of starting triazine **25** (≈10 %). We also prepared **27** to probe the effect of having two competing directing groups on reaction regiochemistry. Interestingly, the reaction proceeded with high selectivity to provide **28 a**, albeit in modest yield,[12] and <5 % of regioisomer **28 b** (as judged by LC-MS analysis). This preliminary data suggest that substrates bearing a directing group at the 3-position are optimal, but that the inclusion of directing groups at C6 are viable. Further studies aimed at understanding the scope of directing-group positioning are currently being pursued.

**Scheme 5:**
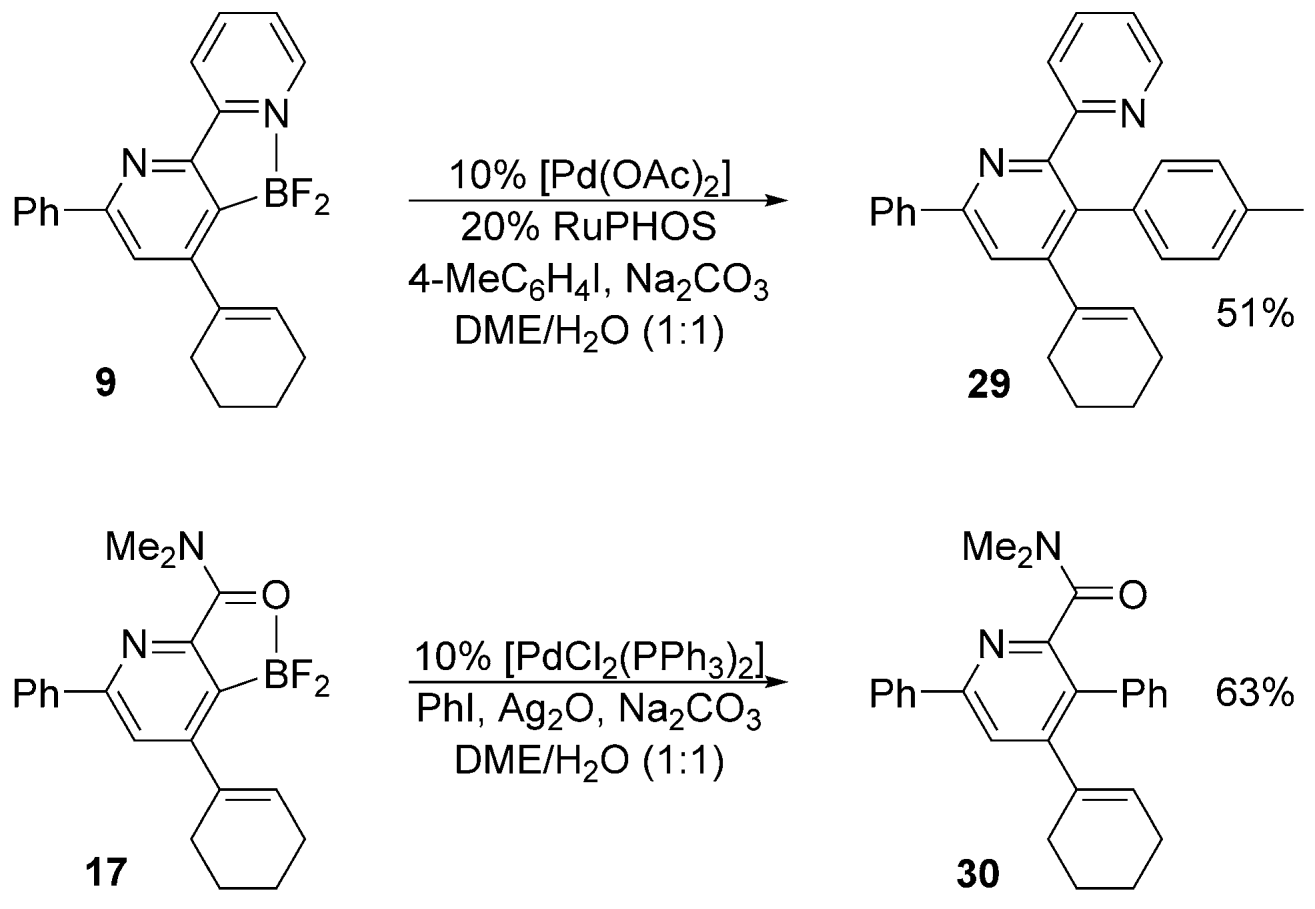
Reactions of cycloadducts. RuPHOS=2-dicyclohexylphosphino-2′,6′-diisopropoxybiphenyl, DME=1,2-dimethoxyethane.

Although the main objective of this study was to demonstrate that the directed cycloaddition could deliver faster reactions than the traditional aza-Diels–Alder process, we recognised the potential value of the products that are armed with a carbon—boron bond. We decided to explore the Pd-catalysed cross coupling of two representative difluoroboranes, **9** and **17**, which contain multiple functionality and a hindered borane unit. In the event, both reactions required some optimisation, but delivered the corresponding biaryl products in acceptable yields (Scheme 5).

## Conclusion

We have developed a mild and regiocontrolled method for the synthesis of highly substituted pyridines by means of a Lewis base directed cycloaddition of triazines and in situ generated alkynylboranes. This method proceeds with a range of alkynes and triazines, although it appears to be advantageous to have the Lewis base directing group at C3 of the diene cycloaddition partner. As well as providing a convenient means for generating bipyridines, this method is compatible with amide directing groups and the presence of the carbon—boron bond allows further functionalisation to take place through cross-coupling reactions.

## Experimental Section

### General procedure for the cycloaddition of alkynyltrifluoroborates and triazines

**Synthesis of 3**: A solution of 6-phenyl-3-(2-pyridyl)-1,2,4-triazine **1 a** (50 mg, 0.21 mmol) and potassium (phenylethynyl)trifluoroborate **2 a** (132 mg, 0.64 mmol) in CH_2_Cl_2_ (2 mL) was treated with BF_3_**⋅**OEt_2_ (55 μL, 0.64 mmol). The reaction was stirred for 10 min and then quenched with brine (10 mL). The mixture was extracted with CH_2_Cl_2_ (3×15 mL) and the extract dried over MgSO_4_, filtered and the solvent evaporated. The residue was purified chromatographically over silica gel (gradient; starting with petroleum ether, ending with ethyl acetate) to afford 3-(difluoroboryl)-4,5-diphenyl-2,2′-bipyridine **3** (63 mg, 84 %) as a colourless solid. M.p 225–226 °C. ^1^H NMR (400 MHz, CDCl_3_): *δ*=7.18–7.22 (2 H, m), 7.25–7.33 (6 H, m), 7.35–7.40 (2 H, m), 7.61–7.67 (1 H, m), 8.26 (1 H, td, *J*=7.5, 1.5 Hz), 8.40 (1 H, d, *J*=8.0), 8.59 (1 H, d, *J*=5.5 Hz), 8.67 ppm (1 H, s); ^13^C NMR (100.6 MHz, CDCl_3_): *δ*=118.9, 125.0, 127.4, 127.7, 127.8, 127.9, 128.2, 129.8, 129.9, 138.2, 138.5, 141.4, 144.1, 151.7, 151.9, 154.6, 154.9 ppm; ^19^F NMR (235.1 MHz, CDCl_3_): *δ*=−156.4 ppm; FTIR: 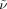
=3058 (w), 2925 (w), 1626 (s), 1578 (m), 1555 (m), 1489 (s), 1452 (m), 1433 (s), 1158 (m), 1131 (s), 1100 (s), 1007 (m), 910 (m) cm^−1^. HRMS: (ESI) *m*/*z* calcd for C_22_H_15_^11^BF_2_N_2_Na: 379.1194 [*M*+Na^+^], found 379.1204.
