# Supplementary material for: Development of a Mild and Versatile Directed Cycloaddition Approach to Pyridines
Source: Chemistry. 2014 Aug 21;20(40):12889–93. doi: 10.1002/chem.201403916 (PMC4313684; doi:10.1002/chem.201403916)
Supplement: Supplementary file 1 [file chem0020-12889-sd1.pdf]

CHEMISTRY
A European Journal

Supporting Information

© Copyright Wiley-VCH Verlag GmbH & Co. KGaA, 69451 Weinheim, 2014

Development of a Mild and Versatile Directed Cycloaddition Approach to Pyridines

Sylvestre P. J. T. Bachollet, Jérôme F. Vivat†, Dean C. Cocker, Harry Adams, and Joseph P. A. Harrity*[a]

chem_201403916_sm_miscellaneous_information.pdf

Contents

General Procedures S2

Cycloaddition on 3-(2-pyridyl)-1,2,4-triazines S3

Synthesis of alternative 3-substituted-1,2,4-triazines S11

Cycloaddition on alternative 3-substituted-1,2,4-triazines S13

Application of difluoroboryl-pyridines S19

^{1}H NMR, ^{13}C NMR and NOE Spectra of Novel Compounds S21

General Procedures

All reactions were conducted in oven or flame-dried glassware under an inert atmosphere of dry nitrogen. Flash chromatography was performed on silica gel (Fluorochem Davisil silica gel 43-60). The solvent system used was a gradient of petroleum ether (40-60), increasing in polarity to ethyl acetate, unless otherwise stated. Thin layer chromatography (TLC) was performed on aluminium backed plates pre-coated with silica (0.2 mm, Merck DC-alufolien Kieselgel 60 F_{254}) which were developed using standard visualizing agents: Ultraviolet light or potassium permanganate.

^{1}H NMR spectra were recorded on a Bruker AV-250 (250 MHz), AV-400 (400 MHz) or DRX-500 (500 MHz) supported by an Aspect 3000 data system. Chemical shifts are reported in ppm with the solvent resonance as the internal standard ($CHCl_3$:δ 7.26 ppm). Data are reported as follows: chemical shift, integration, multiplicity (s = singlet, d = doublet, t = triplet, q = quartet, sept = septet, br = broad, m = multiplet), coupling constants (*J*) in Hz, and assignment.

^{13}C NMR spectra were recorded on a Bruker AC-250 (62.9 MHz), AMX-400 (100.5 MHz) or DRX-500 (125.8 MHz) with complete proton decoupling. Chemical shifts are reported in ppm with the solvent resonance as the internal standard ($CDCl_3$: δ 77.0 ppm). It has to be highlighted that in the spectra of boron containing compounds the carbon atom directly *alpha* to boron is often too broad to be detected.

^{19}F NMR spectra were recorded on a Bruker AC-250 (235.1 MHz), Chemical shifts are reported in ppm with $CFCF_3$ as an external standard ($CFCF_3$: δ 0.0 ppm).

Infrared (FTIR) spectra were recorded on a Perkin Elmer Paragon 100 FTIR spectrophotometer, ν_{max} in cm^{-1}. Bands are characterized as broad (br), strong (s), medium (m) and weak (w). Samples were recorded as thin films using sodium chloride plates.

Low resolution mass spectra were recorded on Micromass Autospec, operating in E.I., C.I. or FAB mode; or a Perkin-Elmer Turbomass Benchtop GC-MS operating in either E.I. or C.I mode. High-resolution mass spectroscopy (HRMS) recorded for accurate mass analysis, were performed on either a MicroMass LCT operating in Electrospray mode (TOF ES^+) or a MicroMass Prospec operating in either FAB (FAB^+), EI (EI^+) or CI (CI^+) mode.

Melting points were performed on recrystallised solids and recorded on a Gallenkamp melting point apparatus and are uncorrected. All solvents and reagents were purified using standard laboratory techniques according to methods published in “Purification of Laboratory Chemicals”

by Perrin, Armarego, and Perrin (Pergamon Press, 1966). Potassium alkynyltrifluoroborates[1] and triazines[2-9] were prepared according to literature procedures or were purchased from commercial sources. The synthesis and characterization of pyridine **15** has been reported elsewhere.[10]

Cycloaddition of 3-(2-pyridyl)-1,2,4-triazines

Synthesis of 3,5-diphenyl-2,2'-bipyridine.

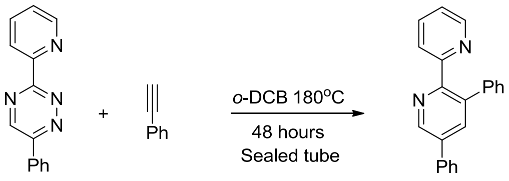

6-Phenyl-3-(2-pyridyl)-1,2,4-triazine (50 mg, 0.213 mmol) and phenylacetylene (70 μL, 0.640 mmol) were heated at 180 °C in a sealed vessel for 48 hours. Chromatographic purification over silica gel (gradient; starting with petroleum ether, ending with ethyl acetate) afforded 3,5-diphenyl-2,2'-bipyridine (16 mg, 24%) as a colourless solid. M.p 90-91°C. ^{1}H NMR (400 MHz, $CDCl_3$): δ 7.20-7.39 (11H, m, Ar), 7.88 (1H, td, *J* = 2.0, 8.0 Hz, Ar), 8.47-8.52 (2H, m, Ar), 8.71-8.76 (2H, m, Ar). ^{13}C NMR (100.6 MHz, $CDCl_3$): δ 121.2, 122.2, 123.7, 127.4, 127.8, 128.2, 128.3, 129.5, 129.8, 135.8, 137.0, 137.7, 138.9, 148.8, 149.3, 150.5, 155.1, 156.0. FTIR: 2925 (w), 1583 (s), 1457 (s), 1371 (m) cm^{-1}. HRMS: (ESI) [MH$^+$] calcd for $C_{22}H_{17}N_2$: 309.1392, found 309.1379.

Synthesis of 4,5-diphenyl-3-(4,4,5,5-tetramethyl-1,3,2-dioxaborolan-2-yl)-2,2'-bipyridine.

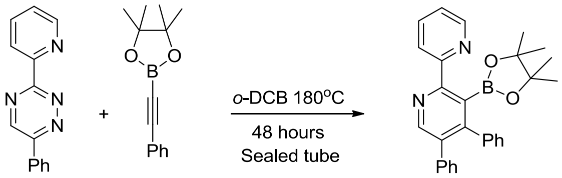

---

[1] Molander, G.; Katona, B.W.; Machrouhi, F. *J. Org. Chem.* **2002**, *67*, 8416. b) Yamamoto, Y.; Hattori, K.; Ishii, J.; Nishiyama, H. *Tetrahedron*. **2006**, *62*, 4294.
[2] Sauer, J.; Heldmann, D.K.; Pabst, G.R.; *Eur. J. Org. Chem*. **1999**, *1*, 313-321.
[3] Kozhevnikov, V.N.; Kozhevnikov, D.N.; Shabunina, O.V.; Rusinov, V.L.; Chupakhin, O.L. *Tet. Lett.* **2005**, *46*, 1791–1793.
[4] Altuna-Urquijo, M.; Gehre, A.; Stanforth, S.P.; Tarbit, B. *Tetrahedron*. **2009**, *65*, 975–984.
[5] Ye, L.; Haddadin, M.J.; Lodewyk, M.W.; Ferreira, A.J.; Fettinger, J.C.; Tantillo, D.J.; Kurth. M.J. *Org. Lett.*, **2010**, *12*, 164–167
[6] Benson, S.C.; Gross, J.L.; Snyder, J.K. *J. Org. Chem.* **1990**, *55*, 3257-3269.
[7] O'Rourke, M.; Lang, S. A.; Cohen, E. *J. Med. Chem.* **1977**, *20*, 723-726.
[8] Maheshwari, V.; Bhattacharyva, D.; Fronczek, F. R. Marzilli, P. A. Marzilli, L. G. *Inorg Chem*. **2006**, *45*, 7182-7190.
[9] Paudler, W. W.; Barton, J. M. *J. Org. Chem.* **1966**, *31*, 1720.
[10] Vivat, J. F.; Adams, H.; Harrity, J. P. A. *Org. Lett.* **2010**, *12*, 160.

6-Phenyl-3-(2-pyridyl)-1,2,4-triazine (50 mg, 0.213 mmol) and 4,4,5,5-tetramethyl-2-(phenylethynyl)-1,3,2-dioxaborolane (145 mg, 0.640 mmol) were heated at 180 ^{o}C in a sealed vessel for 48 hours. Chromatographic purification over silica gel (gradient; starting with petroleum ether, ending with ethyl acetate) afforded 4,5-diphenyl-3-(4,4,5,5-tetramethyl-1,3,2-dioxaborolan-2-yl)-2,2'-bipyridine (10 mg, 11%) as a colourless solid. M.p 189-190 °C. ^{1}H NMR (400 MHz, $CDCl_3$): δ 1.04 (12H, s, CH_3), 7.02-7.09 (2H, m, Ar), 7.15-7.21 (3H, m, Ar), 7.24 (5H, s, Ar), 7.39-7.46 (1H, m, Ar), 7.98 (1H, td, *J* = 1.5, 7.5 Hz, Ar), 8.46 (1H, d, *J* = 8.0 Hz, Ar), 8.59 (1H, s, Ar) 8.68 (1H, d, *J* = 5.0 Hz, Ar). ^{13}C NMR (100.6 MHz, $CDCl_3$): δ 26.8, 82.3, 119.9, 124.1, 126.8, 127.2, 127.4, 127.7, 129.9, 130.5, 137.7, 138.2, 138.9, 139.5, 144.4, 150.2, 153.2, 155.5, 156.2. FTIR: 2972 (m), 1626 (s), 1577 (w), 1536 (m), 1425 (s), 1342 (m), 1301 (m), 1138 (s), 1045 (m) cm^{-1}. HRMS: (ESI) [MH^+] calcd for $C_{28}H_{28}{}^{11}BF_2N_2O_2$: 435.2244, found 435.2233.

General procedure for $BF_3.OEt_2$-promoted cycloadditions of triazines with alkynyltrifluoroborate salts.

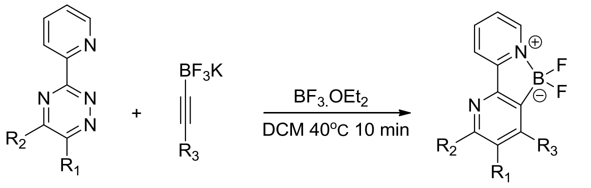

To a solution of triazine and alkynyltrifluoroborate salt (3 eq) in refluxing DCM was added dropwise $BF_3.OEt_2$ (3 eq). The reaction was stirred for 10 minutes. Brine was added (10 mL) and the mixture was extracted with DCM (3 x 15 mL). The organic extract was dried over $MgSO_4$, filtered and the solvent evaporated. The residue was purified chromatographically over silica gel (gradient; starting with petroleum ether, ending with ethyl acetate).

Synthesis of 3-(difluoroboryl)-4,5-diphenyl-2,2'-bipyridine (3).

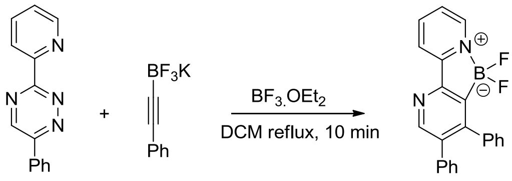

Following the general procedure, a solution of 6-phenyl-3-(2-pyridyl)-1,2,4-triazine (50 mg, 0.21 mmol) and potassium (phenylethynyl)trifluoroborate (132 mg, 0.64 mmol) in DCM (2 mL) was

treated with $BF_3.OEt_2$ (55 µL, 0.64 mmol). Chromatographic purification over silica gel (gradient; starting with petroleum ether, ending with ethyl acetate) afforded 3-(difluoroboryl)-4,5-diphenyl-2,2'-bipyridine (63 mg, 84%) as a colourless solid. M.p 225-226 °C. ^{1}H NMR (400 MHz, $CDCl_3$): δ 7.18-7.22 (2H, m, Ar), 7.25-7.33 (6H, m, Ar), 7.35-7.40 (2H, m, Ar), 7.61-7.67 (1H, m, Ar), 8.26 (1H, dt, *J* = 1.5, 7.5 Hz, Ar), 8.40 (1H, d, *J* = 8.0, Ar), 8.59 (1H, d, *J* = 5.5 Hz, Ar), 8.67 (1H, s, Ar). ^{13}C NMR (100.6 MHz, $CDCl_3$): δ 118.9, 125.0, 127.4, 127.7, 127.8, 127.9, 128.2, 129.8, 129.9, 138.2, 138.5, 141.4, 144.1, 151.7, 151.9, 154.6, 154.9. ^{19}F NMR (235.1 MHz, $CDCl_3$): δ -156.4. FTIR: 3058 (w), 2925 (w), 1626 (s), 1578 (m), 1555 (m), 1489 (s), 1452 (m), 1433 (s), 1158 (m), 1131 (s), 1100 (s), 1007 (m), 910 (m) cm^{-1}. HRMS: (ESI) [MNa^+] calcd for $C_{22}H_{15}{}^{11}BF_2N_2Na$: 379.1194, found 379.1204.

Synthesis of 4-butyl-3-(difluoroboryl)-5-phenyl-2,2'-bipyridine (5).

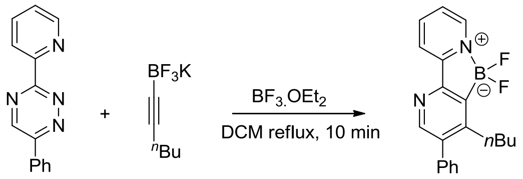

Following the general procedure, a solution of 6-phenyl-3-(2-pyridyl)-1,2,4-triazine (30 mg, 0.128 mmol) potassium (hex-1-ynyl)trifluoroborate (72 mg, 0.385 mmol) in DCM (1 mL) was treated with $BF_3.OEt_2$ (47 µL, 0.385 mmol). Chromatographic purification over silica gel (gradient; starting with petroleum ether, ending with ethyl acetate) afforded 4-butyl-3-(difluoroboryl)-5-phenyl-2,2'-bipyridine (54 mg, 50%) as a colourless oil. ^{1}H NMR (250 Hz, $CDCl_3$) δ 0.78 (3H, t, *J* = 7.0 Hz, CH_3), 1.20-1.35 (2H, m, CH_2), 1.45-1.65 (2H, m, CH_2), 2.70-2.90 (2H, m, CH_2), 7.30-7.50 (5H, m, Ar), 7.55-7.65 (1H, m, Ar), 8.22 (1H, td, *J* = 1.5, 8.0 Hz, Ar), 8.34 (1H, d, *J* = 8.0 Hz, Ar), 8.42 (1H, s, Ar), 8.62 (1H, d, *J* = 8.0 Hz, Ar). ^{13}C NMR (62.9 MHz, $CDCl_3$) δ 13.6, 22.8, 32.0, 32.8, 118.8, 124.7, 127.6, 128.3, 129.3, 138.7, 140.0, 141.3, 144.1, 151.3, 153.6, 154.2, 155.4. ^{19}F NMR (235 MHz, $CDCl_3$) δ -160.0. FTIR. Thin film. 3058 (w), 2958 (m), 2931 (m), 2871 (m), 1626 (s), 1602 (w), 1578 (s), 1552 (s), 1489 (s), 1452 (s), 1304 (w), 1204 (m), 1072 (s), 1009 (s), 747 (s), 703 (s) cm^{-1}. (ESI) [MH^+] calcd for $C_{20}H_{20}BN_2F_2$: 337.1688 found 337.1691.

Synthesis of potassium 4-(cyclohex-1-en-1-yl)-3-(difluoroboryl)-5-phenyl-2,2'-bipyridine (6).

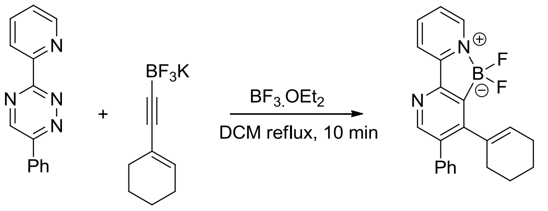

Following the general procedure, a solution of 6-phenyl-3-(2-pyridyl)-1,2,4-triazine (50 mg, 0.213 mmol) and potassium (cyclohex-1-en-1-ylethynyl)trifluoroborate (135 mg, 0.639 mmol) in DCM (2 mL) was treated with BF_3.OEt_2 (79 µL, 0.639 mmol). Chromatographic purification over silica gel (gradient; starting with petroleum ether, ending with ethyl acetate) afforded 4-(cyclohex-1-en-1-yl)-3-(difluoroboryl)-5-phenyl-2,2'-bipyridine (60 mg, 78%) as a colourless solid. M.p 159-160 °C. ^{1}H NMR (400 Hz, $CDCl_3$) δ 1.47-1.70 (4H, m, CH_2), 1.87-1.95 (2H, m, CH_2), 2.18-2.23 (2H, m, CH_2), 5.96 (1H, s, CH), 7.34-7.54 (5H, m, Ar), 7.61-7.67 (1H, m, Ar), 8.25 (1H, td, *J* = 1.5, 8.0 Hz), 8.34 (1H, d, *J* = 8.0 Hz), 8.53 (1H, s, Ar), 8.60 (1H, d, *J* = 6.0 Hz). ^{13}C NMR (100.6 MHz, $CDCl_3$) δ 21.8, 22.7, 25.5, 28.4, 118.8, 124.8, 127.6, 128.2, 129.1, 129.4, 136.1, 137.7, 138.7, 141.3, 143.9, 151.3, 154.4, 154.6, 155.1. ^{19}F NMR (235 MHz, $CDCl_3$) δ -158.0. FTIR. Thin film. 2928 (w), 1627 (m), 1488 (s), 1454 (m), 1435 (m), 1253 (m), 1100 (s), 1007 (s) cm^{-1}. (ESI) [MH^+] calcd for $C_{22}H_{20}BN_2F_2$: 361.1688 found 361.1696.

Synthesis of 3-(difluoroboryl)-5-phenyl-2,2'-bipyridine (7).

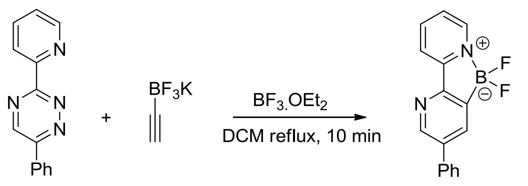

Following the general procedure, a solution of 6-phenyl-3-(2-pyridyl)-1,2,4-triazine (100 mg, 0.42 mmol) and potassium ethynyltrifluoroborate (169 mg, 1.2 mmol) in DCM (2 mL) was treated with BF_3.OEt_2 (55 µL, 1.2 mmol). Chromatographic purification over silica gel (gradient; starting with petroleum ether, ending with ethyl acetate) afforded 3-(difluoroboryl)-5-phenyl-2,2'-bipyridine (54 mg, 48%) as a colourless solid. M.p 245-248 °C. ^{1}H NMR (250 Hz, $CDCl_3$) δ 7.40-7.55 (3H, m, Ar), 7.60-7.70 (3H, m, Ar), 8.20-8.38 (3H, m, Ar), 8.62 (1H, d, *J* = 5.5 Hz), 8.75 (1H, d, *J* = 2.0 Hz). ^{13}C NMR (100.6 MHz, $CDCl_3$) δ 117.9, 123.9, 126.3, 127.4, 128.1, 135.7, 136.9, 137.9, 140.5, 143.1, 148.1, 153.4, 154.0. ^{19}F NMR (235 MHz, $CDCl_3$) δ -160.5.

FTIR. Thin film 3044 (w), 2923 (w), 1628 (m), 1555 (w), 1429 (s), 1486 (s), 1313 (m), 1007 (m), 1078 (s), 1007 (s), 762 (s) cm^{-1}. (ESI) [MH$^+$] calcd for $C_{16}H_{12}BN_2F_2$: 281.1062 found 282.1067.

Synthesis of 3-(difluoroboryl)-4,6-diphenyl-2,2'-bipyridine (8).

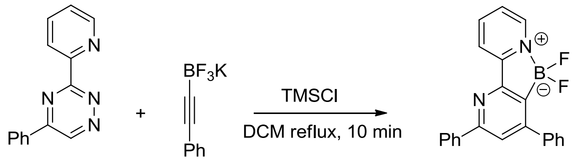

Following the general procedure, a solution of 5-phenyl-3-(2-pyridyl)-1,2,4-triazine (380 mg, 1.6 mmol) and potassium (phenylethynyl)trifluoroborate (1.01 g, 4.8 mmol) in DCM (25 mL) was treated with TMSCl (0.65 mL, 4.8 mmol). Chromatographic purification over silica gel (gradient; starting with petroleum ether, ending with ethyl acetate) afforded 3-(difluoroboryl)-4,6-diphenyl-2,2'-bipyridine (508 mg, 89%) as a colourless solid. M.p 187-189 ^{o}C. ^{1}H NMR (250 Hz, $CDCl_3$) δ 7.44-7.61 (6H, m, Ar), 7.62-7.70 (1H, m, Ar), 7.94-8.04 (3H, m, Ar), 8.14-8.21 (2H, m, Ar), 8.27 (1H, td, *J* = 1.5, 8.0 Hz), 8.52 (1H, d, *J* = 8.0 Hz, Ar), 8.63 (1H, d, *J* = 5.5 Hz). ^{13}C NMR (62.9 MHz, $CDCl_3$) δ 119.3, 122.1, 125.1, 127.1, 128.3, 128.9, 129.3, 131.7, 139.1, 139.7, 141.2, 144.0, 145.1, 153.8, 155.5, 156.5, 158.7. ^{19}F NMR (235 MHz, $CDCl_3$) δ -155.9. FTIR. Thin film. 3061 (w), 2925 (w), 1627 (s), 1586 (s), 1572 (s), 1538 (w), 1482 (s), 1427 (w), 1358 (w), 1358(m), 1256 (w), 1174 (m), 1100 (s), 1074 (s), 995 (m), 1004 (m), 748 (s) cm^{-1}. (ESI) [MH$^+$] calcd for $C_{22}H_{16}BN_2F_2$: 357.1363 found 357.1375.

Synthesis of 4-(cyclohex-1-en-1-yl)-3-(difluoroboryl)-6-phenyl-2,2'-bipyridine (9).

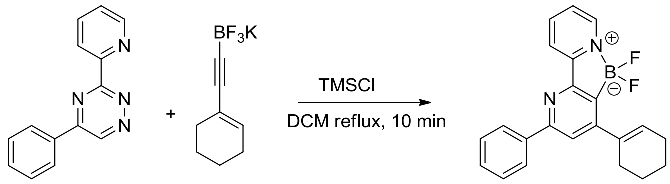

Following the general procedure, a solution of 5-phenyl-3-(pyridin-2-yl)-1,2,4-triazine (1.0 g, 4.27 mmol) and potassium (cyclohex-1-en-1-ylethynyl)trifluoroborate (2.72 g, 12.81 mmol) in DCM (50 mL) was treated with TMSCl (1.65 mL, 12.81 mmol). Chromatographic purification over silica gel (gradient; starting with petroleum ether, ending with ethyl acetate) afforded 4-(cyclohex-1-en-1-yl)-3-(difluoroboryl)-6-phenyl-2,2'-bipyridine (1.27 g, 83%) as a yellow solid. M.p 170-172 ^{o}C. ^{1}H NMR (400 Hz, $CDCl_3$) δ 1.72-1.80 (2H, m, CH_2), 1.84-1.92 (2H, m, CH_2),

2.31-2.38 (2H, m, CH_2), 2.59-2.67 (2H, m, CH_2), 6.61-6.67 (1H, m, CH), 7.43-7.56 (3H, m, Ar), 7.65 (1H, ddd, J = 1.0, 5.5, 8.0 Hz, Ar), 7.74 (1H, s, Ar-CH), 8.10-8.14 (2H, m, Ar), 8.25 (1H, td, J = 1.5, 7.5 Hz, Ar), 8.48 (1H, d, J = 7.5 Hz, Ar), 8.61 (1H, d, J = 5.5 Hz, Ar). ^{13}C NMR (100.6 MHz, $CDCl_3$) δ 21.9, 23.0, 26.1, 27.5, 119.2, 119.9, 124.8, 127.0 (x2), 128.7 (x2), 129.0, 130.2, 136.5, 139.4, 140.9, 143.8, 155.7, 158.3. ^{19}F NMR (235 MHz, $CDCl_3$) δ -157.0. FTIR, Thin Film. 2930 (m), 1627 (s), 1570 (s), 1489 (s), 1254 (m), 1136 (m), 1095 (s), 997 (s) cm^{-1}. (ESI) [MH^+] calcd for $C_{22}H_{20}{}^{10}BN_2F_2$: 360.1724 found 360.1711.

Synthesis of 3-(difluoroboryl)-5,6-dimethyl-4-phenyl-2,2'-bipyridine (10).

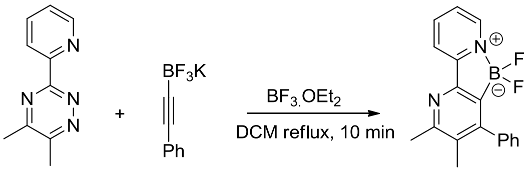

Following the general procedure, a solution of 5,6-dimethyl-3-(pyridin-2-yl)-1,2,4-triazine (40 mg, 0.21 mmol) and potassium (phenylethynyl)trifluoroborate (133 mg, 0.64 mmol) in DCM (2 mL) was treated with BF_3.OEt_2 (55 µL, 0.64 mmol). Chromatographic purification over silica gel (gradient; starting with petroleum ether, ending with ethyl acetate) afforded 3-(difluoroboryl)-5,6-dimethyl-4-phenyl-2,2'-bipyridine (47 mg, 72%) as a yellow oil. ^{1}H NMR (250 Hz, $CDCl_3$) δ 2.24 (3H, s, CH_3), 2.66 (3H, s, CH_3), 7.32-7.55 (6H, m, Ar), 8.17 (1H, td, J = 1.5, 8.0 Hz), 8.32 (1H, d, J = 8.0 Hz), 8.48 (1H, d, J = 5.5 Hz). ^{13}C NMR (62.9 MHz, $CDCl_3$) δ 16.8, 23.7, 118.5, 124.3, 127.6, 128.1, 128.9, 132.1, 139.3, 141.1, 143.7, 152.0, 152.9, 155.6, 159.0. ^{19}F NMR (235 MHz, $CDCl_3$) δ -158.2. FTIR, Thin Film. 3058 (w), 2922 (w), 2250 (w), 1626 (s), 1577 (s), 1558 (s) 1487 (s), 1443 (m), 1385 (m), 1346 (m), 1289 (w), 1256 (m), 1189 (m), 1136 (m), 1079 (s), 1030 (s), 987 (w), 947 (s), 911 (w), 819 (s) cm^{-1}. (ESI) [MH^+] calcd for $C_{18}H_{16}BN_2F_2$: 309.1375 found 309.1379.

Synthesis of 4-(cyclohex-1-en-1-yl)-3-(difluoroboryl)-5,6-dimethyl-2,2'-bipyridine (11).

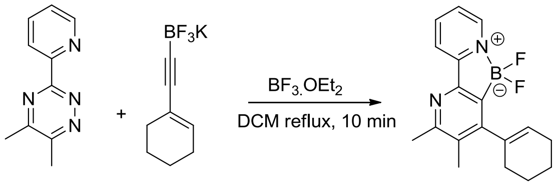

Following the general procedure, a solution of 5,6-dimethyl-3-(pyridin-2-yl)-1,2,4-triazine (40 mg, 0.215 mmol) and potassium (cyclohex-1-en-1-ylethynyl)trifluoroborate (137 mg, 0.645 mmol) in DCM (2 mL) was treated with $BF_3.OEt_2$ (80 µL, 0.645 mmol). Chromatographic purification over silica gel (gradient; starting with petroleum ether, ending with ethyl acetate) afforded 4-(cyclohex-1-en-1-yl)-3-(difluoroboryl)-5,6-dimethyl-2,2'-bipyridine (40 mg, 59%) as a colourless solid M.p 176-179 ^oC. ^1H NMR (250 Hz, $CDCl_3$) δ 1.72-1.88 (4H, m, CH_2), 2.21-2.30 (7H, m, CH_2, CH_3), 2.59 (3H, s, CH_3) 5.64-5.67 (1H, m, CH), 7.53-7.57 (1H, m, Ar), 8.17 (1H, td, J = 1.5, 8.0 Hz), 8.29 (1H, d, J = 8.0 Hz), 8.52 (1H, d, J = 5.5 Hz). ^{13}C NMR (62.9 MHz, $CDCl_3$) δ 15.9, 22.2, 22.9, 23.4, 25.2, 29.2, 118.4, 124.1, 125.8, 131.9, 136.8, 141.1, 143.6, 152.0, 155.6, 155.8, 158.5 ^{19}F NMR (235 MHz, $CDCl_3$) δ -159.9. FTIR, Thin Film. 2927 (m), 2856 (w), 1626 (s), 1560 (s), 1487 (s), 1447 (m), 1385 (m), 1256 (m), 1079 (s), 1030 (s), 945 (w) cm^{-1}. (ESI) [MH^+] calcd for $C_{18}H_{20}{}^{11}BN_2F_2$: 313.1685 found 313.1688.

Synthesis of ethyl 3-(difluoroboryl)-4,6-diphenyl-[2,2'-bipyridine]-5-carboxylate (12).

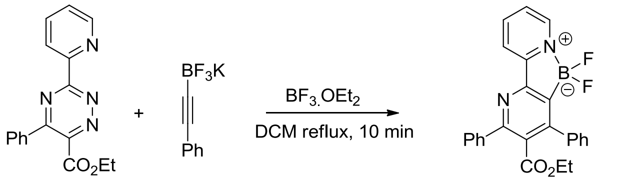

Following the general procedure, a solution of ethyl 5-phenyl-3-(pyridin-2-yl)-1,2,4-triazine-6-carboxylate (40 mg, 0.131 mmol) and potassium (phenylethynyl)trifluoroborate (82 mg, 0.393 mmol) in DCM (2 mL) was treated with $BF_3.OEt_2$ (50 µL, 0.393 mmol). Chromatographic purification over silica gel (gradient; starting with petroleum ether, ending with ethyl acetate) afforded ethyl 3-(difluoroboryl)-4,6-diphenyl-[2,2'-bipyridine]-5-carboxylate (46 mg, 82%) as a colourless solid. M.p 190-191 °C. ^1H NMR (250 MHz, $CDCl_3$): δ 0.88 (3H, t, J = 7.0 Hz, CO_2Et), 3.96 (2H, q, J = 7.0 Hz, CO_2Et), 7.42-7.52 (6H, m, Ar), 7.58-7.65 (2H, m, Ar), 7.67-7.76 (3H, m, Ar), 8.27 (1H, td, J = 1.5, 7.5 Hz, Ar), 8.47 (1H, d, J = 8.0 Hz, Ar), 8.61 (1H, d, J = 5.5 Hz, Ar). ^{13}C NMR (100.6 MHz, $CDCl_3$): δ 13.4, 61.4, 119.6, 125.6, 128.3 (x2), 126.4, 128.5, 128.6, 129.0, 130.9, 138.0, 139.6, 141.5, 144.1, 152.2, 154.3, 155.4 158.0, 168.8. ^{19}F NMR (235.1 MHz, $CDCl_3$): δ -156.4. FTIR: 1725 (s), 1626 (m), 1552 (s), 1490 (s), 1240 (s), 1187 (s), 1141 (s), 1107 (m), 1074 (s) cm^{-1}. HRMS: (ESI) [MH^+] calcd for $C_{25}H_{20}{}^{11}BN_2O_2F_2$: 429.1586, found 429.1594.

Synthesis of ethyl 4-butyl-3-(difluoroboryl)-6-phenyl-[2,2'-bipyridine]-5-carboxylate (13).

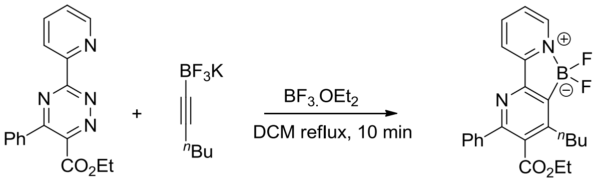

Following the general procedure, a solution of ethyl 5-phenyl-3-(pyridin-2-yl)-1,2,4-triazine-6-carboxylate (40 mg, 0.131 mmol) and potassium (hex-1-ynyl)trifluoroborate (74 mg, 0.393 mmol) in DCM (2 mL) was treated with $BF_3.OEt_2$ (50 µL, 0.393 mmol). Chromatographic purification over silica gel (gradient; starting with petroleum ether, ending with ethyl acetate) afforded ethyl 4-butyl-3-(difluoroboryl)-6-phenyl-[2,2'-bipyridine]-5-carboxylate (33 mg, 62%) as a colourless solid. M.p 143-144 °C. ^{1}H NMR (250 MHz, $CDCl_3$): δ 0.80-1.10 (6H, m, 2 x CH_3), 1.49 (2H, q, *J* = 7.5 Hz, CH_2), 1.68-1.75 (2H, m, CH_2), 2.90 (2H, m, CH_2), 4.16 (2H, q, *J* = 7.5 Hz, CH_2), 7.42-7.50 (3H, m, Ar), 7.63-7.72 (3H, m, Ar), 8.24 (1H, dt, *J* = 1.5, 8.0 Hz, Ar), 8.41 (1H, d, *J* = 8.0 Hz, Ar), 8.63 (1H, d, *J* = 5.5 Hz, Ar). ^{13}C NMR (100.6 MHz, $CDCl_3$): δ 13.7, 13.9, 23.1, 33.2, 33.3, 61.4, 119.5, 125.3, 128.3, 128.4, 128.8, 131.1, 140.0, 141.4, 144.1, 154.2, 154.7, 155.1, 158.0, 169.1. ^{19}F NMR (235.1 MHz, $CDCl_3$): δ -159.1. FTIR: 2958 (m), 2872 (w), 1723 (s), 1627 (m), 1561 (s), 1489 (s), 1248 (m), 1160 (s), 1096 (s) cm^{-1}. HRMS: (ESI) [MH^+] calcd for $C_{23}H_{24}{}^{11}BN_2O_2F_2$: 409.1899, found 409.1894.

Synthesis of 3-(difluoroboryl)-4-phenyl-2,2'-bipyridine (14).

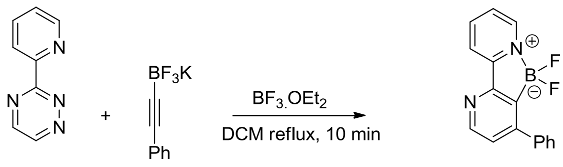

Following the general procedure, a solution of 3-(pyridin-2-yl)-1,2,4-triazine (40 mg, 0.316 mmol) and potassium (phenylethynyl)trifluoroborate (197 mg, 0.948 mmol) in DCM (2 mL) was treated with $BF_3.OEt_2$ (116 µL, 0.948 mmol). Chromatographic purification over silica gel (gradient; starting with petroleum ether, ending with ethyl acetate) afforded 3-(difluoroboryl)-4-phenyl-2,2'-bipyridine (44 mg, 50%) as a colourless solid. M.p 172-173 °C. ^{1}H NMR (400 MHz, $CDCl_3$): δ 7.43-7.59 (4H, m, Ar), 7.64-7.69 (1H, m, Ar), 7.94 (2H, d, *J* = 7.0 Hz, Ar), 8.27 (1H, td, *J* = 1.5, 8.0 Hz, Ar), 8.39 (1H, d, *J* = 8.0 Hz, Ar), 8.63 (1H, d, *J* = 5.0 Hz, Ar), 8.67 (1H, d, *J* = 5.0 Hz, Ar). ^{13}C NMR (100.6 MHz, $CDCl_3$): δ 119.1, 125.1, 127.9, 128.3, 128.8, 131.9, 139.2,

141.2, 144.1, 150.6, 153.0, 155.2, 156.0. ^{19}F NMR (235.1 MHz, $CDCl_3$): δ -155.9. FTIR: 3059 (w), 1628 (m), 1569 (s), 1491 (m), 1250 (w), 1158 (w), 1101 (s) cm^{-1}. HRMS: (ESI) [MH^+] calcd for $C_{16}H_{12}{}^{11}BN_2F_2$: 281.1053, found 281.1062.

Synthesis of alternative 3-substituted-1,2,4-triazines

Synthesis of *N,N*-dimethyl-5-phenyl-1,2,4-triazine-3-carboxamide (1f).

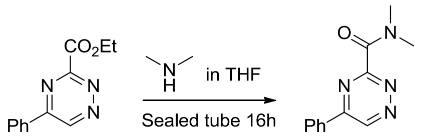

Ethyl 5-phenyl-1,2,4-triazine-3-carboxylate (500 mg, 2.18 mmol) and a solution of dimethylamine in THF (2 M) (8.72 mL, 17.44 mmol) in a sealed vessel were heated at 80 ^oC for 16 hours. Evaporation of the solvent followed by chromatographic purification over silica gel (gradient; starting with petroleum ether, ending with ethyl acetate) afforded *N,N*-dimethyl-5-phenyl-1,2,4-triazine-3-carboxamide (455 mg, 91%) as a yellow solid. M.p 114-115 °C. ^{1}H NMR (400 MHz, $CDCl_3$): δ 3.06 (3H, s, CH_3), 3.25 (3H, s, CH_3), 7.55-7.68 (3H, m, Ar), 8.22-8.27 (2H, m, Ar), 9.73 (1H, s, Ar). ^{13}C NMR (100.6 MHz, $CDCl_3$): δ 35.1, 38.5, 127.9, 129.5, 132.6, 133.2, 145.8, 156.0, 161.6, 165.0. FTIR: 2929 (w), 1652 (s), 1544 (m), 1496 (m), 1320 (w) cm^{-1}. HRMS: (ESI) [MH^+] calcd for $C_{12}H_{13}N_4O$: 229.1089, found 229.1080.

Synthesis of (5-phenyl-1,2,4-triazin-3-yl)(piperidin-1-yl)methanone (1g).

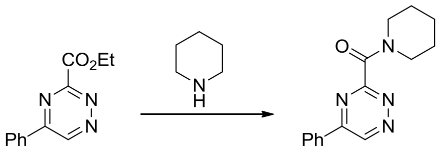

Ethyl 5-phenyl-1,2,4-triazine-3-carboxylate (619 mg, 0.270 mmol) was dissolved in piperidine (5 mL) and the mixture was heated to 40 ^oC for 2 hours. Evaporation of the solvent followed by chromatographic purification over silica gel (gradient; starting with petroleum ether, ending with ethyl acetate) afforded (5-phenyl-1,2,4-triazin-3-yl)(piperidin-1-yl)methanone as a dark solid (608 mg, 84%). M.p 88-90 °C. ^{1}H NMR (400 MHz, $CDCl_3$): δ 1.62-1.64 (2H, m, CH_2), 1.71-1.75 (4H, m, CH_2), 3.30-3.32 (2H, m, CH_2), 3.80-3.83 (2H, m, CH_2), 7.54-7.62 (3H, m, Ar), 8.20-8.23 (2H, m, Ar), 9.69 (1H, s, Ar-H). ^{13}C NMR (100.6 MHz, $CDCl_3$): δ 24.4, 25.4, 26.3,

43.0, 48.2, 127.9, 129.5, 132.7, 133.1, 145.7, 155.9, 161.8, 163.5. FTIR: 3483 (w), 2940 (m), 2858 (w), 1646 (s), 1544 (s), 1481 (s), 1441 (w), 1320 (w), 1222 (m), 1135 (w), 1011 (w) cm^{-1}. (ESI) [MH^+] calcd for $C_{15}H_{17}N_4O$: 269.1402 found 269.1401.

Synthesis of (1,2,4-triazin-3-yl)(piperidin-1-yl)methanone (1h).

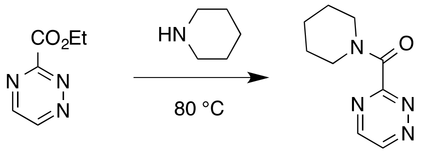

Ethyl 1,2,4-triazine-3-carboxylate (400 mg, 2.61 mmol) was dissolved in piperidine (4.0 mL) and the mixture was heated to 80 °C for 3 hours. Evaporation of the solvent followed by chromatographic purification over silica gel (gradient; starting with dichloromethane, ending with ethyl acetate) afforded (1,2,4-triazin-3-yl)(piperidin-1-yl)methanone as a yellow oil (295 mg, 87%). M.p 162-164 °C. ^{1}H NMR (400 MHz, $CDCl_3$): δ 1.61-1.62 (2H, m, CH_2), 1.71-1.73 (4H, m, CH_2), 3.26-3.29 (2H, m, CH_2), 3.78-3.81 (2H, m, CH_2), 8.73 (1H, d, J = 2.5 Hz, Ar), 9.29 (1H, d, J = 2.5 Hz, Ar). ^{13}C NMR (100.6 MHz, $CDCl_3$): δ 24.4, 25.3, 26.3, 43.1, 48.2, 149.3, 149.4, 162.5, 163.0. FTIR: 2497 (w), 2939 (m), 2858 (m), 1646 (s), 1448 (m), 1395 (m), 1345 (m), 1298 (m), 1209 (m), 1132 (m) cm^{-1}. (ESI) [MH^+] calcd for $C_{14}H_{11}N_4$: 235.0984 found 235.0981.

Synthesis of 3-phenyl-6-(2-pyridyl)-1,2,4-triazine (25).

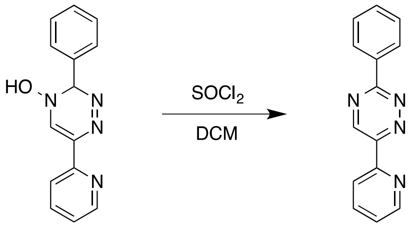

4-hydroxy-3-hydro-3-phenyl-6-(2-pyridyl)-1,2,4-triazine (401 mg, 1.59 mmol) and thionyl chloride (0.12 mL, 1.59 mmol) were dissolved in dry dichloromethane (7.0 mL) and the mixture was stirred at RT for 1.5 hours. The solution was neutralized with $NaHCO_3$, extracted with DCM and washed with water and brine. The organic layers were combined and evaporated under reduced pressure. Chromatographic purification over silica gel (gradient; starting with petroleum ether, ending with ethyl acetate) afforded 3-phenyl-6-(2-pyridyl)-1,2,4-triazine as a yellow solid (110 mg, 30%). M.p 162-164 °C. ^{1}H NMR (400 MHz, $CDCl_3$): δ 7.42-7.46 (1H, m, Ar), 7.56-

7.58 (3H, m, Ar), 7.90-7.94 (1H, m, Ar), 8.60-8.63 (2H, m, Ar), 8.65-8.67 (1H, m, Ar), 8.75-8.77 (1H, m, Ar), 9.69 (1H, s, Ar). ^{13}C NMR (100.6 MHz, $CDCl_3$): δ 121.5, 125.2, 128.4, 129.0, 131.9, 134.7, 137.4, 148.0, 149.7, 151.7, 154.1, 163.5. FTIR: 3062 (w), 1587 (m), 1404 (s), 1086 (w), 787 (w), 690 (m) cm^{-1}. (ESI) [MH^+] calcd for $C_9H_{13}N_4O$: 193.1089 found 193.1080.

Cycloaddition on alternative 3-substituted-1,2,4-triazines

Synthesis of 3-(difluoroboryl)-*N*,*N*-dimethyl-4,6-diphenylpicolinamide (16).

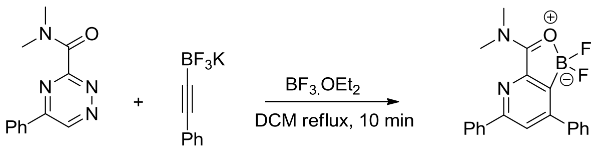

Following the general procedure, a solution of *N*,*N*-dimethyl-5-phenyl-1,2,4-triazine-3-carboxamide (50 mg, 0.219 mmol) and potassium (phenylethynyl)trifluoroborate (137 mg, 0.657 mmol) in DCM (2 mL) was treated with $BF_3.OEt_2$ (83 µL, 0.657 mmol). Chromatographic purification over silica gel (gradient; starting with petroleum ether, ending with ethyl acetate) afforded 3-(difluoroboryl)-*N*,*N*-dimethyl-4,6-diphenylpicolinamide (46 mg, 60%) as a colourless solid. M.p 151-152 °C. ^{1}H NMR (400 MHz, $CDCl_3$): δ 3.53 (3H, s, CH_3), 4.28 (3H, s, CH_3), 7.45-7.59 (6H, m, Ar), 7.95-7.98 (2H, m, Ar), 8.00-8.05 (3H, m, Ar). ^{13}C NMR (100.6 MHz, $CDCl_3$): δ 39.4, 40.7, 122.8, 127.0, 128.3, 128.9, 129.0, 129.2, 129.6, 138.5, 138.7, 152.8, 153.6, 158.2, 170.5. ^{19}F NMR (235.1 MHz, $CDCl_3$): δ -149.1. FTIR: 2930 (w), 1662 (s), 1586 (m), 1575 (m), 1468 (m), 1268 (m), 1104 (s), 1009 (s) cm^{-1}. HRMS: (ESI) [MH^+] calcd for $C_{20}H_{18}{}^{11}BN_2OF_2$: 351.1480, found 351.1487.

Synthesis of 4-(cyclohex-1-en-1-yl)-3-(difluoroboryl)-*N*,*N*-dimethyl-6-phenylpicolinamide (17).

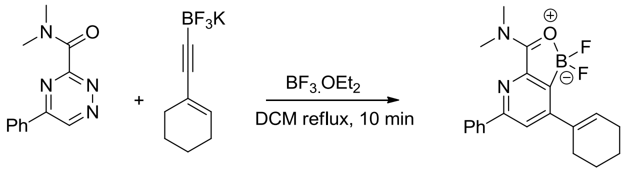

Following the general procedure, a solution of *N*,*N*-dimethyl-5-phenyl-1,2,4-triazine-3-carboxamide (322 mg, 1.41 mmol) and potassium (cyclohex-1-en-1-ylethynyl)trifluoroborate (897 mg, 4.235 mmol) in DCM (15 mL) was treated with $BF_3.OEt_2$ (522 µL, 4.235 mmol).

Chromatographic purification over silica gel (gradient; starting with petroleum ether, ending with ethyl acetate) afforded 4-(cyclohex-1-en-1-yl)-3-(difluoroboryl)-*N*,*N*-dimethyl-6-phenylpicolinamide (350 mg, 70%) as a colourless solid. M.p 208-209 °C. ^{1}H NMR (400 MHz, $CDCl_3$): δ 1.69-1.77 (2H, m, CH_2), 1.81-1.89 (2H, m, CH_2), 2.30-2.38 (2H, m, CH_2), 2.54-2.61 (2H, m, CH_2), 3.51 (3H, s, CH_3), 4.26 (3H, s, CH_3), 6.68-6.74 (1H, m, CH), 7.44-7.54 (3H, m, Ar), 7.80 (1H, s, Ar-H), 7.95-8.00 (2H, m, Ar). ^{13}C NMR (100.6 MHz, $CDCl_3$): δ 21.8, 22.9, 26.2, 27.2, 39.4, 40.6, 120.4, 126.9, 128.8, 129.3, 131.6, 135.7, 138.9, 152.3, 155.5, 157.8, 170.8. ^{19}F NMR (235.1 MHz, $CDCl_3$): δ -150.1. FTIR: 3030 (w), 2950 (m), 3837 (w), 1677 (m), 1606 (m), 1505 (s), 1455 (m), 1442 (m), 1347 (m), 1291 (m), 1246 (m), 1191 (m), 1164 (m), 1131 (m) cm^{-1}. HRMS: (ESI) [MH^+] calcd for $C_{20}H_{22}{}^{11}BN_2OF_2$: 355.1793, found 355.1776.

Synthesis of (3-(difluoroboryl)-4,6-diphenylpyridin-2-yl)(piperidin-1-yl)methanone (18).

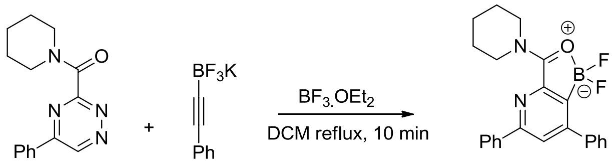

Following the general procedure, a solution of (5-phenyl-1,2,4-triazin-3-yl)(piperidin-1-yl)methanone (50 mg, 0.19 mmol) and potassium (phenylethynyl)trifluoroborate (116 mg, 0.56 mmol) in DCM (2.0 mL) was treated with $BF_3.OEt_2$ (0.07 mL, 0.56 mmol). Trituration in DCM/petroleum ether afforded (3-(difluoroboryl)-4,6-diphenylpyridin-2-yl)(piperidin-1-yl)methanone (62 mg, 84%) as a brown solid. M.p 166-168 °C. ^{1}H NMR (400 MHz, $CDCl_3$): δ 1.85-1.93 (6H, m, CH_2), 4.03-4.05 (2H, m, CH_2), 5.20-5.22 (2H, m, CH_2), 7.46-7.55 (6H, m, Ar), 7.93-7.99 (4H, m, Ar), 8.01 (1H, s, Ar). ^{13}C NMR (100.6 MHz, $CDCl_3$): δ 23.8, 26.1, 26.6, 48.0, 48.9, 122.8, 127.0, 128.4, 129.0 (x2), 129.1, 129.6, 138.6, 138.8, 153.2, 153.6, 158.0, 168.3. ^{19}F NMR (235.1 MHz, $CDCl_3$): δ -149.2. FTIR: 3033 (w), 2940 (m), 2866 (w), 1642 (s), 1586 (m), 1575 (m), 1444 (m), 1250 (m), 1177 (m), 1110 (s) cm^{-1}. HRMS: (ESI) [MH^+] calcd for $C_{23}H_{22}{}^{11}BN_2OF_2$: 391.1793, found 391.1783.

Synthesis of (3-(difluoroboryl)-4-butyl-6-phenylpyridin-2-yl)(piperidin-1-yl)methanone (19).

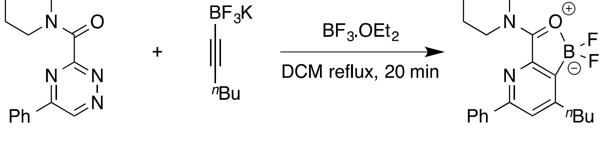

Following the general procedure, a solution of (5-phenyl-1,2,4-triazin-3-yl)(piperidin-1-yl)methanone (50 mg, 0.19 mmol) and potassium (hex-1-ynyl)trifluoroborate (105 mg, 0.56 mmol) in DCM (2.0 mL) was treated with $BF_3.OEt_2$ (0.07 mL, 0.56 mmol) for 20 min. Chromatographic purification over florisil (gradient; starting with petroleum ether, ending with ethyl acetate) afforded (3-(difluoroboryl)-4-butyl-6-phenylpyridin-2-yl)(piperidin-1-yl)methanone (41 mg, 58%) as a white solid. M.p 112-114 °C. ^{1}H NMR (400 MHz, $CDCl_3$): δ 0.94-0.98 (3H, m, CH_3), 1.14-1.47 (2H, m, CH_2), 1.70-1.78 (2H, m, CH_2), 1.82-1.89 (6H, m, CH_2), 2.86-2.90 (2H, m, CH_2), 3.98-4.01 (2H, m, CH_2), 5.15-5.17 (2H, m, CH_2), 7.43-7.52 (3H, m, Ar), 7.66 (1H, s, Ar), 7.91-7.93 (2H, m, Ar). ^{13}C NMR (100.6 MHz, $CDCl_3$): δ 14.0, 22.6, 23.8, 26.1, 26.5, 32.5, 34.9, 47.8, 48.8, 123.8, 126.9, 128.9, 129.3, 138.9, 152.3, 156.6, 157.7, 168.5. ^{19}F NMR (235.1 MHz, $CDCl_3$): δ -152.9. FTIR: 3034 (w), 2929 (m), 2862 (w), 1644 (s), 1589 (m), 1444 (m), 1265 (w), 1174 (m), 1107 (m) cm^{-1}. HRMS: (ESI) [MH^+] calcd for $C_{21}H_{26}{}^{11}BN_2OF_2$: 371.2106, found 371.2090.

Synthesis of (3-(difluoroboryl)-6-phenylpyridin-2-yl)(piperidin-1-yl)methanone (20).

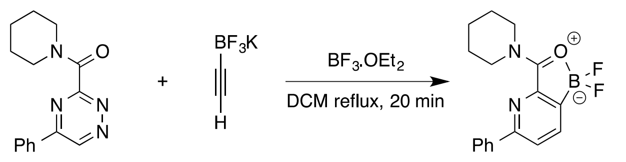

Following the general procedure, a solution of (5-phenyl-1,2,4-triazin-3-yl)(piperidin-1-yl)methanone (50 mg, 0.19 mmol) and potassium ethynyltrifluoroborate (74 mg, 0.56 mmol) in DCM (2.0 mL) was treated with $BF_3.OEt_2$ (0.07 mL, 0.56 mmol) for 20 min. Trituration in DCM/petroleum ether afforded (3-(difluoroboryl)-6-phenylpyridin-2-yl)(piperidin-1-yl)methanone (40 mg, 67%) as a white solid. M.p 214-216 °C. ^{1}H NMR (400 MHz, $CDCl_3$): δ 1.85-1.90 (6H, m, CH_2), 3.99-4.01 (2H, m, CH_2), 5.13-5.16 (2H, m, CH_2), 7.46-7.52 (3H, m, Ar), 7.86 (1H, d, J = 8.0 Hz, Ar), 7.91-7.94 (2H, m, Ar), 8,15 (1H, d, J = 8.0 Hz, Ar). ^{13}C NMR (100.6 MHz, $CDCl_3$): δ 23.7, 26.0, 26.5, 47.8, 48.8, 124.1, 126.9, 129.0, 129.6, 138.5, 139.2, 152.6, 157.4, 168.2. ^{19}F NMR (235.1 MHz, $CDCl_3$): δ -154.0. FTIR: 3426 (w), 3027 (w), 2951 (w), 2862 (w), 1646 (s), 1584 (m), 1442 (m), 1353 (m), 1271 (m), 1216 (m), 1121 (s) cm^{-1}. HRMS: (ESI) [MH^+] calcd for $C_{17}H_{18}{}^{11}BN_2OF_2$: 315.1480, found 315.1482.

Synthesis of (3-(difluoroboryl)-4-phenylpyridin-2-yl)(piperidin-1-yl)methanone (21).

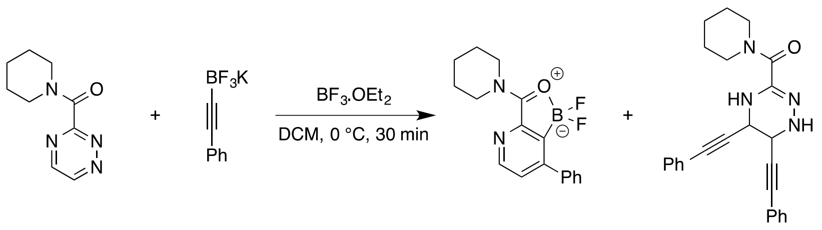

Following the general procedure, a solution of (1,2,4-triazin-3-yl)(piperidin-1-yl)methanone (20 mg, 0.15 mmol) and potassium (phenylethynyl)trifluoroborate (96 mg, 0.46 mmol) in DCM (2.0 mL) was treated with $BF_3.OEt_2$ (0.06 mL, 0.46 mmol) for 30 min at 0 °C. Chromatographic purification over florisil (gradient; starting with dichloromethane, ending with ethyl acetate) afforded (3-(difluoroboryl)-4-phenylpyridin-2-yl)(piperidin-1-yl)methanone (6 mg, 13%) as a white solid. M.p 168-170 °C. ^{1}H NMR (400 MHz, $CDCl_3$): δ 1.84-1.87 (6H, m, CH_2), 3.99-4.02 (2H, m, CH_2), 5.05-5.08 (2H, m, CH_2), 7.44-7.56 (4H, m, Ar), 7.88-7.90 (2H, m, Ar), 8.62-8.63 (1H, m, Ar). ^{13}C NMR (100.6 MHz, $CDCl_3$): δ 23.8, 26.1, 26.5, 47.8, 48.8, 125.7, 128.4, 128.9, 129.1, 138.5, 149.6, 152.8, 153.0, 168.2. ^{19}F NMR (235.1 MHz, $CDCl_3$): δ -149.3. FTIR: 3033 (w), 2923 (m), 2851 (w), 1645 (s), 1573 (m), 1419 (m), 1306 (m), 1194 (w), 1111 (m) cm^{-1}. HRMS: (ESI) [MH^+] calcd for $C_{17}H_{18}{}^{11}BN_2OF_2$: 315.1480, found 315.1488.

Side product **24** (R=Ph) was isolated as a brown oil (13 mg, 22%). ^{1}H NMR (400 MHz, $CDCl_3$): δ 1.63-1.67 (6H, m, CH_2), 3.62-3.65 (2H, m, CH_2), 3.91 (1H, d, J = 7.0 Hz, CH), 3.97-4.03 (2H, m, CH_2), 4.71 (1H, d, J = 7.0 Hz, CH), 5.35 (1H, br, NH), 5.59 (1H, br, NH), 7.33-7.35 (6H, m, Ar), 7.47-7.49 (4H, m, Ar). ^{13}C NMR (100.6 MHz, $CDCl_3$): δ 24.6, 25.7, 26.7, 44.5, 47.2, 48.3, 50.0, 84.6, 84.9, 85.4, 86.0, 122.1, 122.2, 128.2, 128.3, 128.6, 128.7, 131.9, 132.1, 140.3, 161.4. FTIR: 3292 (w), 2937 (w), 2895 (w), 1617 (s), 1490 (m), 1443 (m), 1281 (w), 1026 (w), 757 (w), 691 (w) cm^{-1}. HRMS: (ESI) [MH^+] calcd for $C_{25}H_{25}N_4O$: 397.2028, found 397.2039.

Synthesis of (3-(difluoroboryl)-4-butylpyridin-2-yl)(piperidin-1-yl)methanone (22).

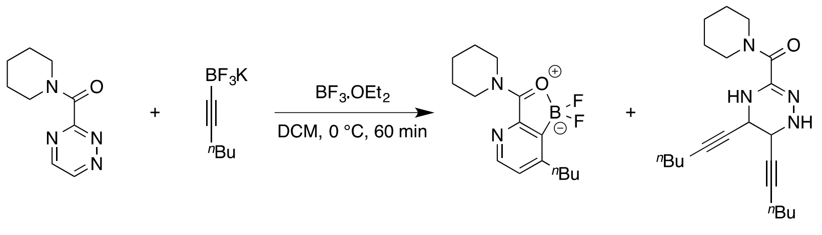

Following the general procedure, a solution of (1,2,4-triazin-3-yl)(piperidin-1-yl)methanone (20 mg, 0.15 mmol) and potassium (hex-1-ynyl)trifluoroborate (87 mg, 0.46 mmol) in DCM (2.0 mL) was treated with $BF_3.OEt_2$ (0.06 mL, 0.46 mmol) for 1h at 0 °C. Chromatographic purification over florisil (gradient; starting with dichloromethane, ending with ethyl acetate) afforded (3-(difluoroboryl)-4-butylpyridin-2-yl)(piperidin-1-yl)methanone (5 mg, 11%) as a colourless oil. ^{1}H NMR (400 MHz, $CDCl_3$): δ 0.92-0.95 (3H, m, CH_3), 1.36-1.42 (2H, m, CH_2), 1.66-1.70 (2H, m, CH_2), 1.82-1.83 (6H, m, CH_2), 2.79-2.83 (2H, m, CH_2), 3.95-3.98 (2H, m, CH_2), 5.00-5.03 (2H, m, CH_2), 7.20 (1H, d, *J* = 5.0 Hz, Ar), 8.45 (1H, d, *J* = 5.0 Hz, Ar). ^{13}C NMR (100.6 MHz, $CDCl_3$): δ 13.9, 22.4, 23.7, 26.0, 26.4, 32.3, 34.5, 47.6, 48.6, 126.7, 149.2, 152.1, 155.8, 168.4. ^{19}F NMR (235.1 MHz, $CDCl_3$): δ -153.2. FTIR: 3033 (w), 2929 (m), 2860 (m), 1643 (s), 1581 (m), 1419 (m), 1302 (m), 1187 (w), 1107 (m), 1009 (m) cm^{-1}. HRMS: (ESI) [MH^+] calcd for $C_{15}H_{22}{}^{11}BN_2OF_2$: 295.1793, found 295.1791.

Side product **24** (R=Bu) was isolated as a brown oil (13 mg, 24%) and tentatively assigned on the basis of ^{1}H NMR spectroscopy and HRMS. ^{1}H NMR (400 MHz, $CDCl_3$): δ 0.89-0.92 (6H, m, CH_3), 1.40-1.64 (14H, m, CH_2), 2.20-2.22 (4H, m, CH_2), 3.41-3.43 (1H, m, CH), 3.57 (2H, m, CH_2), 3.89-3.93 (2H, m, CH_2), 4.23-4.26 (1H, m, CH), 5.11 (1H, br, NH), 5.26 (1H, br, NH). HRMS: (ESI) [MH^+] calcd for $C_{21}H_{33}N_4O$: 357.2654, found 357.2646.

Synthesis of (3-(difluoroboryl)-pyridin-2-yl)(piperidin-1-yl)methanone (23).

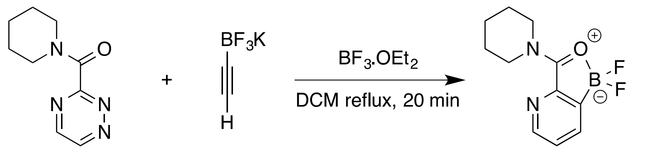

Following the general procedure, a solution of (1,2,4-triazin-3-yl)(piperidin-1-yl)methanone (20 mg, 0.15 mmol) and potassium ethynyltrifluoroborate (61 mg, 0.46 mmol) in DCM (2.0 mL) was treated with $BF_3.OEt_2$ (0.06 mL, 0.46 mmol) for 20 min. Trituration in DCM/petroleum ether afforded (3-(difluoroboryl)-pyridin-2-yl)(piperidin-1-yl)methanone (9 mg, 25%) as a yellow oil. ^{1}H NMR (400 MHz, $CDCl_3$): δ 1.83-1.85 (6H, m, CH_2), 3.96-3.99 (2H, m, CH_2), 5.00-5.02 (2H, m, CH_2), 7.41 (1H, dd, *J* = 5.0, 7.5 Hz, Ar), 8.09 (1H, dd, *J* = 1.5, 7.5 Hz, Ar), 8.58-8.59 (1H, dd, *J* = 1.5, 5.0 Hz, Ar). ^{13}C NMR (100.6 MHz, $CDCl_3$): δ 23.7, 26.0, 26.5, 47.6, 48.7, 127.1, 138.3, 149.2, 152.5, 168.1. ^{19}F NMR (235.1 MHz, $CDCl_3$): δ -154.2. FTIR: 3444 (m), 2928 (w), 2858 (w), 1646 (s), 1567 (w), 1419 (m), 1303 (m), 1265 (m), 1120 (m) cm^{-1}. HRMS: (ESI) [MH^+] calcd for $C_{11}H_{14}{}^{11}BN_2OF_2$: 239.1167, found 239.1177.

Synthesis of 4-(difluoroboryl)-2,3-diphenyl-5,2'-bipyridine (26)

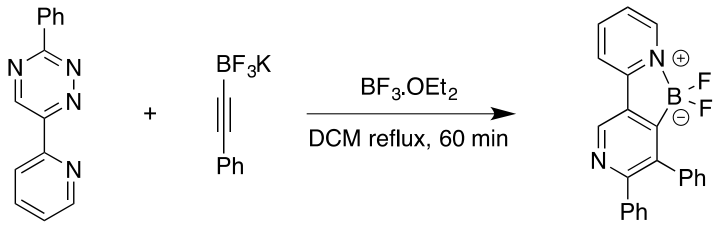

Following the general procedure, a solution of 3-phenyl-6-(2-pyridyl)-1,2,4-triazine (20 mg, 0.09 mmol) and potassium (phenylethynyl)trifluoroborate (53 mg, 0.26 mmol) in DCM (2 mL) was treated with $BF_3.OEt_2$ (0.03 mL, 0.26 mmol) for 1 hour. Chromatographic purification over silica gel (gradient; starting with petroleum ether, ending with ethyl acetate) afforded 4-(difluoroboryl)-2,3-diphenyl-5,2'-bipyridine (19 mg, 63%) as a white solid. M.p 222-224 °C. ^{1}H NMR (500 MHz, $CDCl_3$): δ 7.23-7.25 (3H, m, Ar), 7.27-7.31 (3H, m, Ar), 7.36-7.40 (4H, m, Ar), 7.55-7.57 (1H, m, Ar), 8.07-8.09 (1H, d, J = 8.0 Hz, Ar), 8.18-8.21 (1H, m, Ar), 8.54-8.55 (1H, d, J = 5.5 Hz, Ar), 9.08 (1H, s, Ar). ^{13}C NMR (125.8 MHz, $CDCl_3$): δ 118.5, 124.1, 127.1, 127.8, 127.9, 128.0, 130.0, 130.1, 131.6, 139.0, 139.1, 140.3, 141.4, 142.2, 143.8, 153.8, 160.2. ^{19}F NMR (376.5 MHz, $CDCl_3$): δ -155.7. FTIR: 3057 (w), 2924 (w), 1625 (s), 1575 (m), 1486 (s), 1386 (s), 1160 (m), 1099 (s), 1017 (m), 767 (s), 733 (s), 700 (s) cm^{-1}. HRMS: (ESI) [MH$^+$] calcd for $C_{22}H_{16}{}^{11}BF_2N_2$: 357.1375, found 357.1379.

Synthesis of 3'-(difluoroboryl)-4'-phenyl-2,2':5',2''-terpyridine (28)

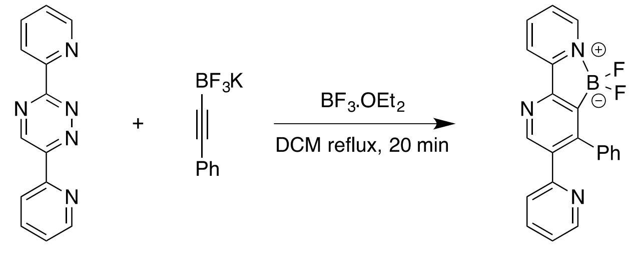

Following the general procedure, a solution of 3,6-bis(2-pyridyl)-1,2,4-triazine (50 mg, 0.21 mmol) and potassium (phenylethynyl)trifluoroborate (133 mg, 0.64 mmol) in DCM (2.0 mL) was treated with $BF_3.OEt_2$ (0.08 mL, 0.64 mmol). Chromatographic purification over silica gel (gradient; starting with dichloromethane, ending with ethyl acetate) afforded 3'-(difluoroboryl)-4'-phenyl-2,2':5',2''-terpyridine (31 mg, 41%) as a colourless solid. M.p 196-198 °C. ^{1}H NMR (400 MHz, $CDCl_3$): δ 6.90-6.93 (1H, m, Ar), 7.14-7.17 (1H, m, Ar), 7.29-7.30 (3H, m, Ar), 7.37-7.44 (3H, m, Ar), 7.61-7.64 (1H, m, Ar), 8.22-8.27 (1H, m, Ar), 8.39-8.41 (1H, m, Ar), 8.56-8.57 (1H, m, Ar), 8.67-8.69 (1H, m, Ar), 8.91 (1H, s, Ar). ^{13}C NMR (100.6 MHz, $CDCl_3$): δ 119.3,

122.0, 125.2, 125.9, 127.9, 128.2, 129.6, 135.5, 137.1, 138.3, 141.4, 144.2, 149.8, 151.6, 152.4, 154.8, 155.2, 156.7. ^{19}F NMR (235.1 MHz, $CDCl_3$): δ -156.4. FTIR: 3642 (w), 3059 (m), 2924 (m), 2227 (w), 1627 (s), 1588 (m), 1490 (s), 1439 (s), 1290 (m), 1135 (s), 1014 (s), 790 (m) cm^{-1}. HRMS: (ESI) [MH^+] calcd for $C_{21}H_{15}{}^{11}BN_3F_2$: 358.1327, found 358.1333.

Application of difluoroboryl-pyridines

Synthesis of 4-(cyclohex-1-en-1-yl)-6-phenyl-3-(p-tolyl)-2,2'-bipyridine (29).

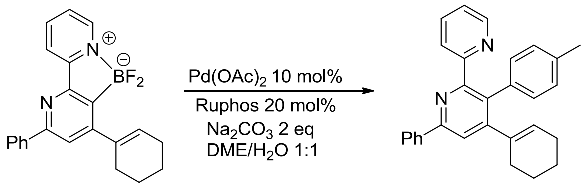

In a flame vessel equipped with a reflux condenser 4-(cyclohex-1-en-1-yl)-3-(difluoroboryl)-6-phenyl-2,2'-bipyridine was added (40 mg, 0.111 mmol), $Pd(OAc)_2$ (2.5 mg, 0.011 mmol), Ruphos (10 mg, 0.022 mmol), Na_2CO_3 (23 mg, 0.22 mmol) and 4-iodotoluene (48 mg, 0.222 mmol) in a 1:1 mixture of DME/H_2O (2 mL). The system was purged with argon and heated at reflux for 16 hours. The solvents were evaporated and the residue purified by column chromatography on silica gel. Chromatographic purification (gradient; starting with petroleum ether, ending with petroleum ether/EtOAc 8:2) afforded 4-(cyclohex-1-en-1-yl)-6-phenyl-3-(p-tolyl)-2,2'-bipyridine as a colorless oil (28 mg, 63%). ^{1}H NMR (400 MHz, $CDCl_3$): δ 1.41-1.47 (2H, m, CH_2), 1.49-1.55 (2H, m, CH_2), 1.69-1.77 (2H, m, CH_2), 2.08-2.15 (2H, m, CH_2), 2.31 (3H, s, CH_3), 5.77-5.81 (1H, m, CH), 6.97-7.03 (4H, m, Ar), 7.13 (1H, ddd, J = 1.0, 5.0, 8.5 Hz, Ar), 7.30 (1H, dt, J = 1.0, 8.0 Hz, Ar), 7.39-7.44 (1H, m, Ar), 7.45-7.51 (2H, m, Ar), 7.53 (1H, td, J = 2.0, 8.0 Hz, Ar), 7.65 (1H, s, Ar), 8.13 (2H, d, J = 7.0 Hz, Ar), 8.53 (1H, dq, J = 1.0, 5.0 Hz, Ar). ^{13}C NMR (100.6 MHz, $CDCl_3$): δ 21.2, 21.8, 22.7, 25.5, 28.7, 120.4, 121.8, 124.9, 127.2, 128.2, 128.6, 128.7 (x2), 130.4, 132.7, 135.0, 135.4, 136.1, 138.3, 139.4, 148.7, 153.8, 155.7, 156.7, 159.4. FTIR: 2929 (s), 1578 (m), 1531 (w), 1474 (w), 1417 (m), 1376 (w) cm^{-1}. HRMS: (ESI) [MH^+] calcd for $C_{29}H_{27}N_2$: 403.2174, found 403.2174.

Synthesis of 4-(cyclohex-1-en-1-yl)-*N*,*N*-dimethyl-3,6-diphenylpicolinamide (30).

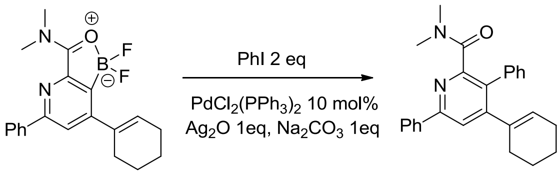

In a flame vessel equipped with a reflux condenser 4-(cyclohex-1-en-1-yl)-3-(difluoroboryl)-*N*,*N* -dimethyl-6-phenylpicolinamide was added (40 mg, 0.113 mmol), $PdCl_2(PPh_3)_2$ (8 mg, 0.0113 mmol), Ag_2O (26 mg, 0.113 mmol), Na_2CO_3 (12 mg, 0.113 mmol) and iodobenzene (46 mg, 0.216 mmol) in a 1:1 mixture of DME/H_2O (2 mL). The system was purged with nitrogen and heated at reflux for 2 hours. The solvents were then evaporated and the residue purified by column chromatography on silica gel. Chromatographic purification (gradient; starting with petroleum ether, ending with petroleum ether/EtOAc 1:1) afforded 4-(cyclohex-1-en-1-yl)-*N*,*N*-dimethyl-3,6-diphenylpicolinamide as a colorless solid (42 mg, 97%). M.p 142-143 °C. ^1H NMR (400 MHz, $CDCl_3$): δ 1.39-1.56 (4H, m, 2*CH_2), 1.71-1.79 (2H, m, CH_2), 2.08-2.15 (2H, m, CH_2), 2.64 (3H, s, CH_3), 2.85 (3H, s, CH_3), 5.80-5.84 (1H, m, CH), 7.31-7.52 (8H, m, Ar), 7.63 (1H, s, Ar-H), 8.09 (2H, d, J = 7.0 Hz, Ar). ^{13}C NMR (100.6 MHz, $CDCl_3$): δ 21.7, 22.6, 25.6, 28.5, 34.2, 37.8, 120.5, 127.2, 127.7, 127.9, 128.6, 129.0, 129.6, 129.7, 130.4, 136.3, 137.7, 138.7, 153.2, 154.1, 156.3, 169.2. FTIR: 3059 (w), 2930 (s), 2362 (w), 2237 (w), 1644 (s), 1497 (m), 1412 (m), 1142 (m) cm^{-1}. HRMS: (ESI) [MH^+] calcd for $C_{26}H_{27}N_2O$: 383.2123, found 383.2116

^{1}H NMR, ^{13}C NMR and NOE Spectra of Novel Compounds

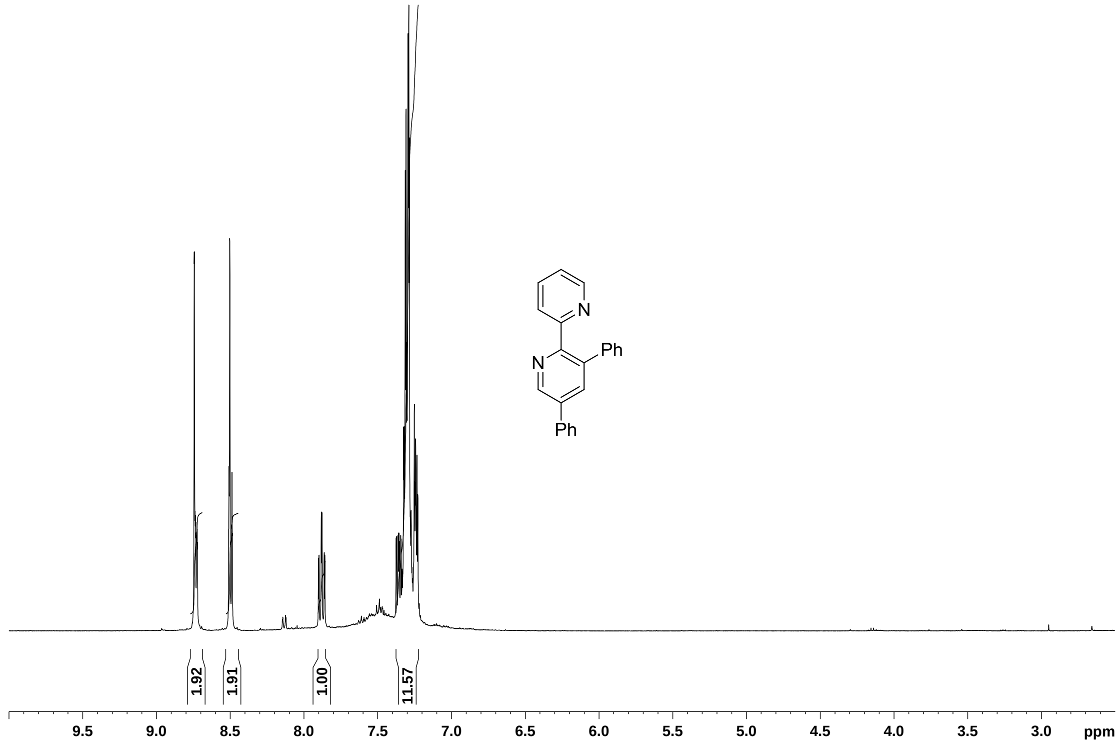

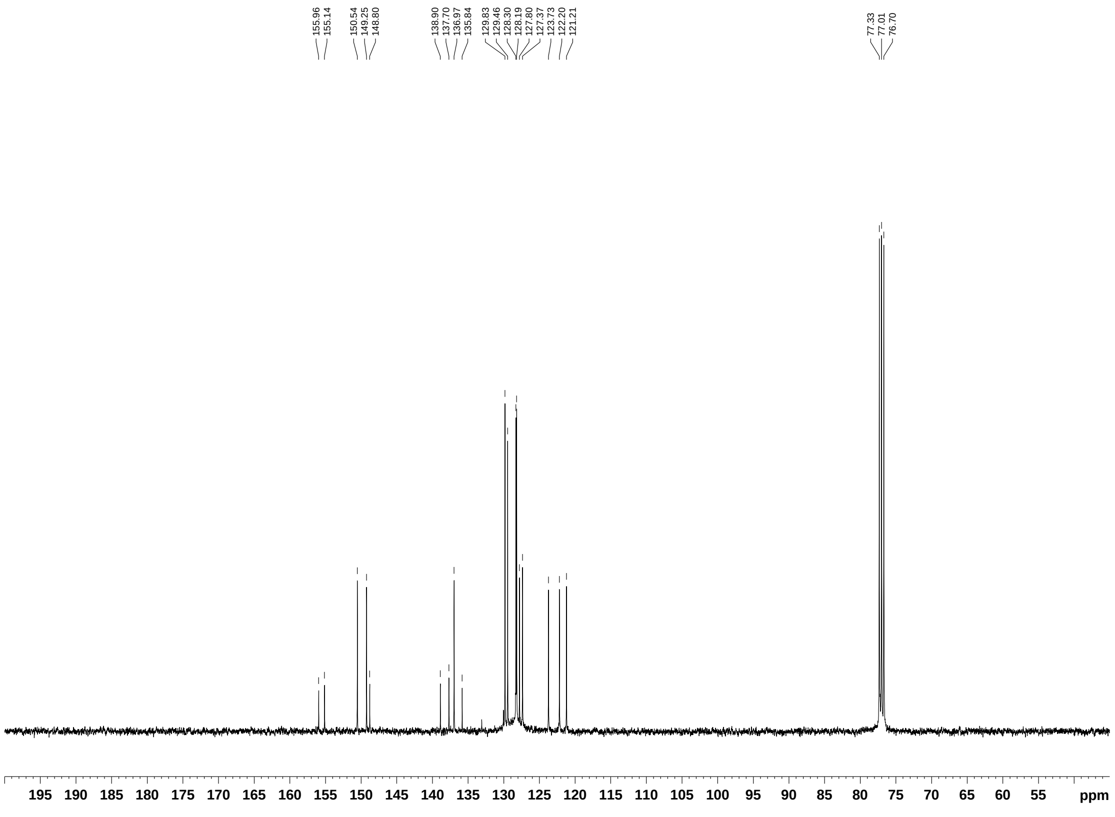

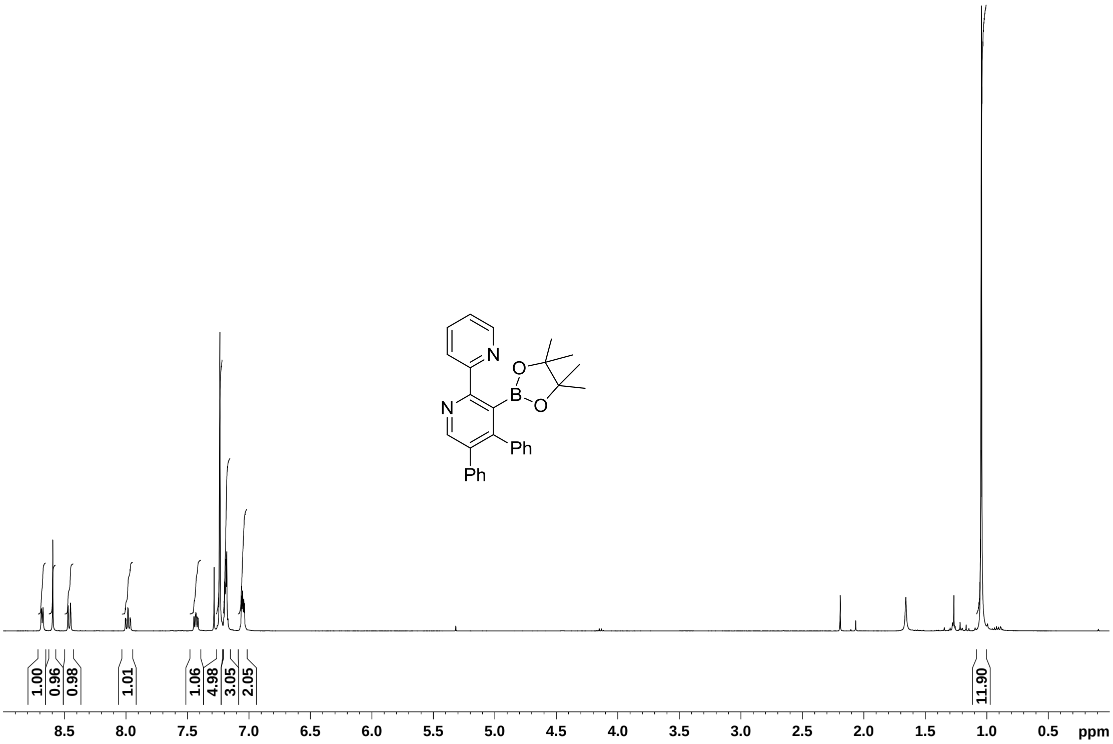
N
O
B
O
N
Ph
Ph
1.00
0.96
0.98
1.01
1.06
4.98
3.05
2.05
11.90
8.5 8.0 7.5 7.0 6.5 6.0 5.5 5.0 4.5 4.0 3.5 3.0 2.5 2.0 1.5 1.0 0.5 ppm

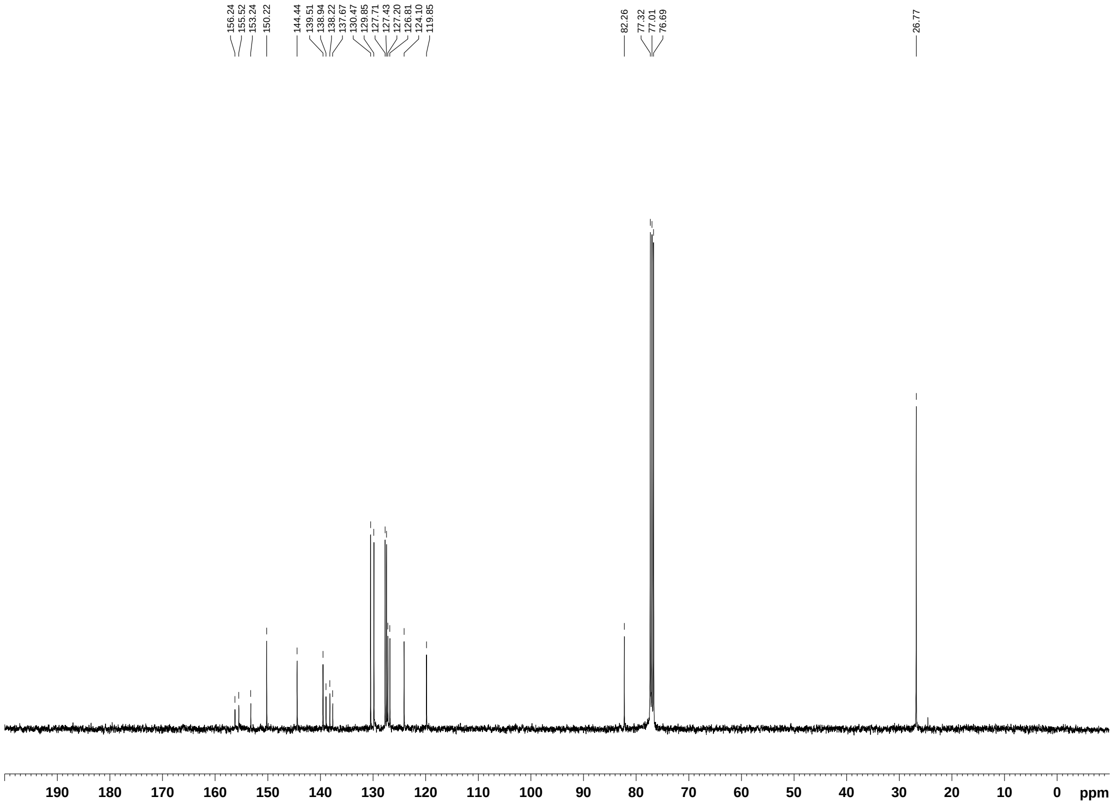
156.24
155.52
153.24
150.22
144.44
139.51
138.94
138.22
137.67
130.47
129.85
127.71
127.43
127.20
126.81
124.10
119.85
82.26
77.32
77.01
76.69
26.77
190 180 170 160 150 140 130 120 110 100 90 80 70 60 50 40 30 20 10 0 ppm

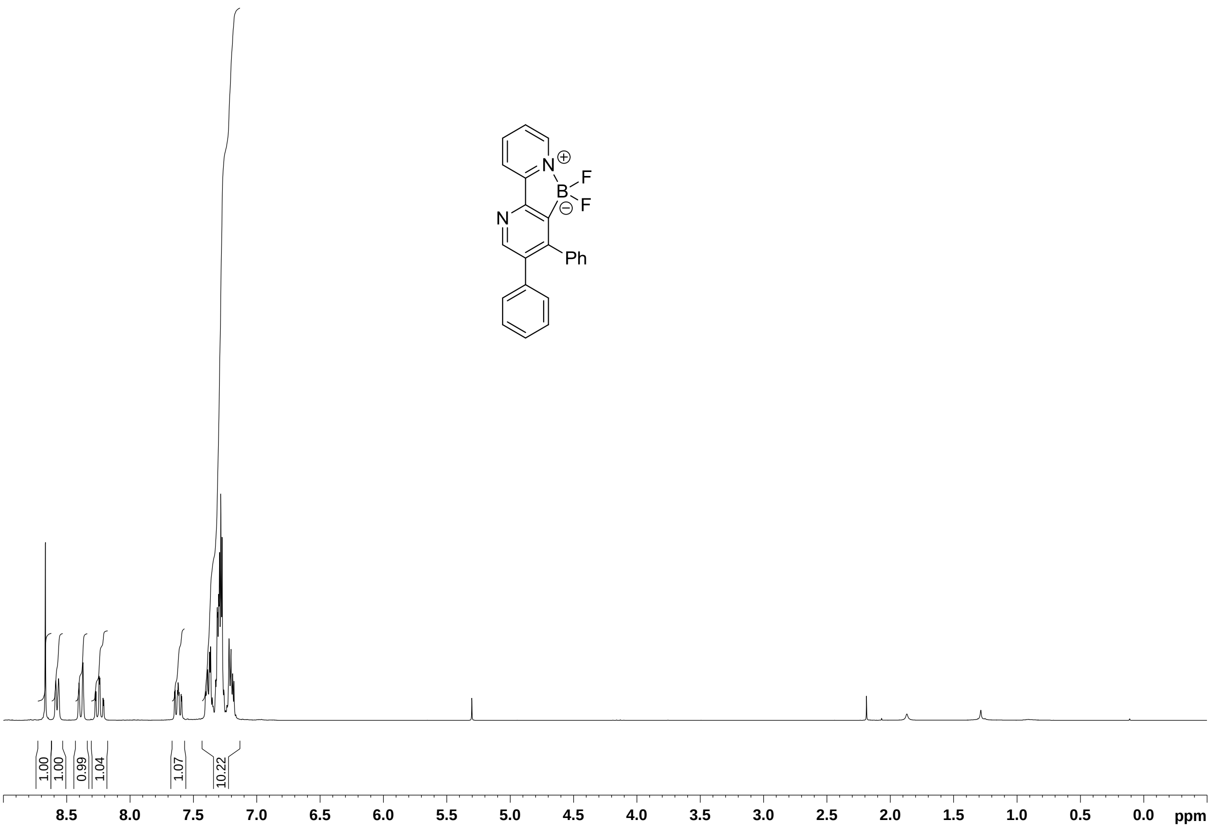
F
F
N
B
N
Ph
1.00
1.00
0.99
1.04
1.07
10.22
8.5 8.0 7.5 7.0 6.5 6.0 5.5 5.0 4.5 4.0 3.5 3.0 2.5 2.0 1.5 1.0 0.5 0.0 ppm

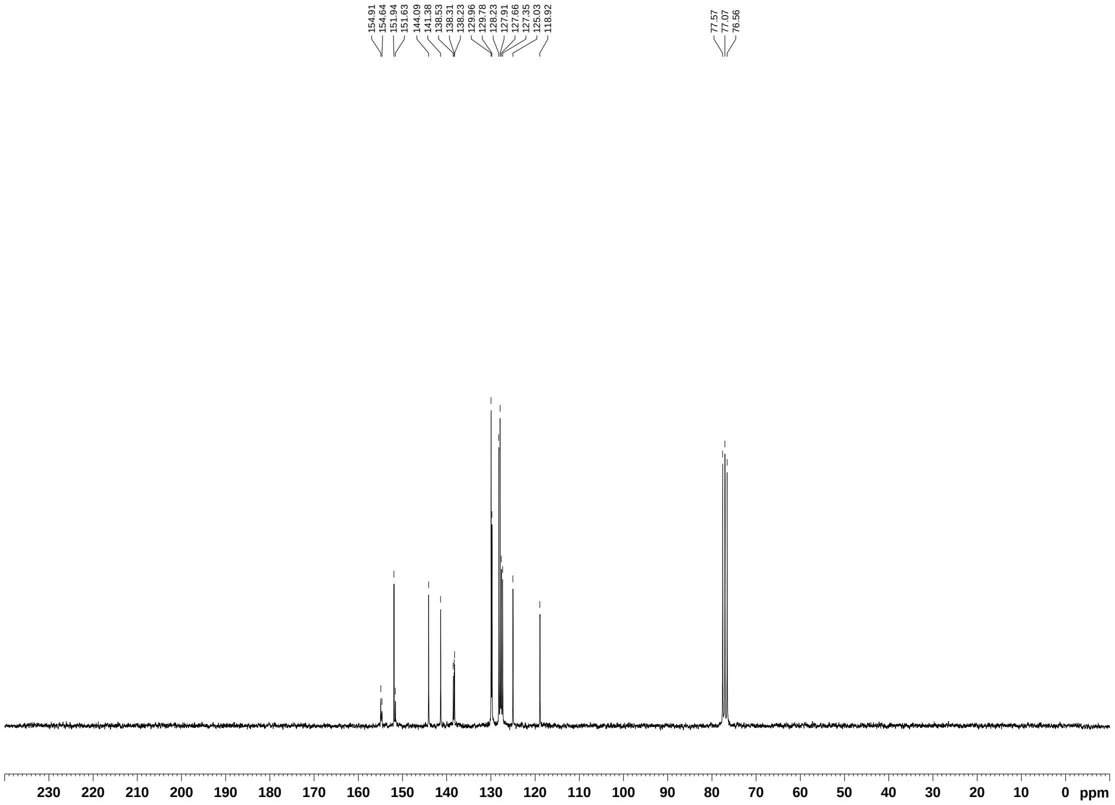
154.91
154.64
151.94
151.63
144.09
141.38
138.53
138.31
138.23
129.96
129.78
128.23
128.23
127.91
127.66
127.35
125.03
118.92
77.57
77.07
76.56
230 220 210 200 190 180 170 160 150 140 130 120 110 100 90 80 70 60 50 40 30 20 10 0 ppm

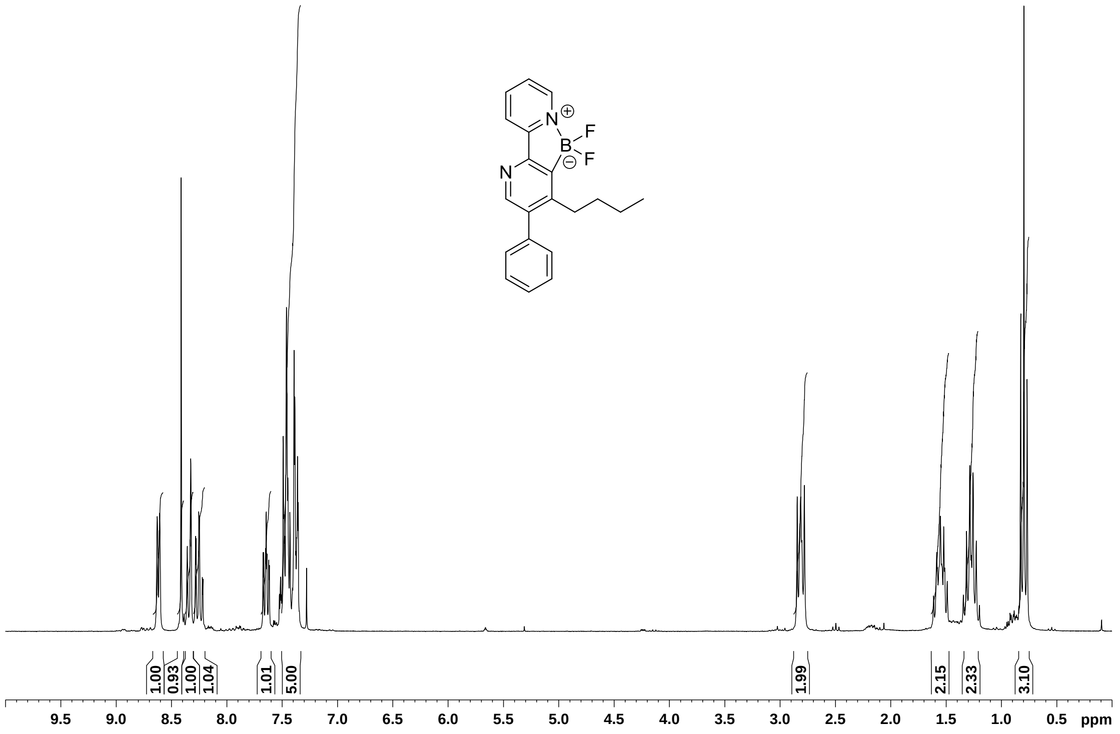
N
B
F
F
N
1.00
0.93
1.00
1.04
1.01
5.00
1.99
2.15
2.33
3.10
9.5 9.0 8.5 8.0 7.5 7.0 6.5 6.0 5.5 5.0 4.5 4.0 3.5 3.0 2.5 2.0 1.5 1.0 0.5 ppm

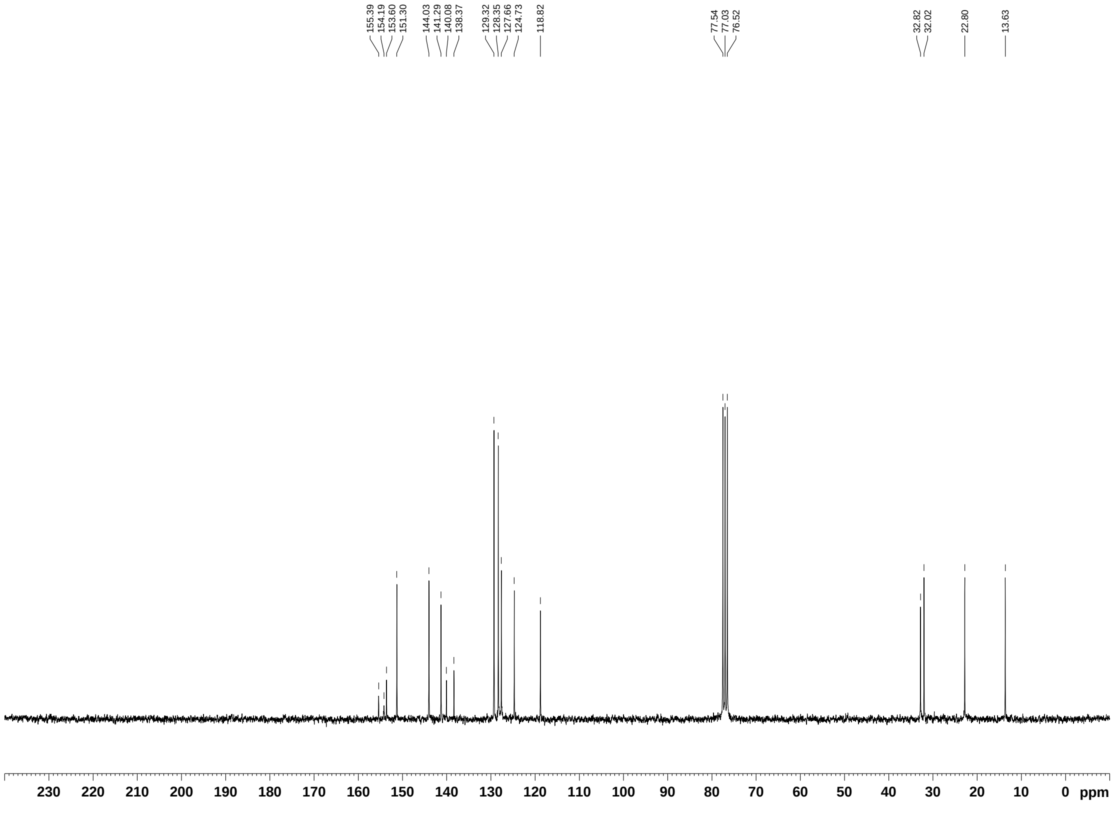
155.39
154.19
153.60
151.30
144.03
141.29
140.08
138.37
129.32
128.35
127.66
124.73
118.82
77.54
77.03
76.52
32.82
32.02
22.80
13.63
230 220 210 200 190 180 170 160 150 140 130 120 110 100 90 80 70 60 50 40 30 20 10 0 ppm

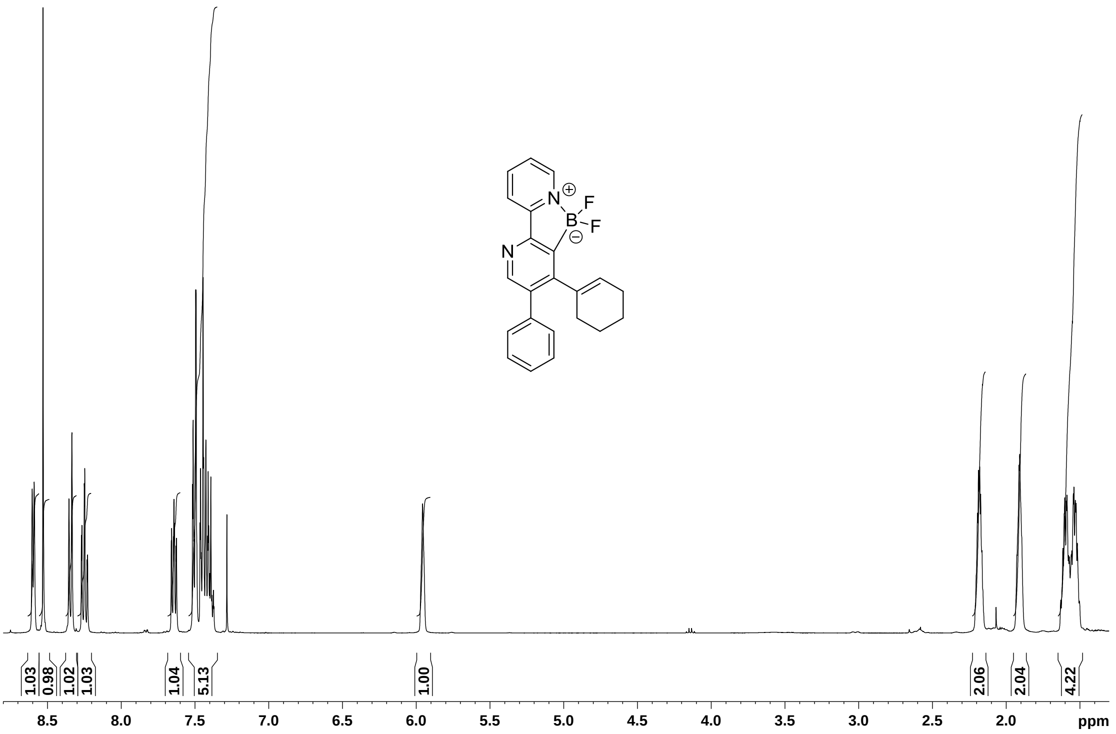
N
N
B
F
F
1.03
0.98
1.02
1.03
1.04
5.13
1.00
2.06
2.04
4.22
8.5
8.0
7.5
7.0
6.5
6.0
5.5
5.0
4.5
4.0
3.5
3.0
2.5
2.0
ppm

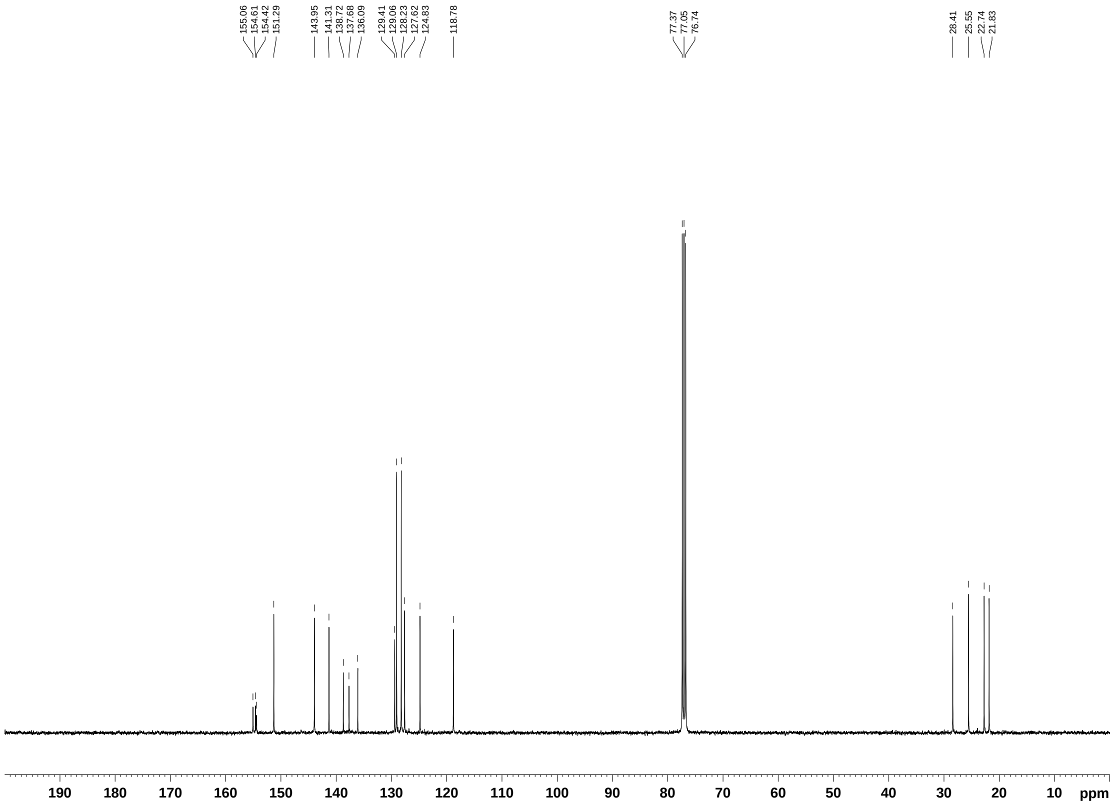
155.06
154.61
154.42
151.29
143.95
141.31
138.72
137.68
136.09
129.41
129.06
128.23
127.62
124.83
118.78
77.37
77.05
76.74
28.41
25.55
22.74
21.83
190
180
170
160
150
140
130
120
110
100
90
80
70
60
50
40
30
20
10
ppm

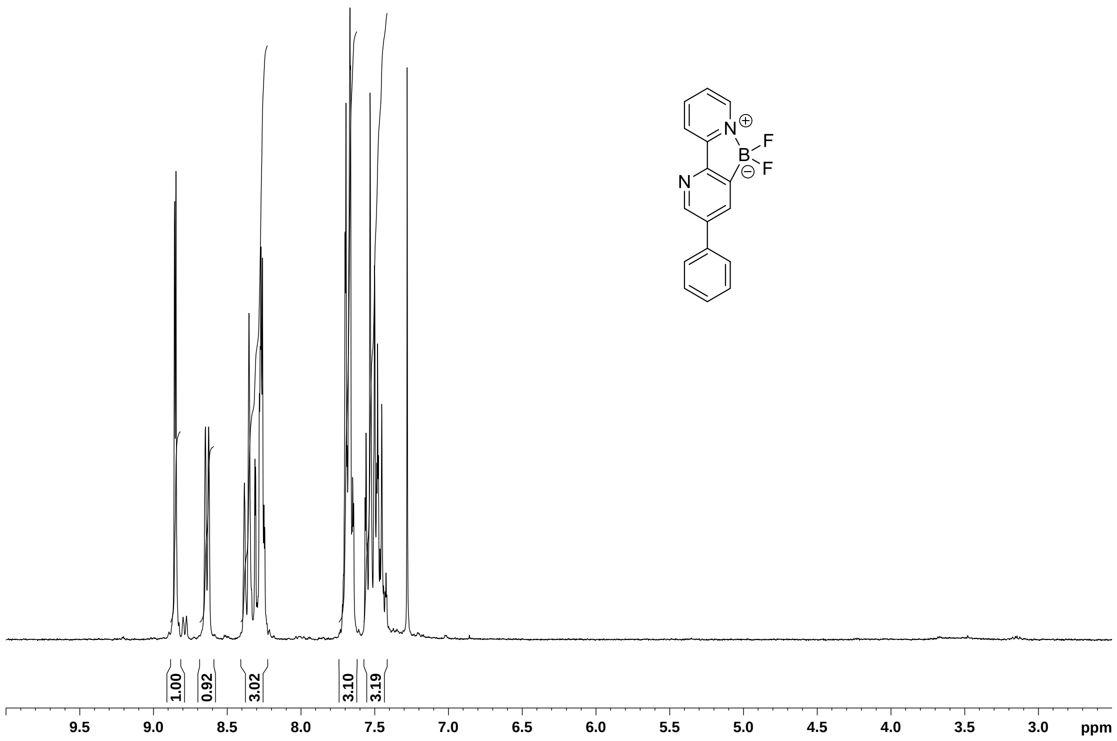
N
B
F
F
N
1.00
0.92
3.02
3.10
3.19
9.5 9.0 8.5 8.0 7.5 7.0 6.5 6.0 5.5 5.0 4.5 4.0 3.5 3.0 ppm

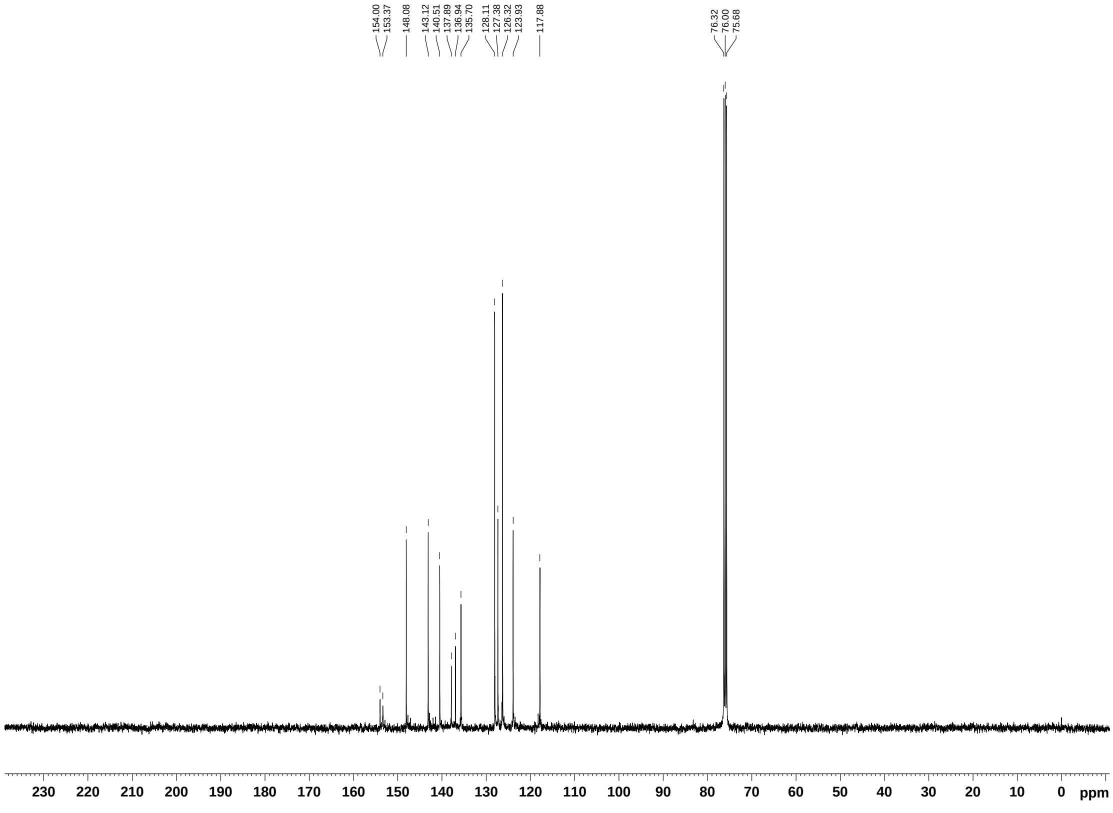
154.00
153.37
148.08
143.12
140.51
137.89
136.94
135.70
128.11
127.38
126.32
123.93
117.88
76.32
76.00
75.68
230 220 210 200 190 180 170 160 150 140 130 120 110 100 90 80 70 60 50 40 30 20 10 0 ppm

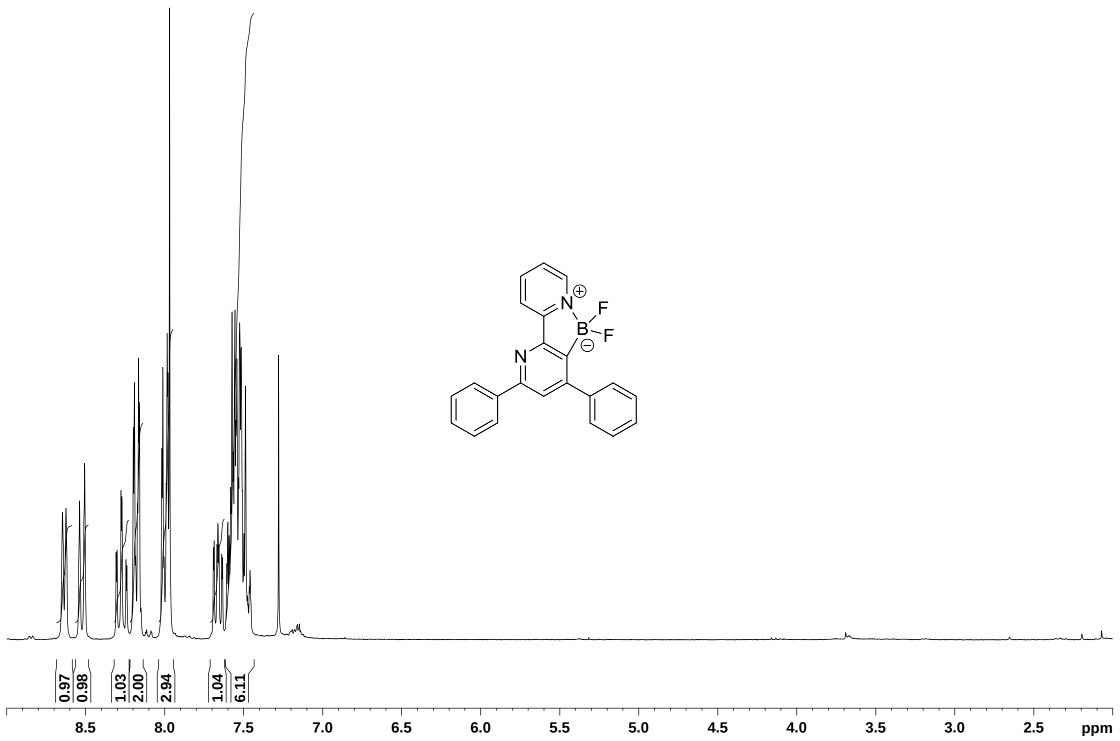
N
N
B
F
F
0.97
0.98
1.03
2.00
2.94
1.04
6.11
8.5 8.0 7.5 7.0 6.5 6.0 5.5 5.0 4.5 4.0 3.5 3.0 2.5 ppm

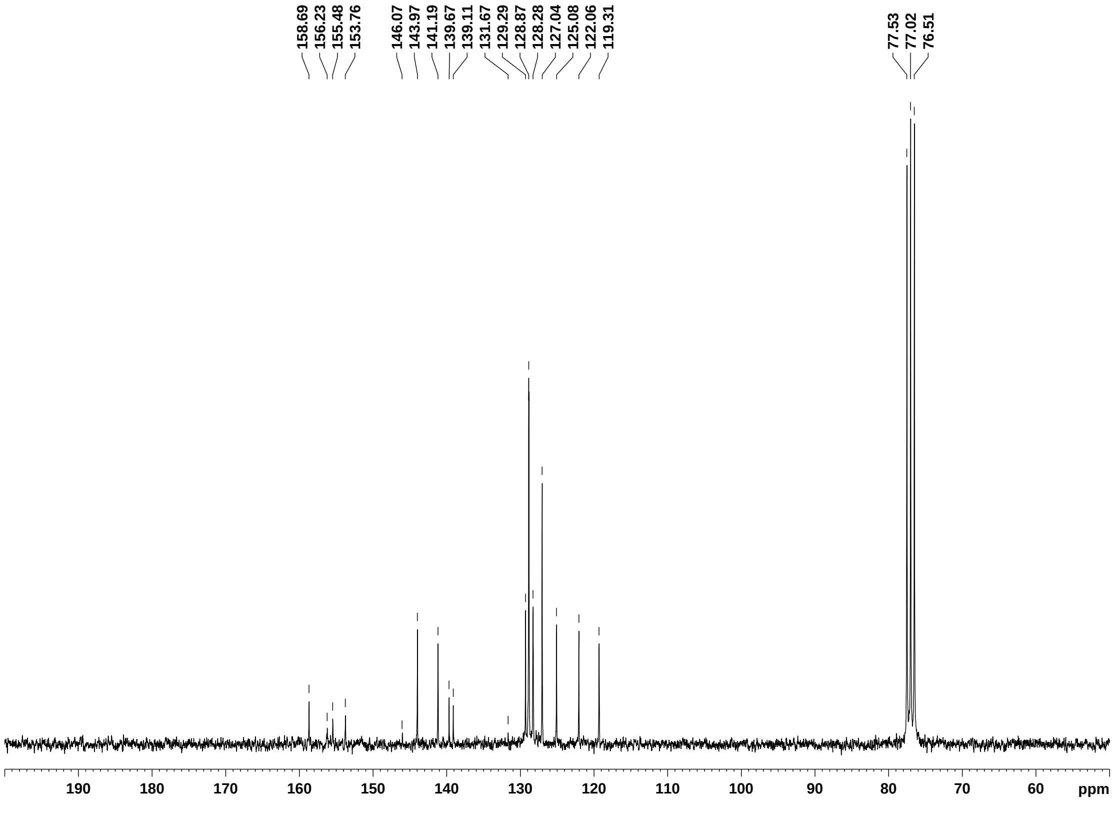
158.69
156.23
155.48
153.76
146.07
143.97
141.19
139.67
139.11
131.67
129.29
128.87
128.28
127.04
125.08
122.06
119.31
77.53
77.02
76.51
190 180 170 160 150 140 130 120 110 100 90 80 70 60 ppm

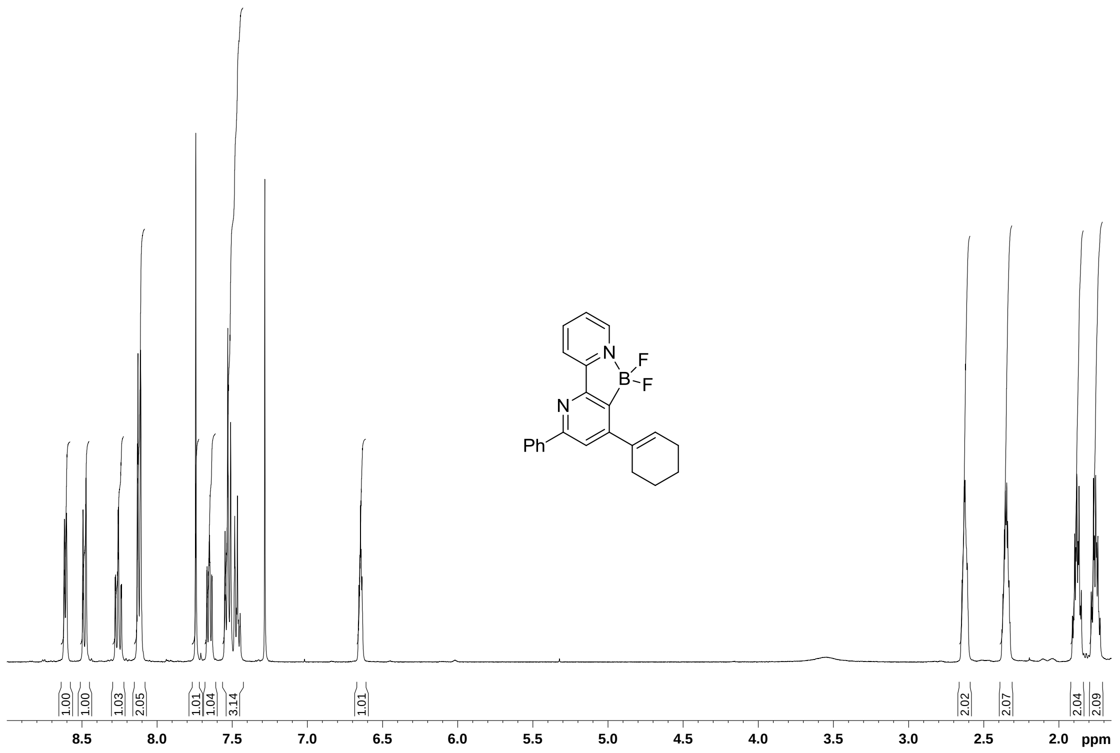
N
B
F
F
N
Ph
1.00
1.00
1.03
2.05
1.01
1.04
3.14
1.01
2.02
2.07
2.04
2.09
8.5
8.0
7.5
7.0
6.5
6.0
5.5
5.0
4.5
4.0
3.5
3.0
2.5
2.0
ppm

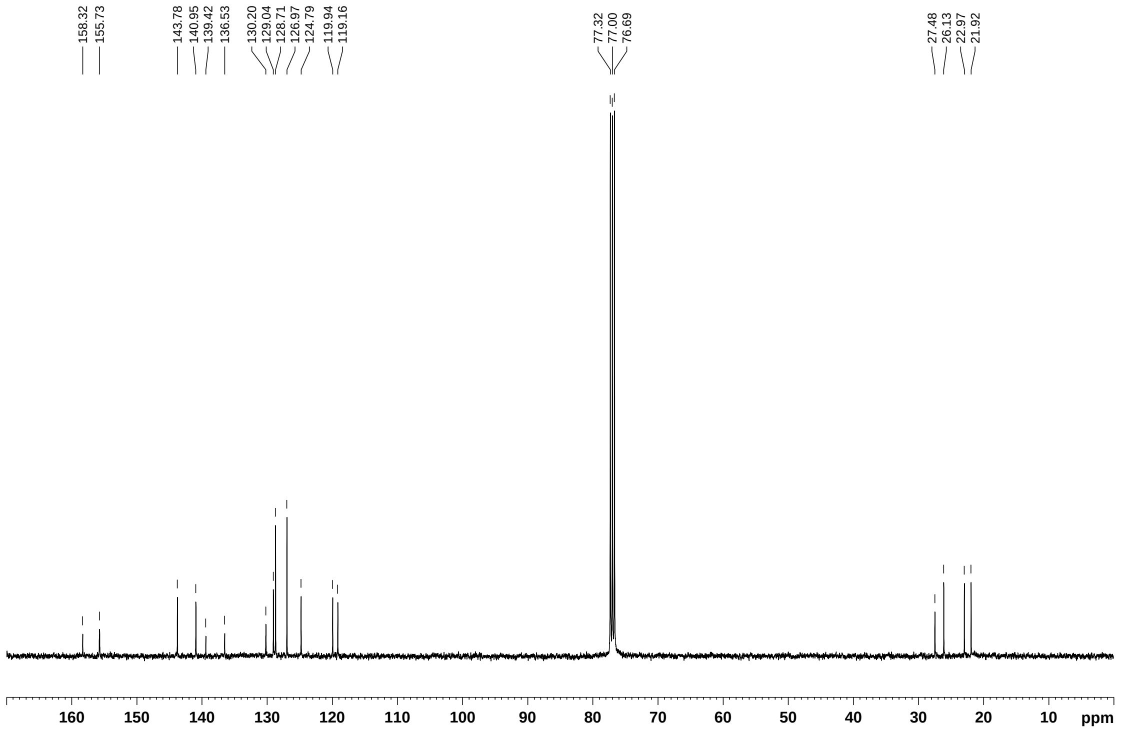
158.32
155.73
143.78
140.95
139.42
136.53
130.20
129.04
128.71
126.97
124.79
119.94
119.16
77.32
77.00
76.69
27.48
26.13
22.97
21.92
160
150
140
130
120
110
100
90
80
70
60
50
40
30
20
10
ppm

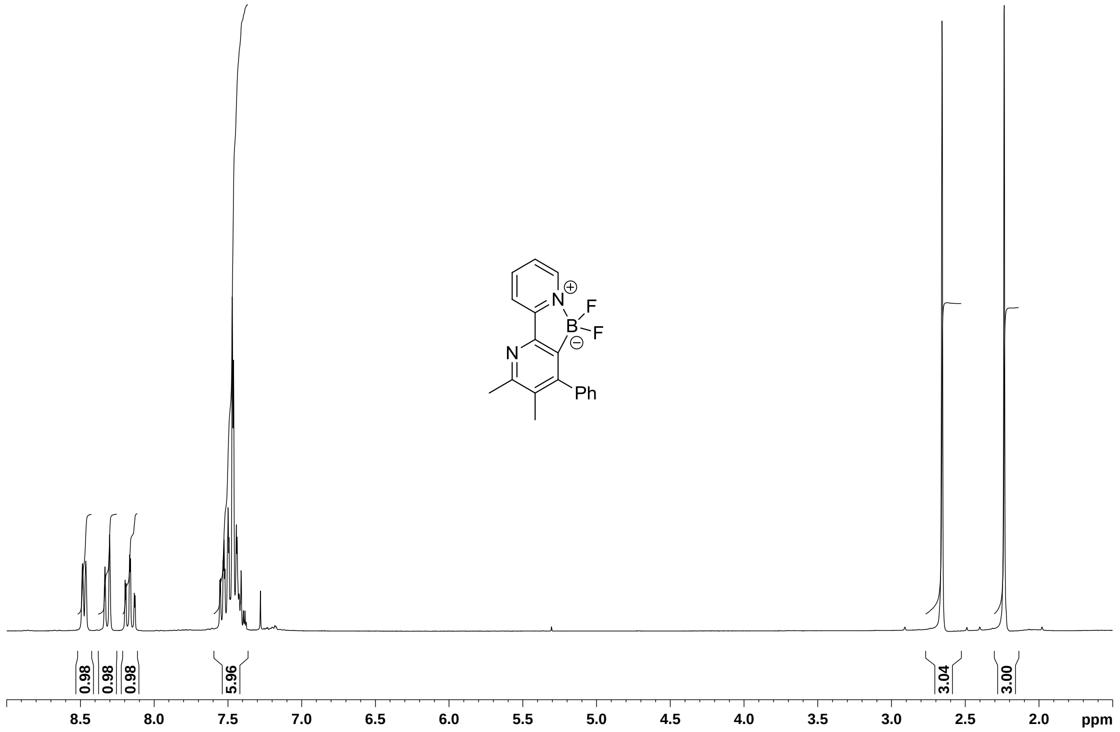
N
F
B
F
N
Ph
0.98
0.98
0.98
5.96
3.04
3.00
8.5 8.0 7.5 7.0 6.5 6.0 5.5 5.0 4.5 4.0 3.5 3.0 2.5 2.0 ppm

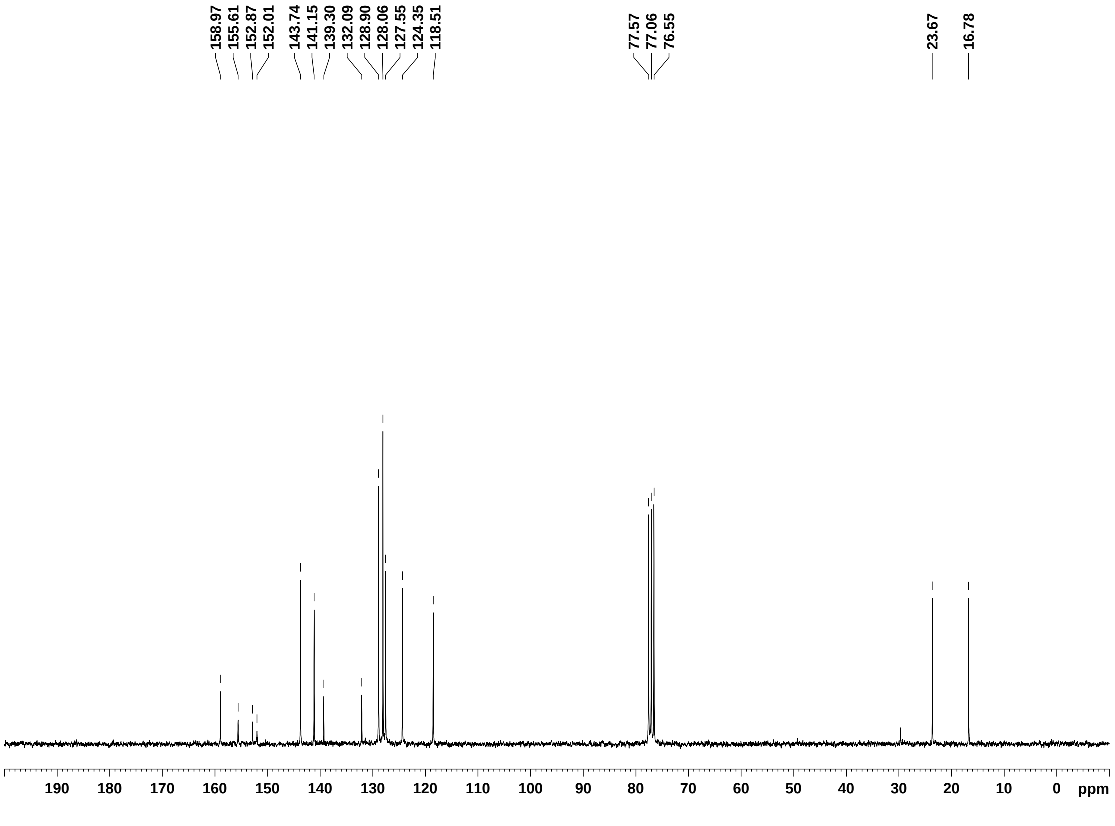
158.97
155.61
152.87
152.01
143.74
141.15
139.30
132.09
128.90
128.06
127.55
124.35
118.51
77.57
77.06
76.55
23.67
16.78
190 180 170 160 150 140 130 120 110 100 90 80 70 60 50 40 30 20 10 0 ppm

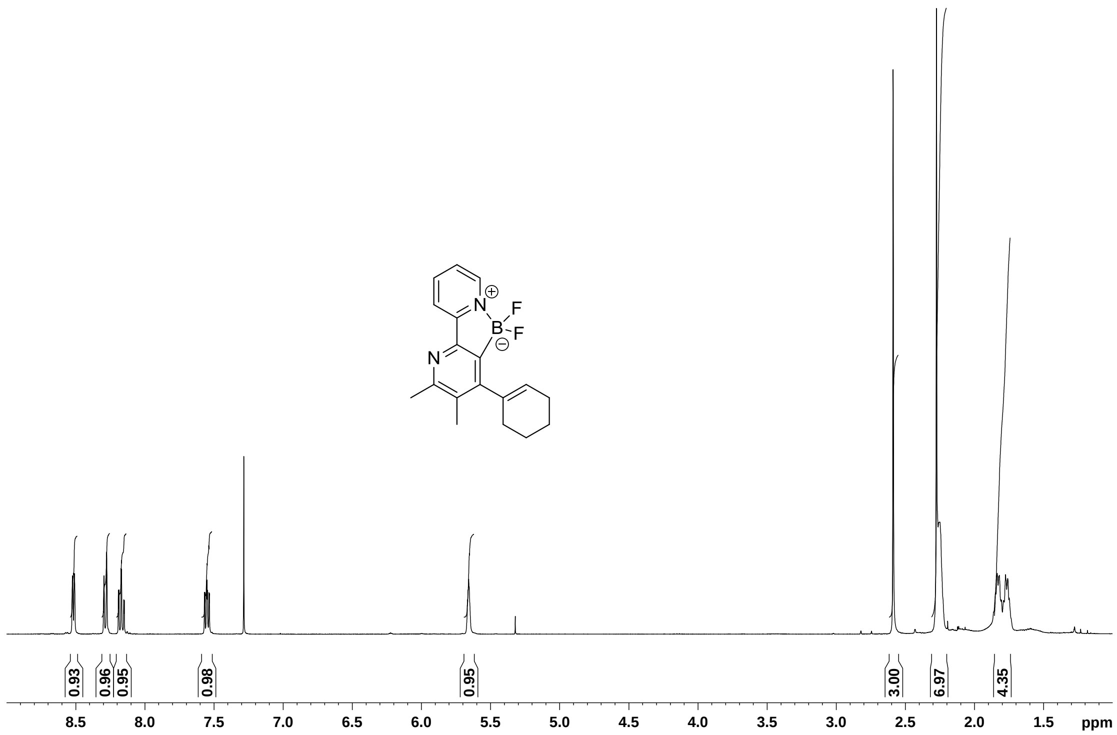
N
B
F
F
N
0.93
0.96
0.95
0.98
0.95
3.00
6.97
4.35
8.5 8.0 7.5 7.0 6.5 6.0 5.5 5.0 4.5 4.0 3.5 3.0 2.5 2.0 1.5 ppm

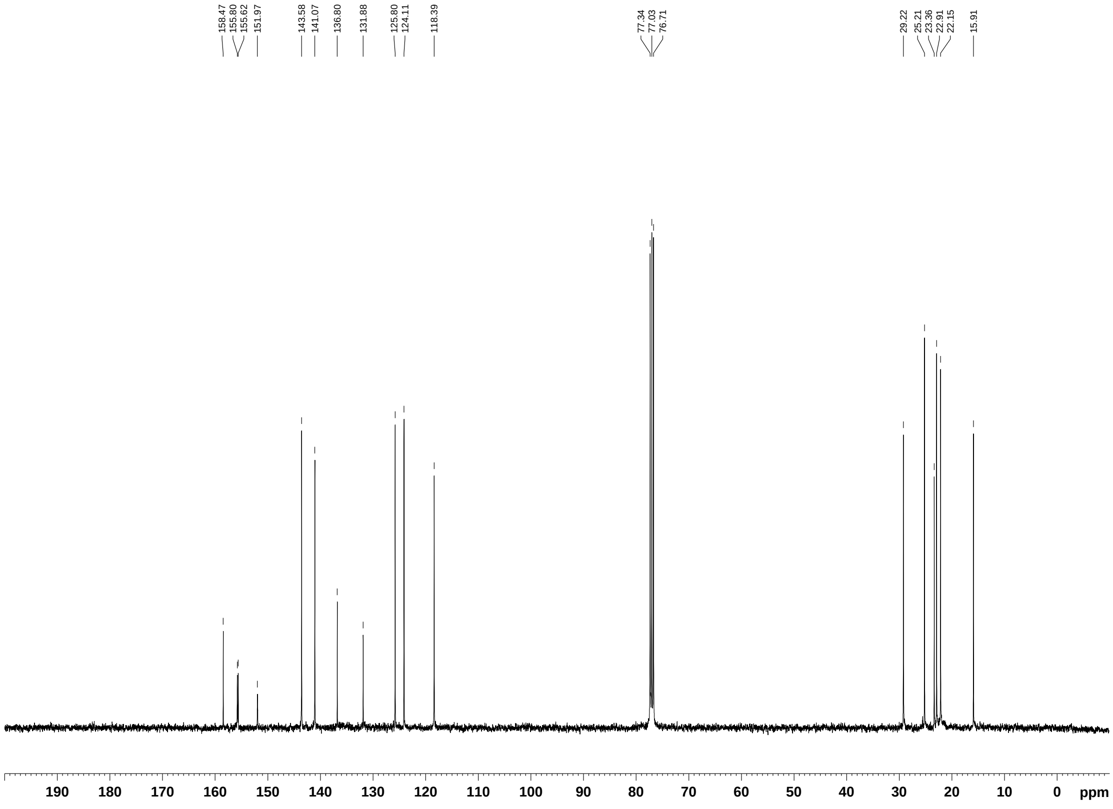
158.47
155.80
155.62
151.97
143.58
141.07
136.80
131.88
125.80
124.11
118.39
77.34
77.03
76.71
29.22
25.21
23.36
22.91
22.15
15.91
190 180 170 160 150 140 130 120 110 100 90 80 70 60 50 40 30 20 10 0 ppm

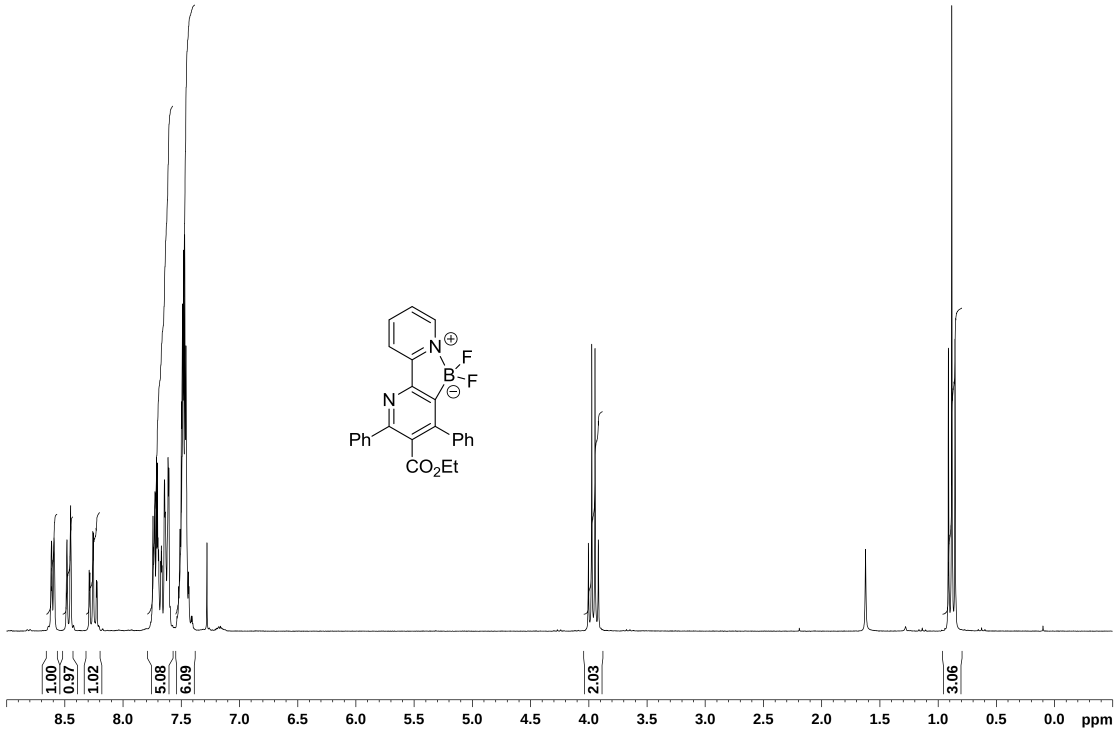
N
B
F
F
N
Ph
Ph
CO_2Et
1.00
0.97
1.02
5.08
6.09
2.03
3.06
8.5 8.0 7.5 7.0 6.5 6.0 5.5 5.0 4.5 4.0 3.5 3.0 2.5 2.0 1.5 1.0 0.5 0.0 ppm

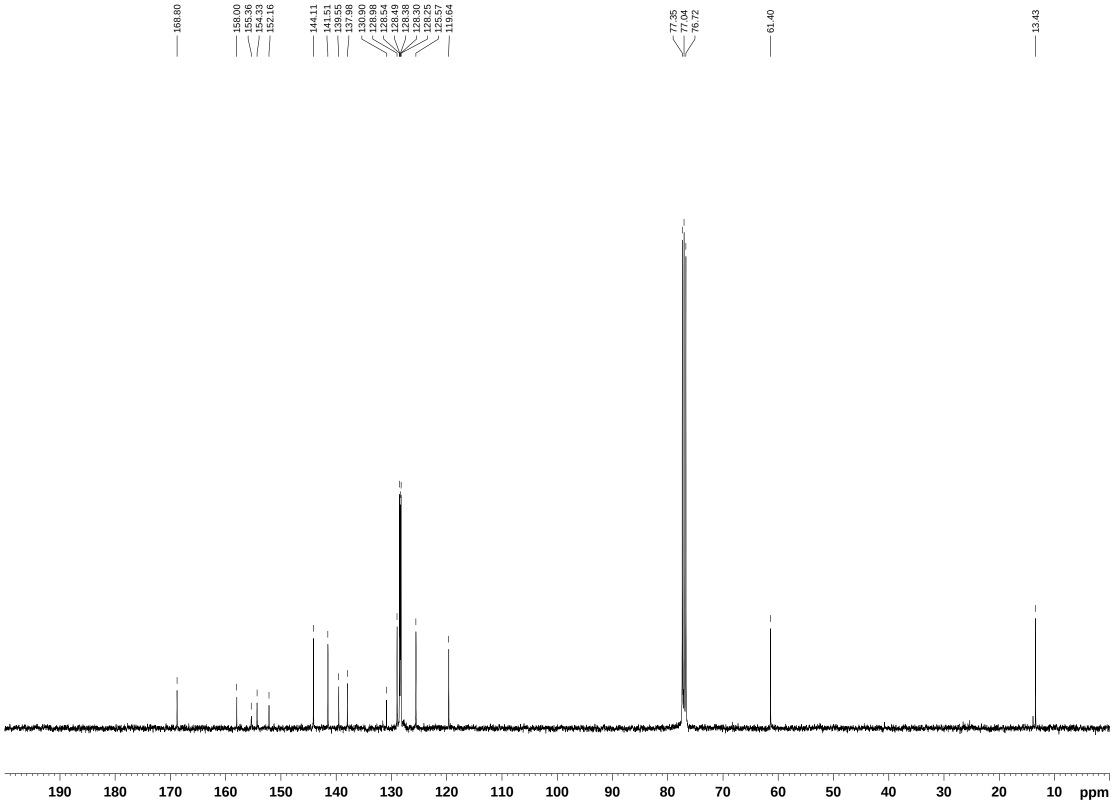
168.80
158.00
155.36
154.33
152.16
144.11
141.51
139.55
137.98
130.90
128.98
128.54
128.49
128.38
128.30
128.25
125.57
119.64
77.35
77.04
76.72
61.40
13.43
190 180 170 160 150 140 130 120 110 100 90 80 70 60 50 40 30 20 10 ppm

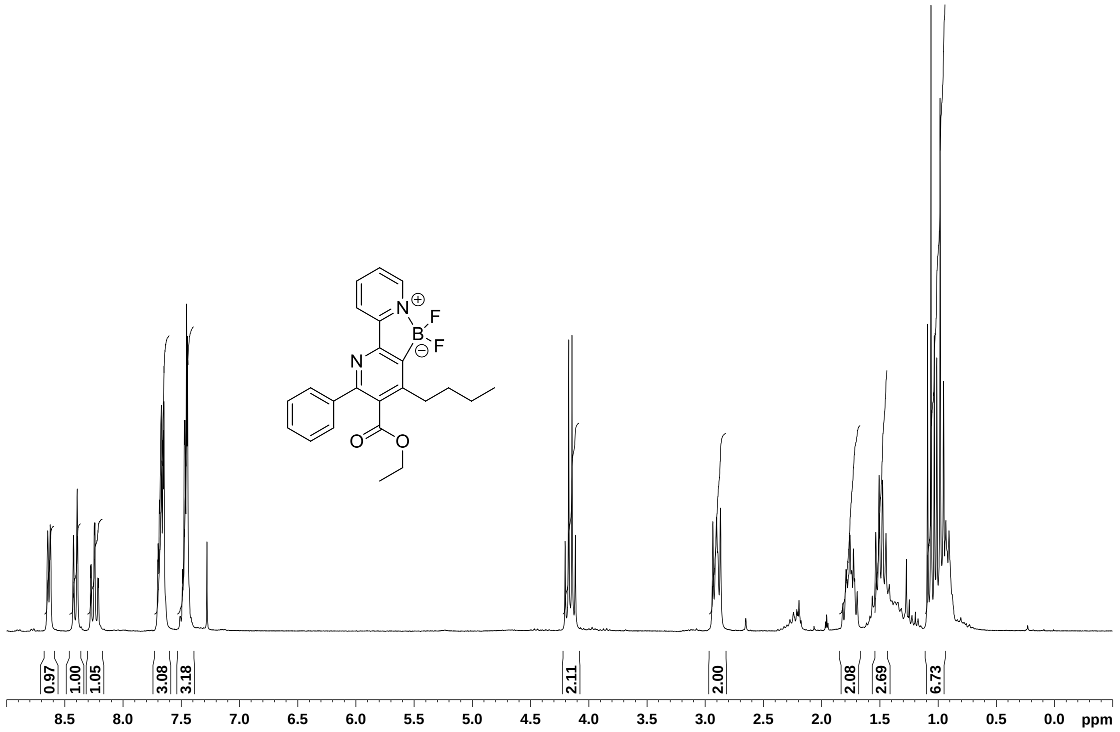
N
N
B
F
F
O
O
0.97
1.00
1.05
3.08
3.18
2.11
2.00
2.08
2.69
6.73
8.5 8.0 7.5 7.0 6.5 6.0 5.5 5.0 4.5 4.0 3.5 3.0 2.5 2.0 1.5 1.0 0.5 0.0 ppm

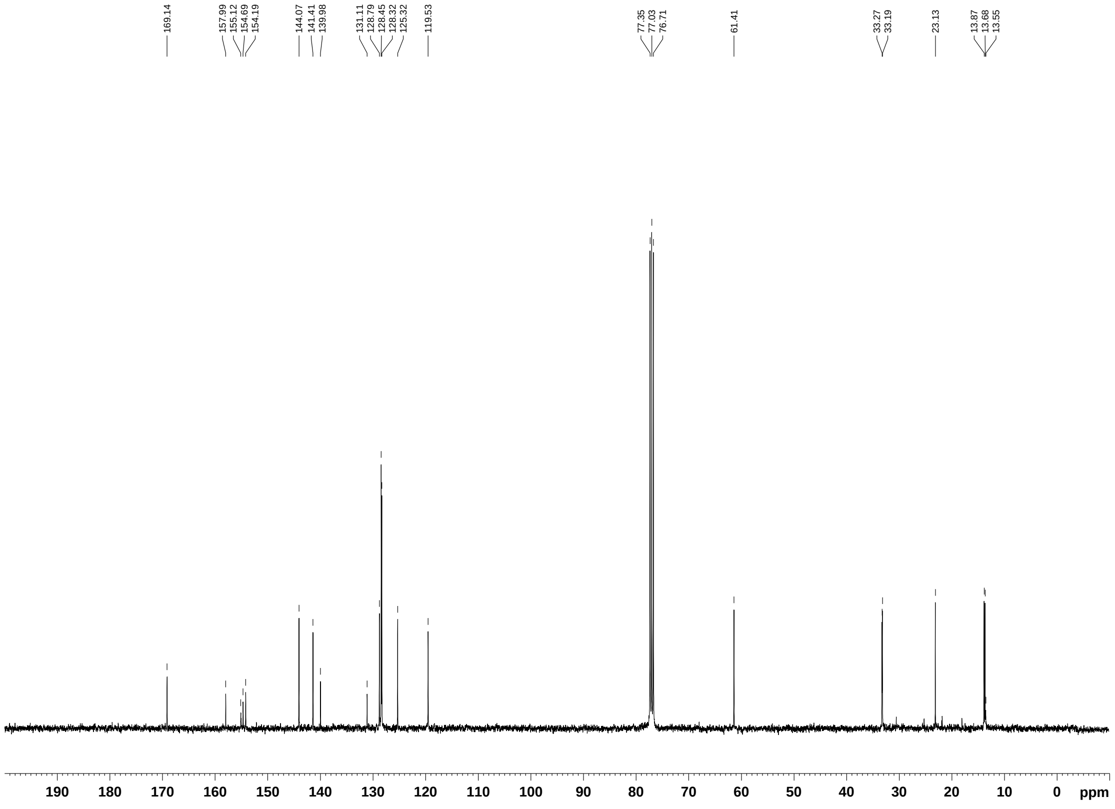
169.14
157.99
155.12
154.69
154.19
144.07
141.41
139.98
131.11
128.79
128.45
128.32
125.32
119.53
77.35
77.03
76.71
61.41
33.27
33.19
23.13
13.87
13.68
13.55
190 180 170 160 150 140 130 120 110 100 90 80 70 60 50 40 30 20 10 0 ppm

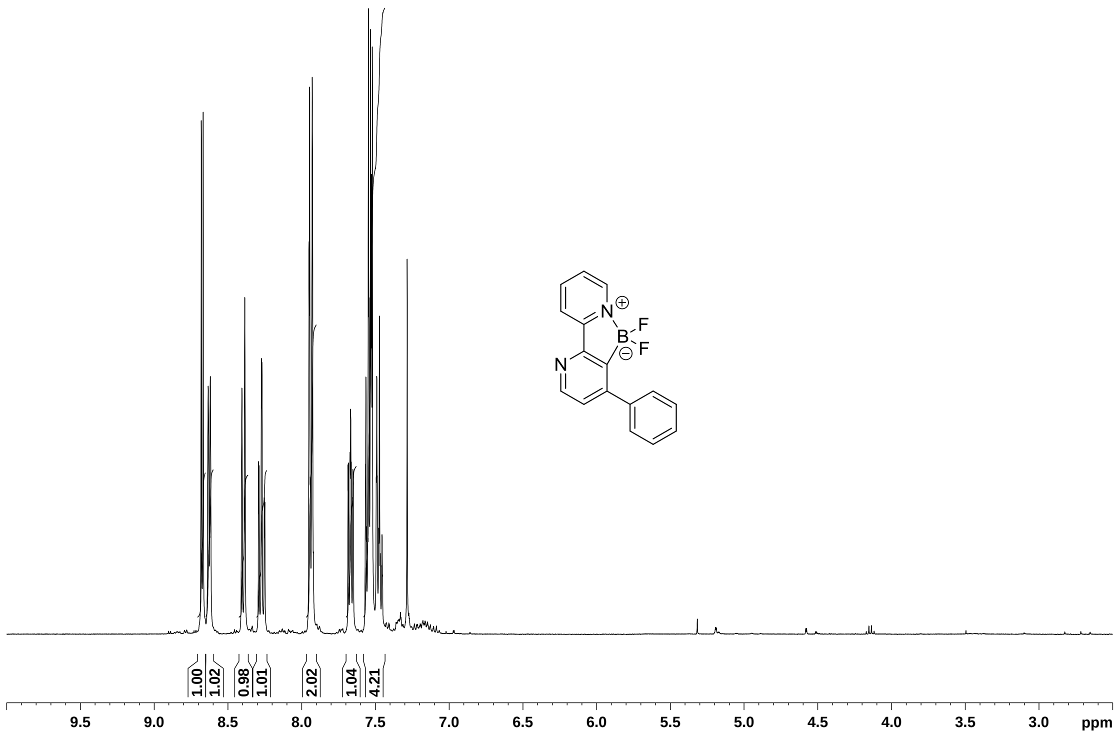
N
B
F
F
N
1.00
1.02
0.98
1.01
2.02
1.04
4.21
9.5 9.0 8.5 8.0 7.5 7.0 6.5 6.0 5.5 5.0 4.5 4.0 3.5 3.0 ppm

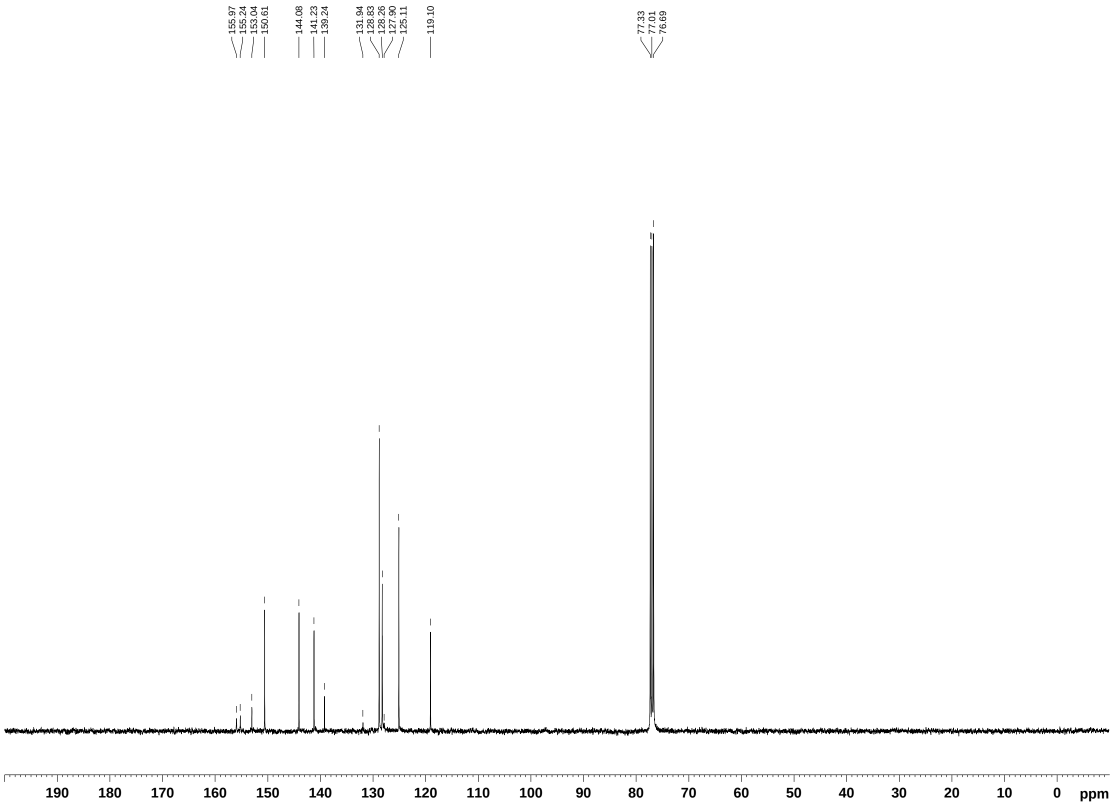
155.97
155.24
153.04
150.61
144.08
141.23
139.24
131.94
128.83
128.26
127.90
125.11
119.10
77.33
77.01
76.69
190 180 170 160 150 140 130 120 110 100 90 80 70 60 50 40 30 20 10 0 ppm

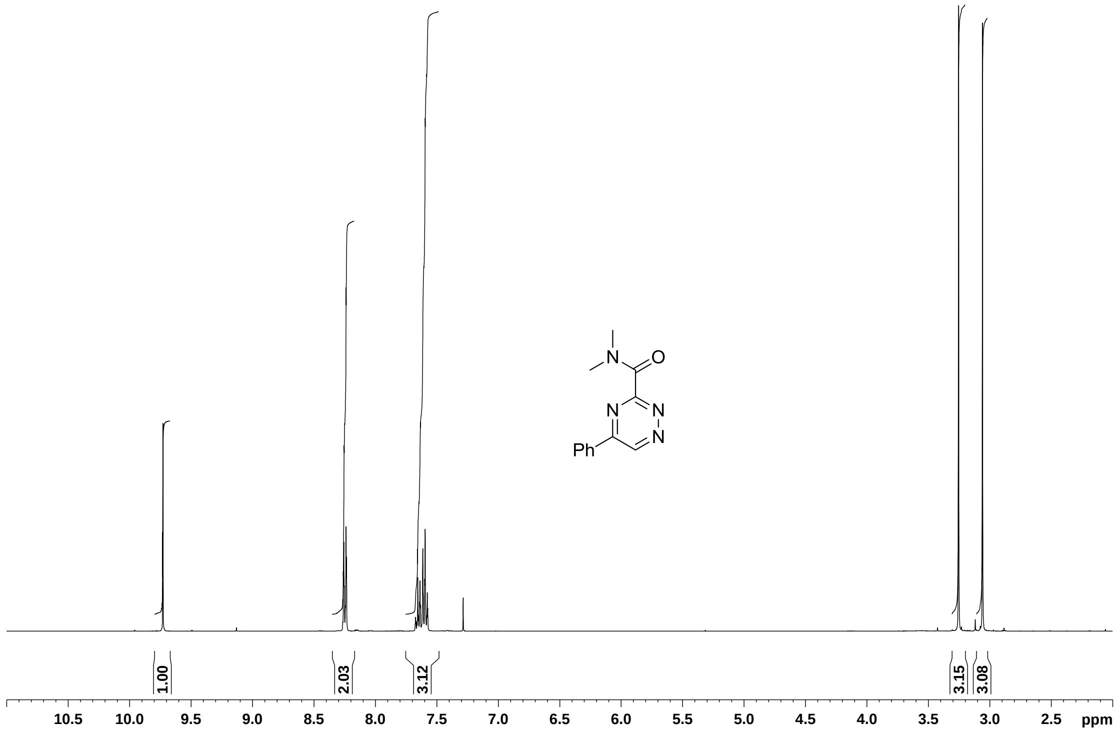
Ph
N
O
1.00
2.03
3.12
3.15
3.08
10.5 10.0 9.5 9.0 8.5 8.0 7.5 7.0 6.5 6.0 5.5 5.0 4.5 4.0 3.5 3.0 2.5 ppm

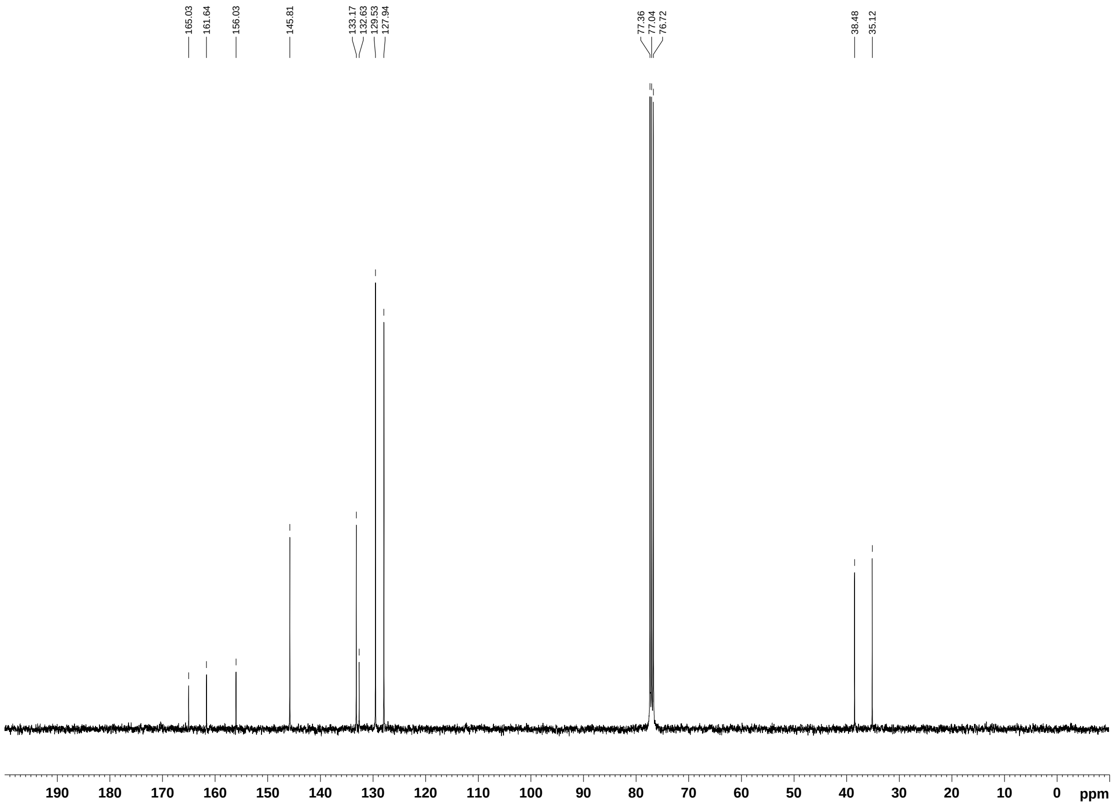
165.03
161.64
156.03
145.81
133.17
132.63
129.53
127.94
77.36
77.04
76.72
38.48
35.12
190 180 170 160 150 140 130 120 110 100 90 80 70 60 50 40 30 20 10 0 ppm

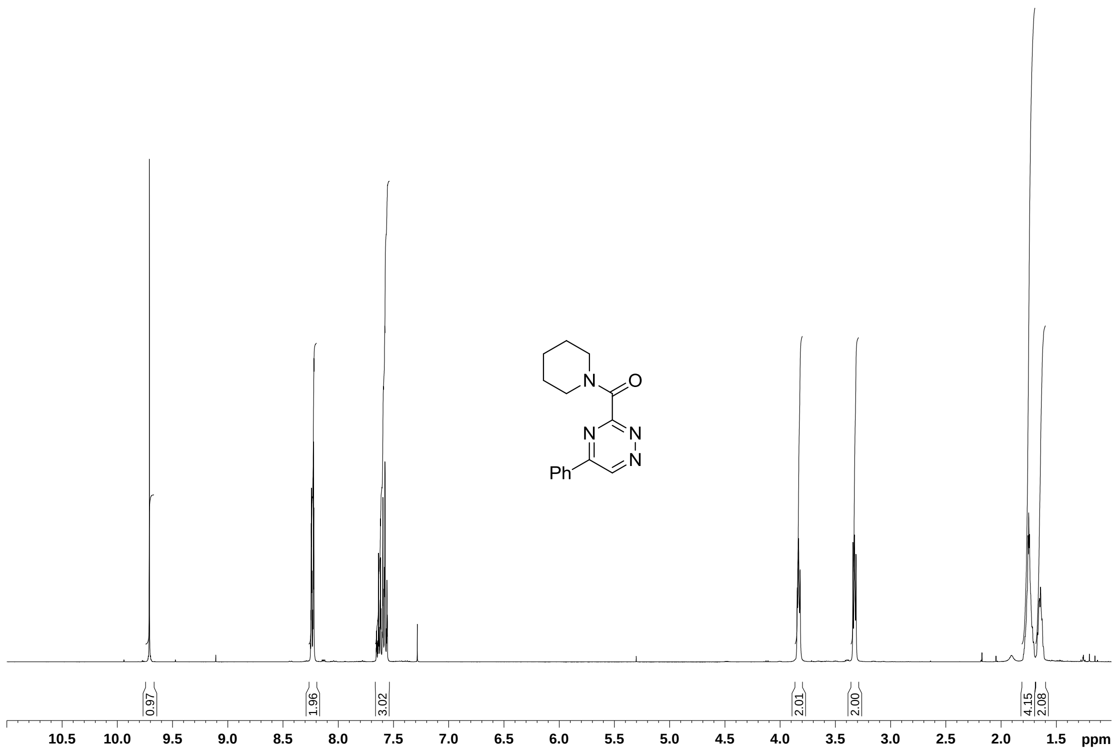

N
O
N
N
N
Ph
0.97
1.96
3.02
2.01
2.00
4.15
2.08
10.5
10.0
9.5
9.0
8.5
8.0
7.5
7.0
6.5
6.0
5.5
5.0
4.5
4.0
3.5
3.0
2.5
2.0
1.5
ppm

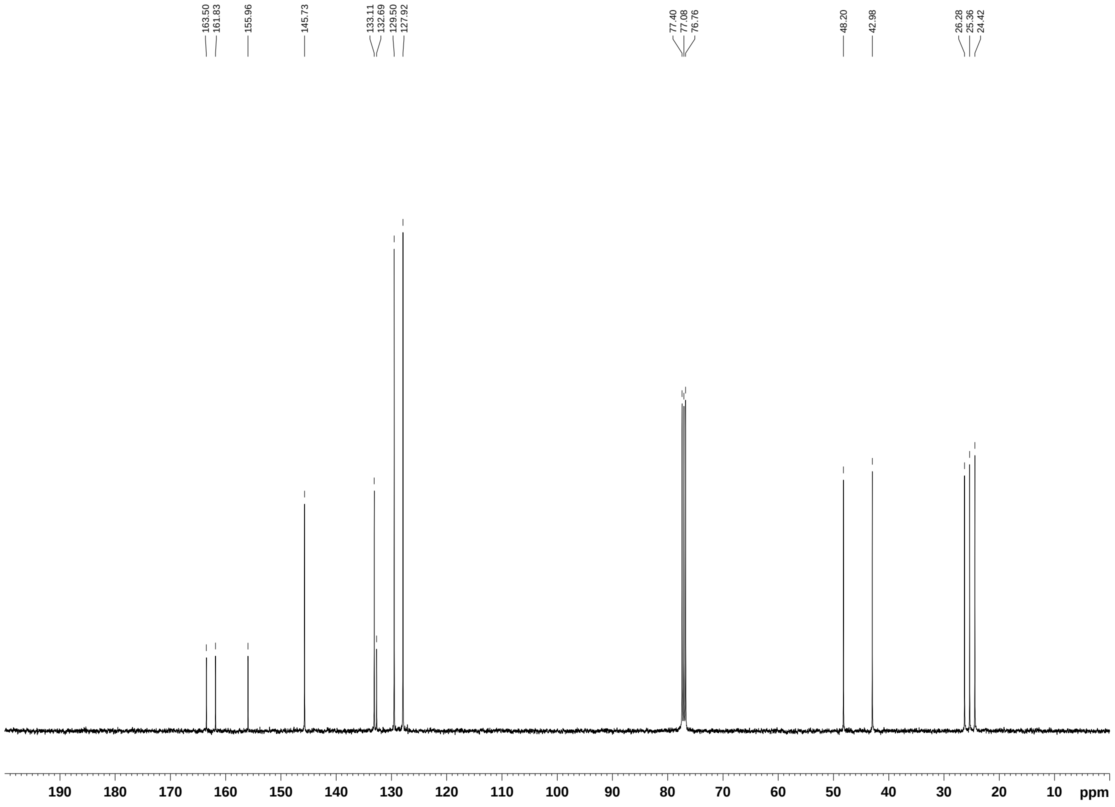

163.50
161.83
155.96
145.73
133.11
132.69
129.50
127.92
77.40
77.08
76.76
48.20
42.98
26.28
25.36
24.42
190
180
170
160
150
140
130
120
110
100
90
80
70
60
50
40
30
20
10
ppm

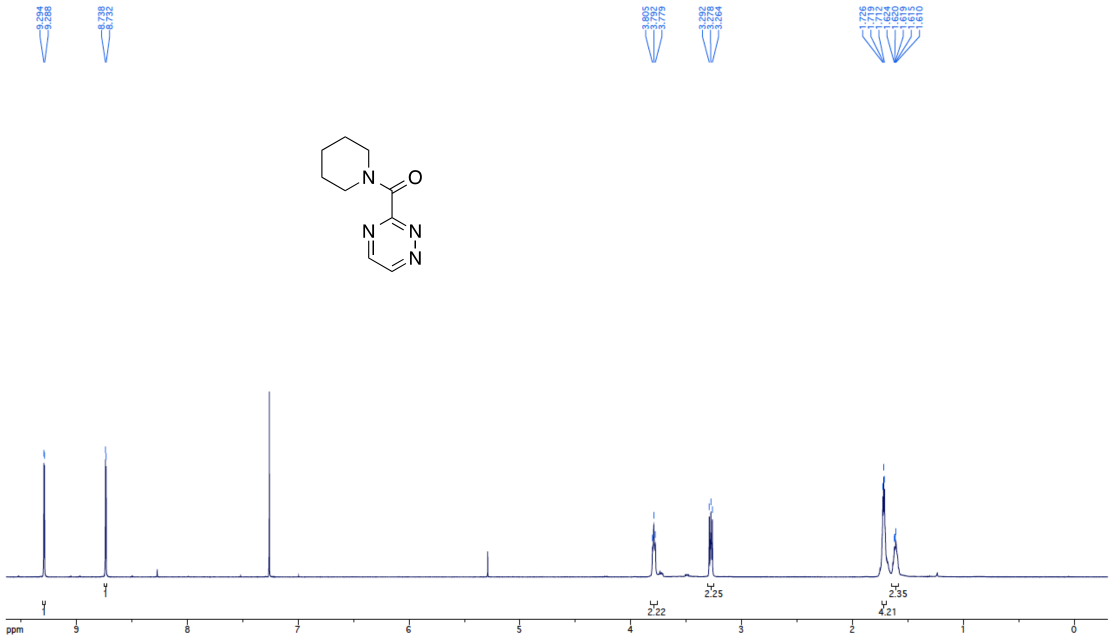

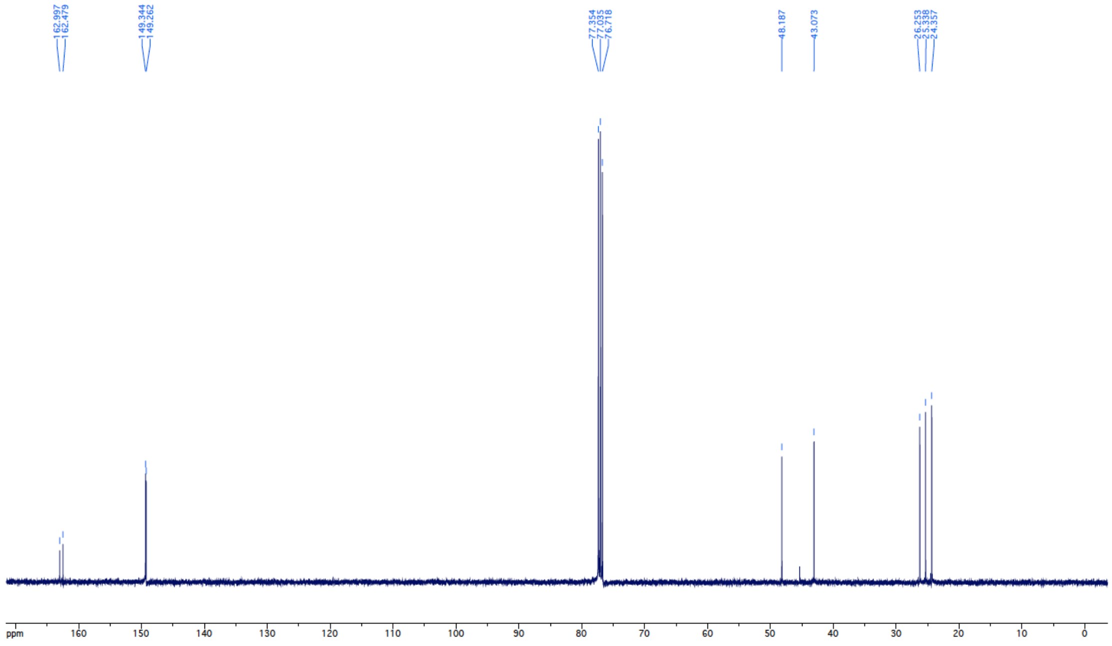

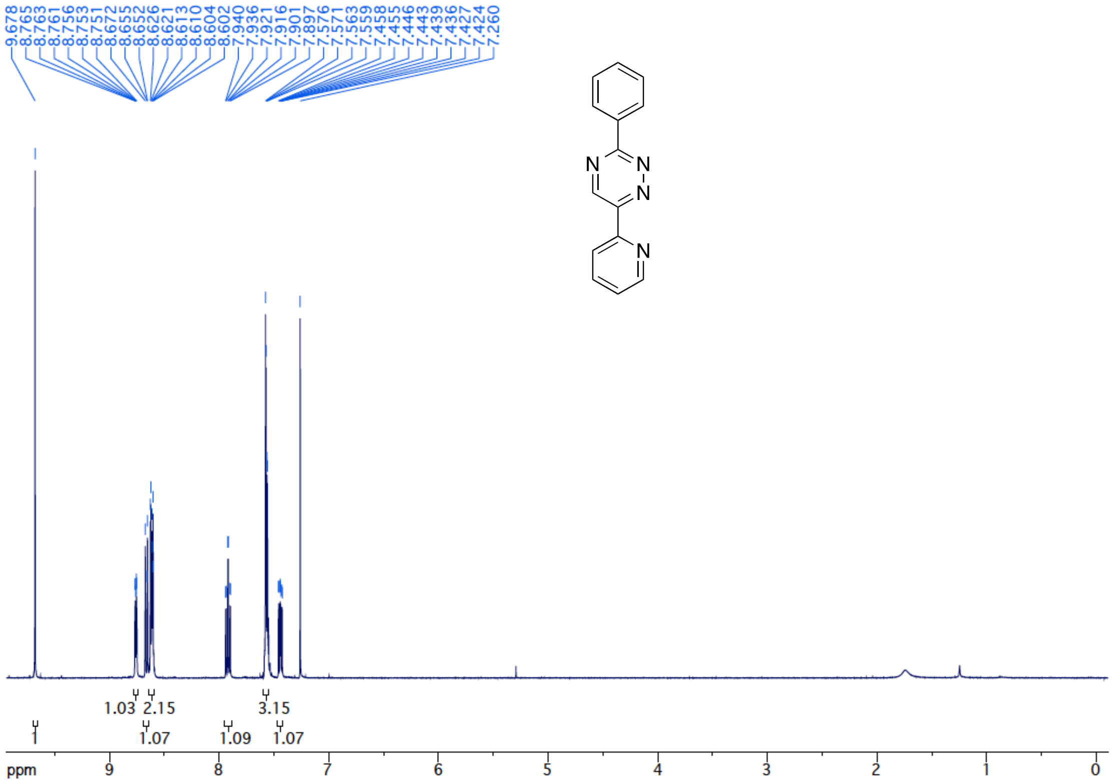
9.678
8.765
8.761
8.756
8.753
8.751
8.672
8.655
8.652
8.626
8.621
8.613
8.610
8.604
8.602
7.940
7.936
7.921
7.916
7.901
7.897
7.576
7.571
7.563
7.559
7.458
7.455
7.446
7.443
7.439
7.436
7.427
7.424
7.260
N
N
N
N
1
1.03
2.15
1.07
1.09
3.15
1.07
ppm
9
8
7
6
5
4
3
2
1
0

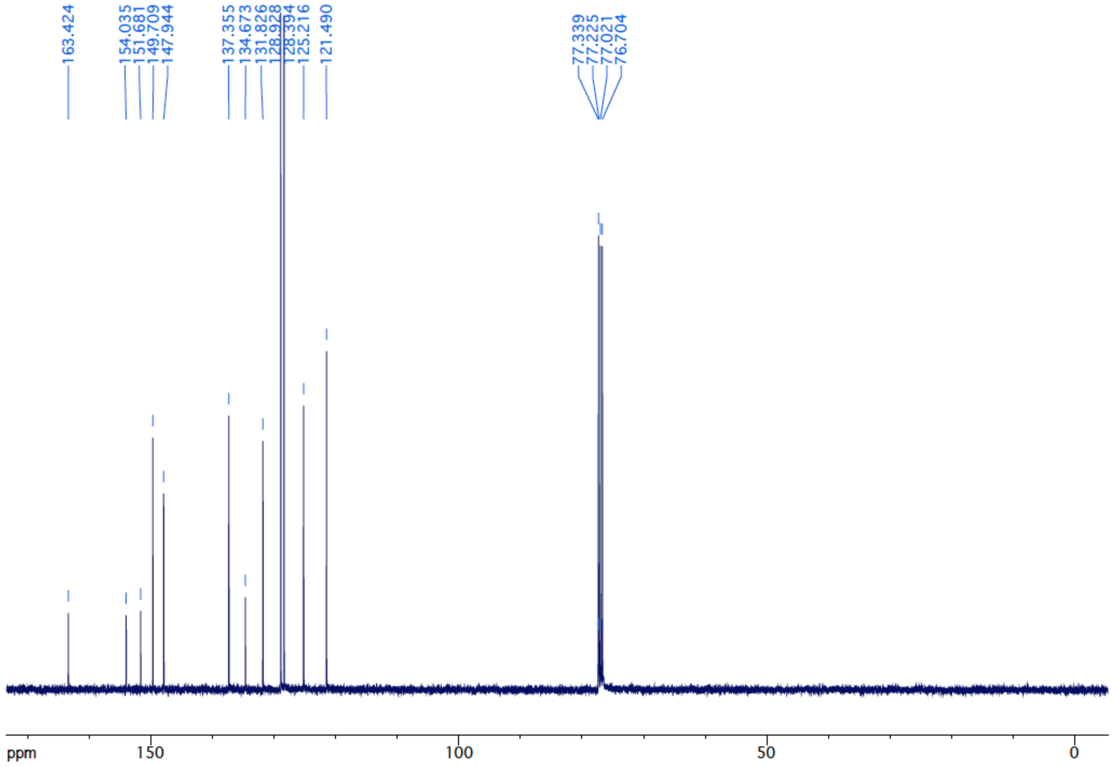
163.424
154.035
151.681
149.709
147.944
137.355
134.673
131.826
128.928
128.394
125.216
121.490
77.339
77.225
77.021
76.704
ppm
150
100
50
0

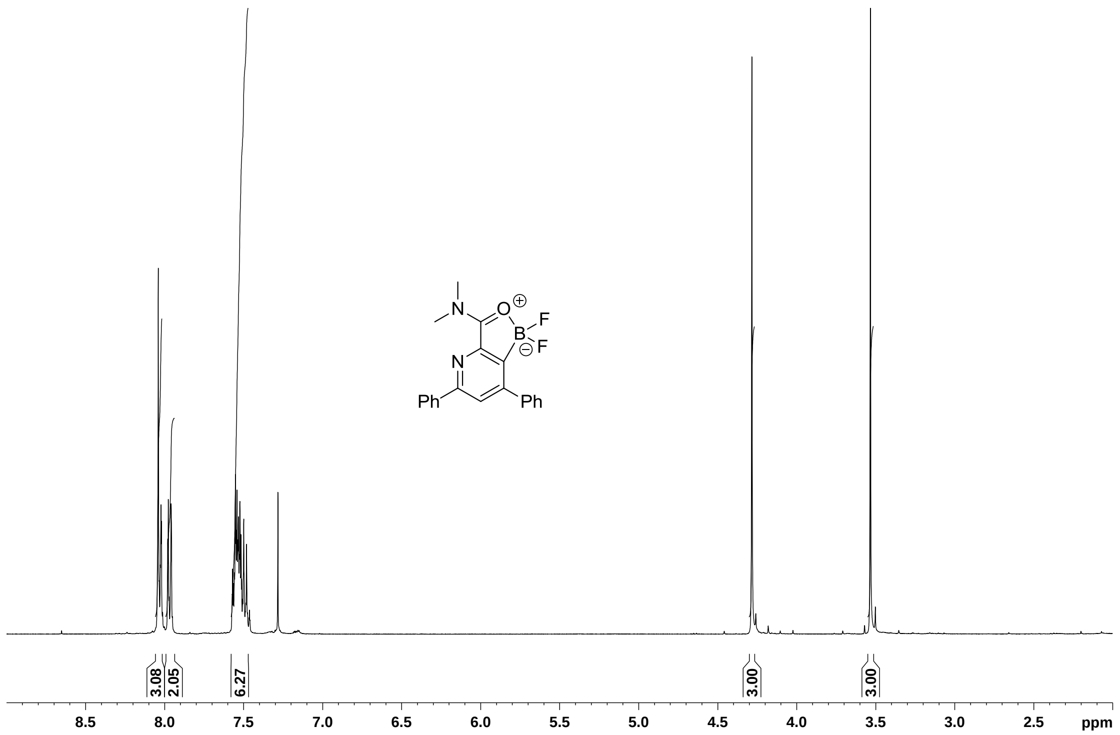
N
O
B
F
F
N
Ph
Ph
3.08
2.05
6.27
3.00
3.00
8.5 8.0 7.5 7.0 6.5 6.0 5.5 5.0 4.5 4.0 3.5 3.0 2.5 ppm

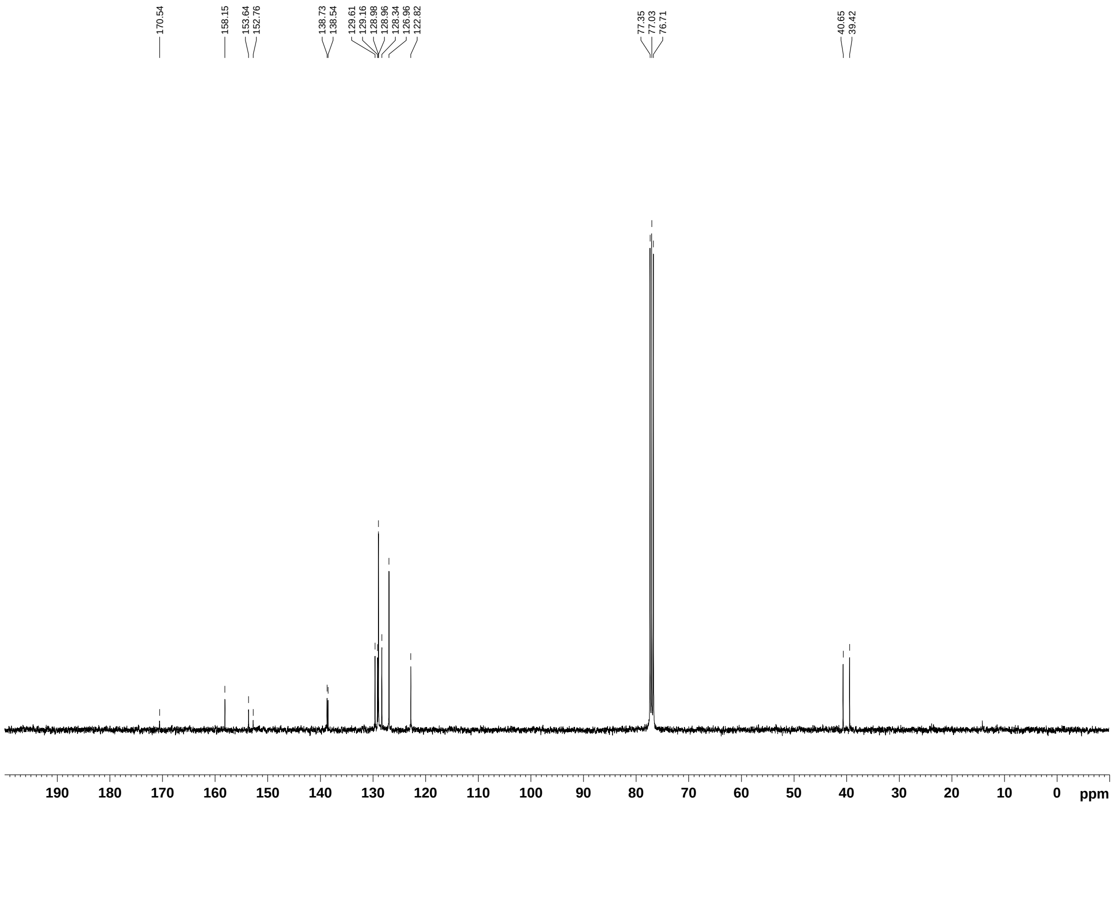
170.54
158.15
153.64
152.76
138.73
138.54
129.61
129.16
128.98
128.96
128.34
126.96
122.82
77.35
77.03
76.71
40.65
39.42
190 180 170 160 150 140 130 120 110 100 90 80 70 60 50 40 30 20 10 0 ppm

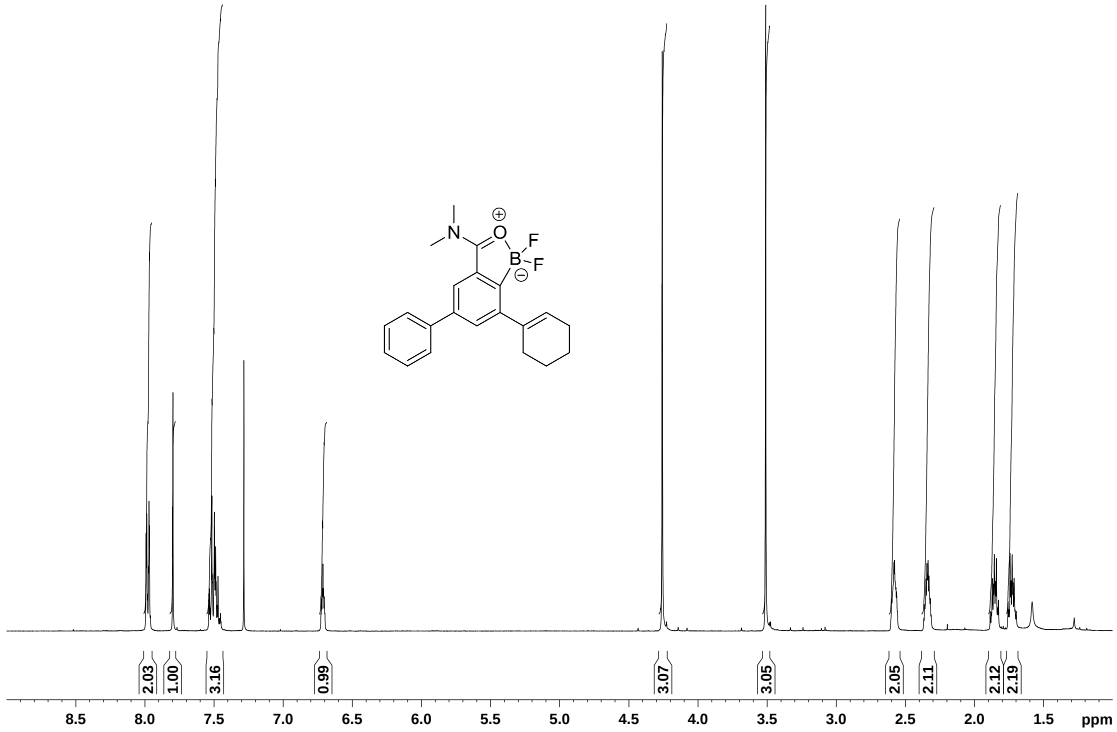

N
O
B
F
F
2.03
1.00
3.16
0.99
3.07
3.05
2.05
2.11
2.12
2.19
8.5 8.0 7.5 7.0 6.5 6.0 5.5 5.0 4.5 4.0 3.5 3.0 2.5 2.0 1.5 ppm

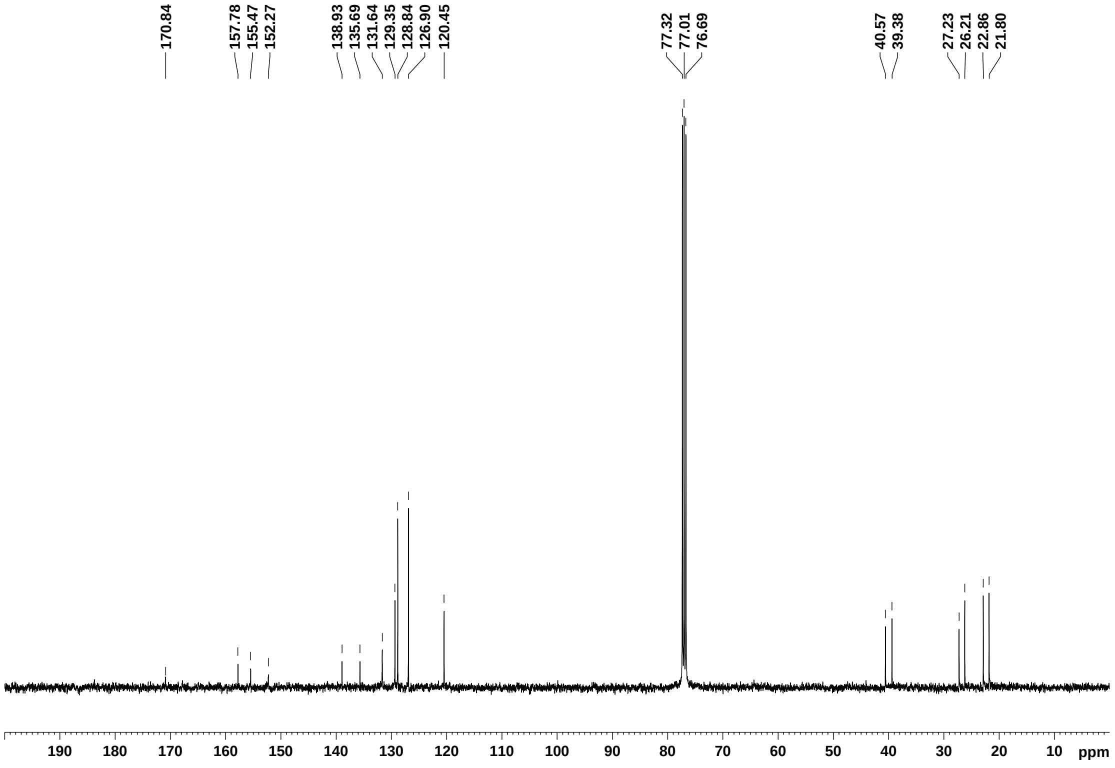

170.84
157.78
155.47
152.27
138.93
135.69
131.64
129.35
128.84
126.90
120.45
77.32
77.01
76.69
40.57
39.38
27.23
26.21
22.86
21.80
190 180 170 160 150 140 130 120 110 100 90 80 70 60 50 40 30 20 10 ppm

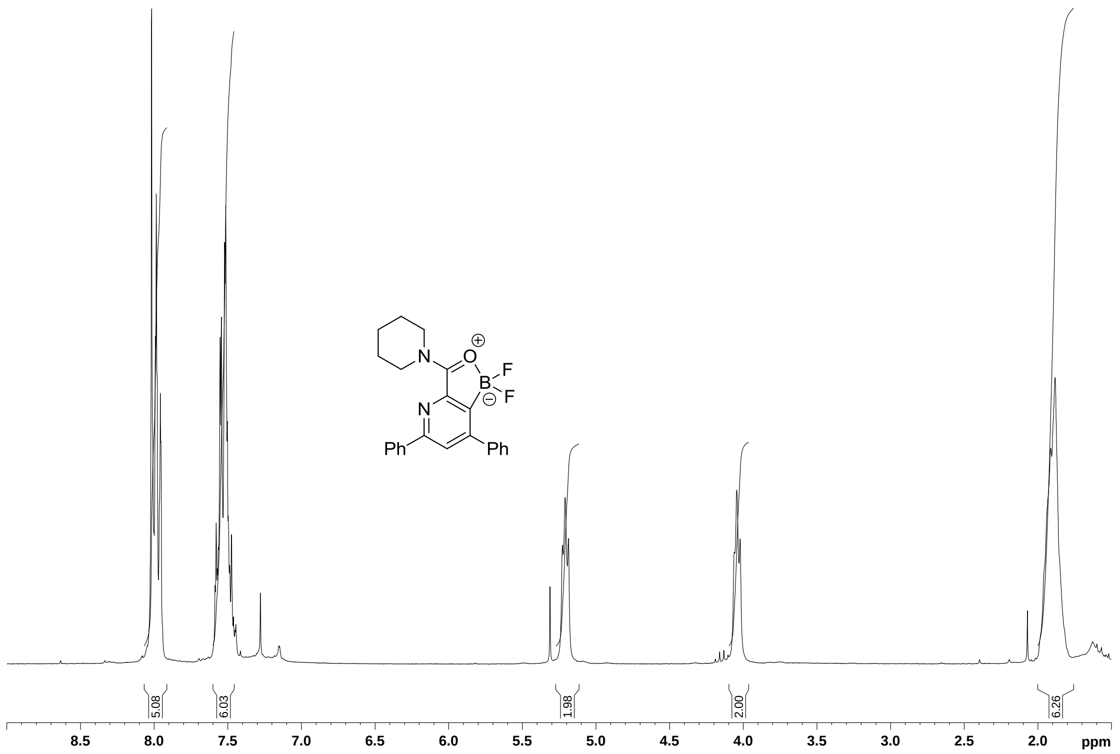
N
O
B
F
F
N
Ph
Ph
5.08
6.03
1.98
2.00
6.26
8.5 8.0 7.5 7.0 6.5 6.0 5.5 5.0 4.5 4.0 3.5 3.0 2.5 2.0 ppm

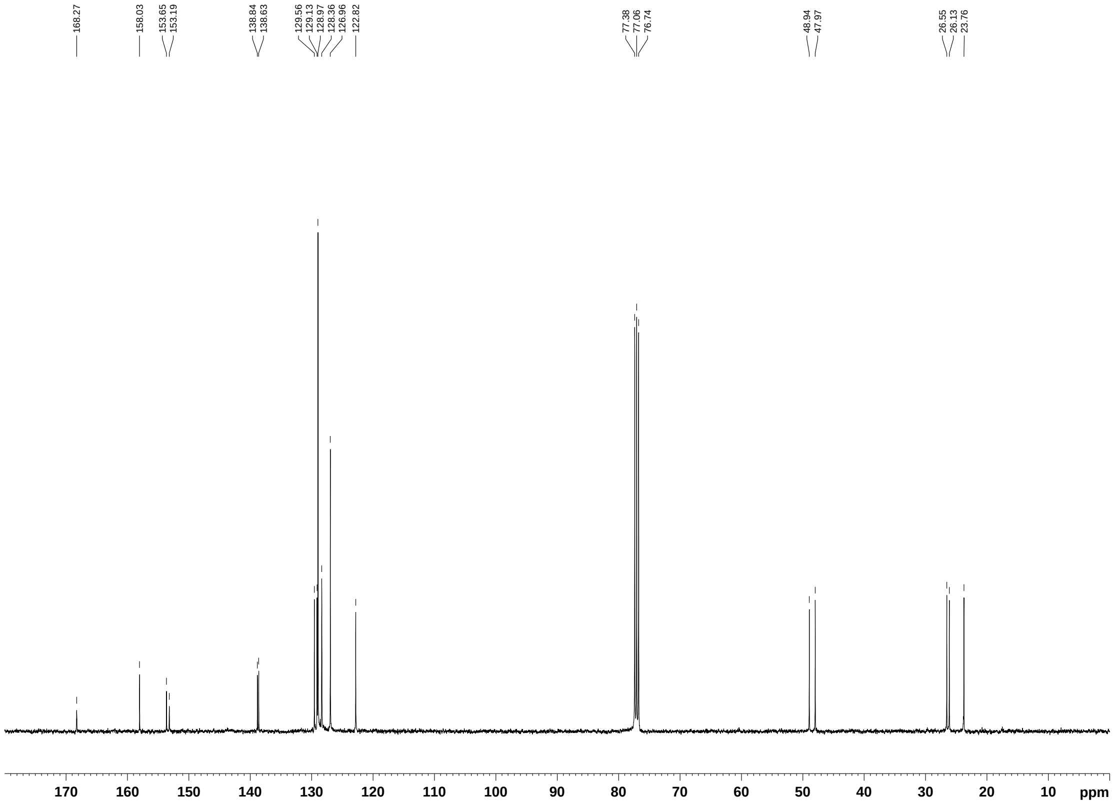
168.27
158.03
153.65
153.19
138.84
138.63
129.56
129.13
128.97
128.36
126.96
122.82
77.38
77.06
76.74
48.94
47.97
26.55
26.13
23.76
170 160 150 140 130 120 110 100 90 80 70 60 50 40 30 20 10 ppm

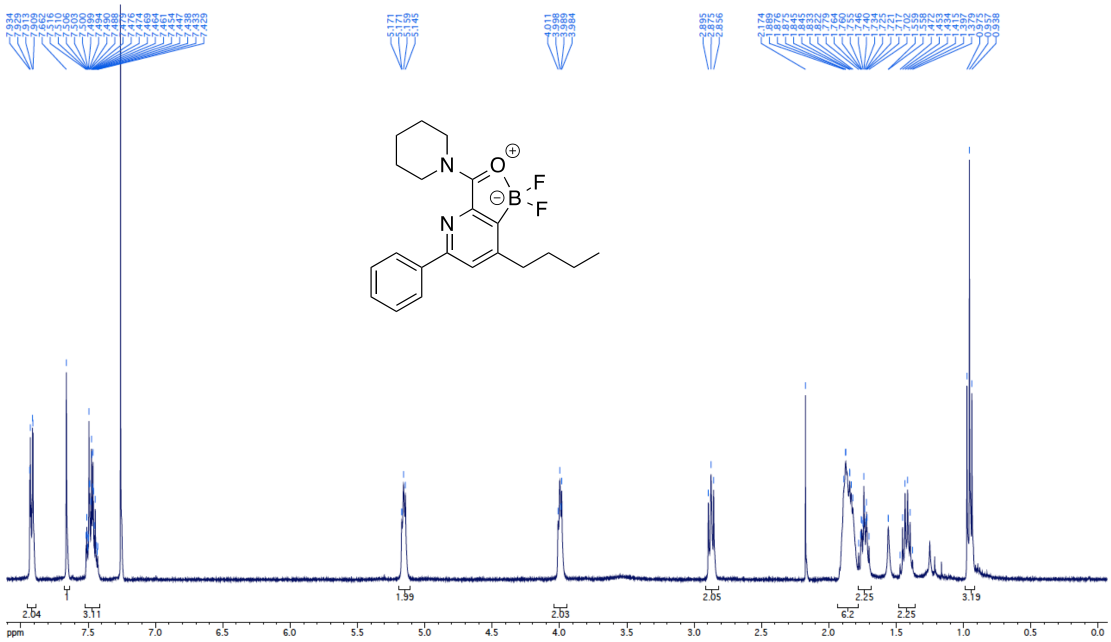
7.934
7.929
7.913
7.909
7.662
7.516
7.510
7.506
7.503
7.500
7.499
7.494
7.490
7.488
7.479
7.476
7.474
7.469
7.464
7.461
7.454
7.447
7.438
7.433
7.429
5.171
5.171
5.159
5.145
4.011
3.998
3.989
3.984
2.895
2.875
2.856
2.174
1.889
1.876
1.875
1.845
1.845
1.833
1.822
1.779
1.764
1.760
1.755
1.746
1.740
1.734
1.725
1.721
1.717
1.702
1.559
1.558
1.472
1.453
1.434
1.415
1.397
1.379
0.975
0.957
0.938
N
O
B
F
F
N
2.04
3.11
1.99
2.03
2.05
6.2
2.25
2.25
3.19
ppm
7.5
7.0
6.5
6.0
5.5
5.0
4.5
4.0
3.5
3.0
2.5
2.0
1.5
1.0
0.5
0.0

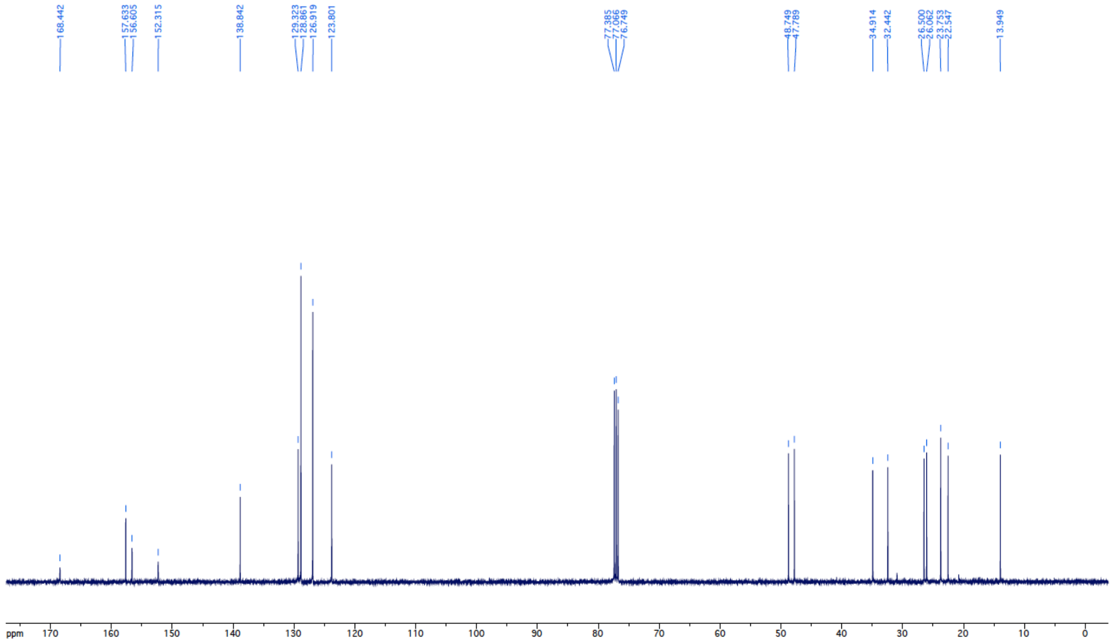
168.442
157.633
156.605
152.315
138.842
129.323
128.861
126.919
123.801
77.385
77.066
76.749
48.749
47.789
34.914
32.442
26.500
26.062
23.753
22.547
13.949
ppm
170
160
150
140
130
120
110
100
90
80
70
60
50
40
30
20
10
0

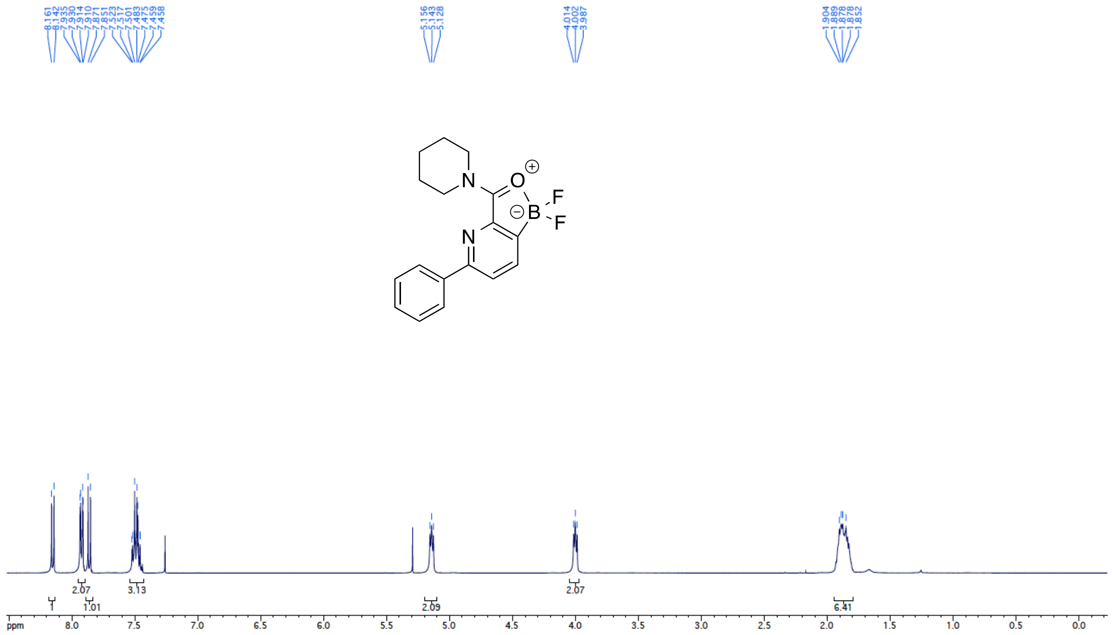
8.161
8.142
7.935
7.930
7.914
7.910
7.871
7.851
7.523
7.517
7.501
7.483
7.475
7.459
7.458
5.156
5.143
5.128
4.014
4.002
3.987
1.904
1.889
1.878
1.878
1.852
N
O
B
F
F
N
2.07
1.01
3.13
2.09
2.07
6.41
ppm
8.0
7.5
7.0
6.5
6.0
5.5
5.0
4.5
4.0
3.5
3.0
2.5
2.0
1.5
1.0
0.5
0.0

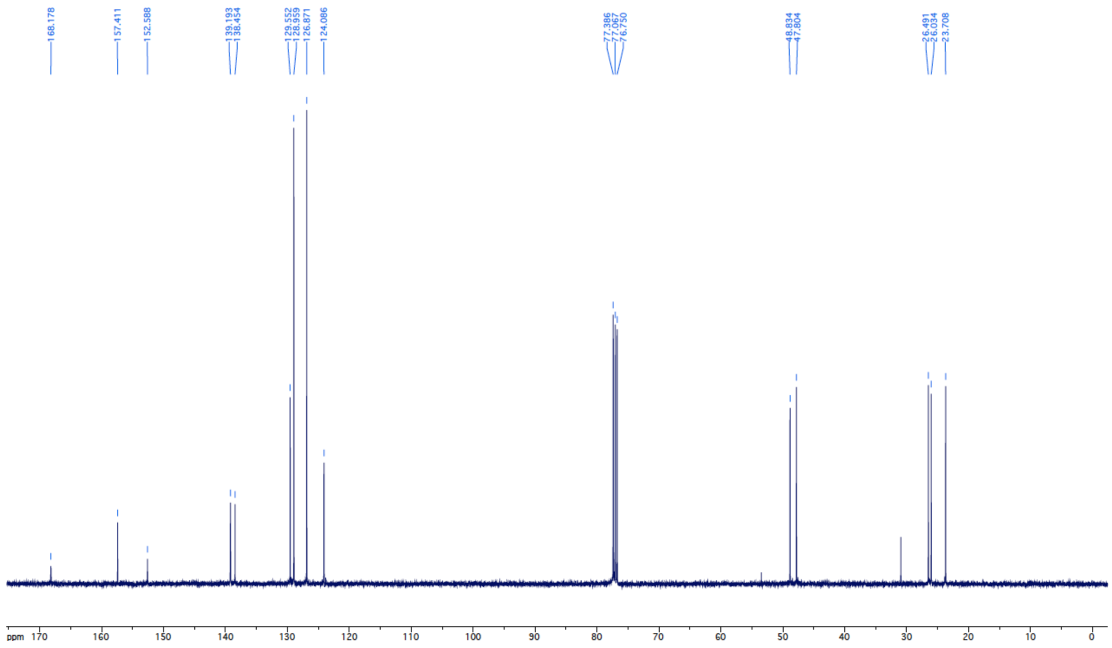
168.178
157.411
152.588
139.193
138.454
129.552
128.959
126.871
124.086
77.386
77.067
76.750
48.834
47.804
26.491
26.034
23.708
ppm
170
160
150
140
130
120
110
100
90
80
70
60
50
40
30
20
10
0

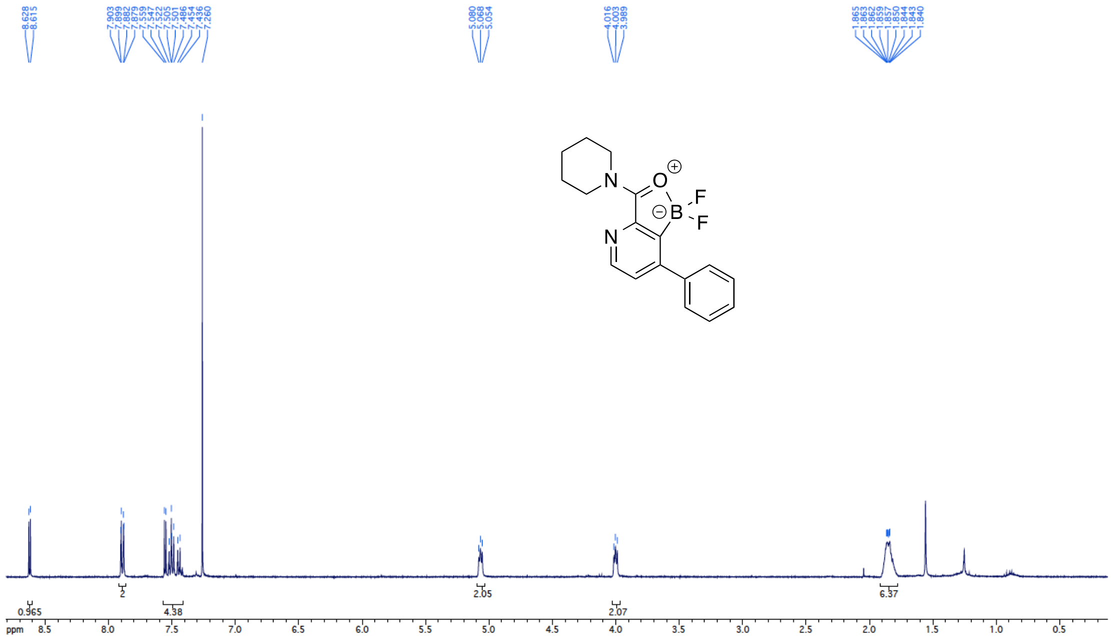
8.628
8.615
7.903
7.899
7.882
7.879
7.559
7.547
7.527
7.505
7.501
7.486
7.454
7.436
7.260
5.080
5.068
5.054
4.016
4.003
3.989
1.865
1.863
1.862
1.859
1.857
1.850
1.844
1.843
1.840
N
O
B
F
F
N
0.965
2
4.38
2.05
2.07
6.37
ppm
8.5
8.0
7.5
7.0
6.5
6.0
5.5
5.0
4.5
4.0
3.5
3.0
2.5
2.0
1.5
1.0
0.5

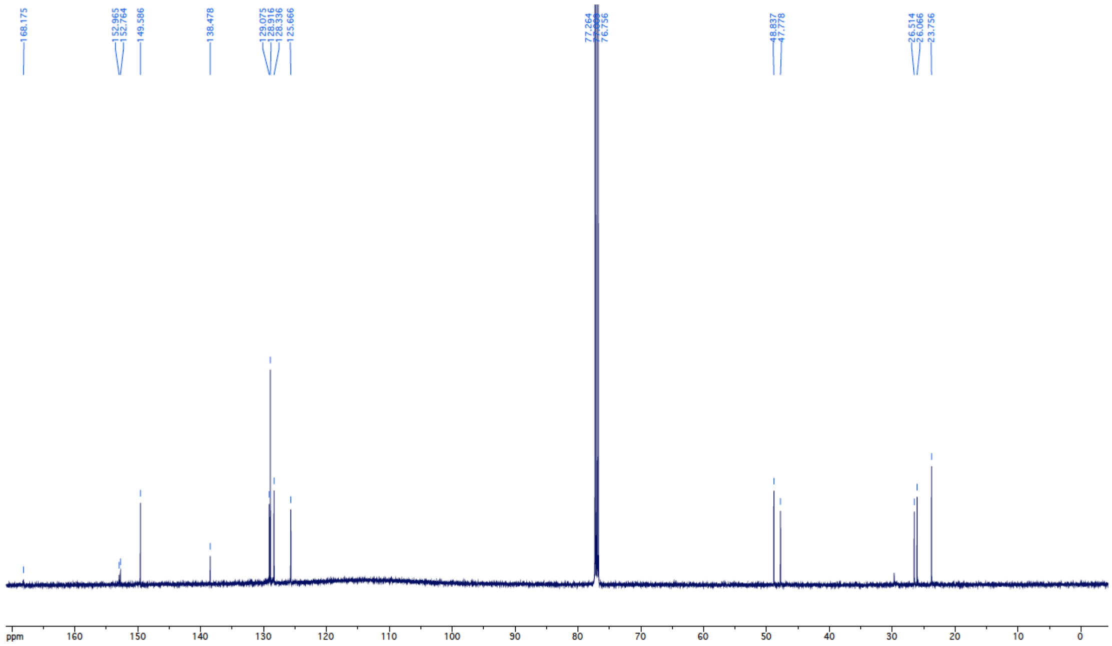
168.175
152.965
152.764
149.586
138.478
129.075
128.916
128.336
125.666
77.264
76.756
48.837
47.778
26.514
26.066
23.756
ppm
160
150
140
130
120
110
100
90
80
70
60
50
40
30
20
10
0

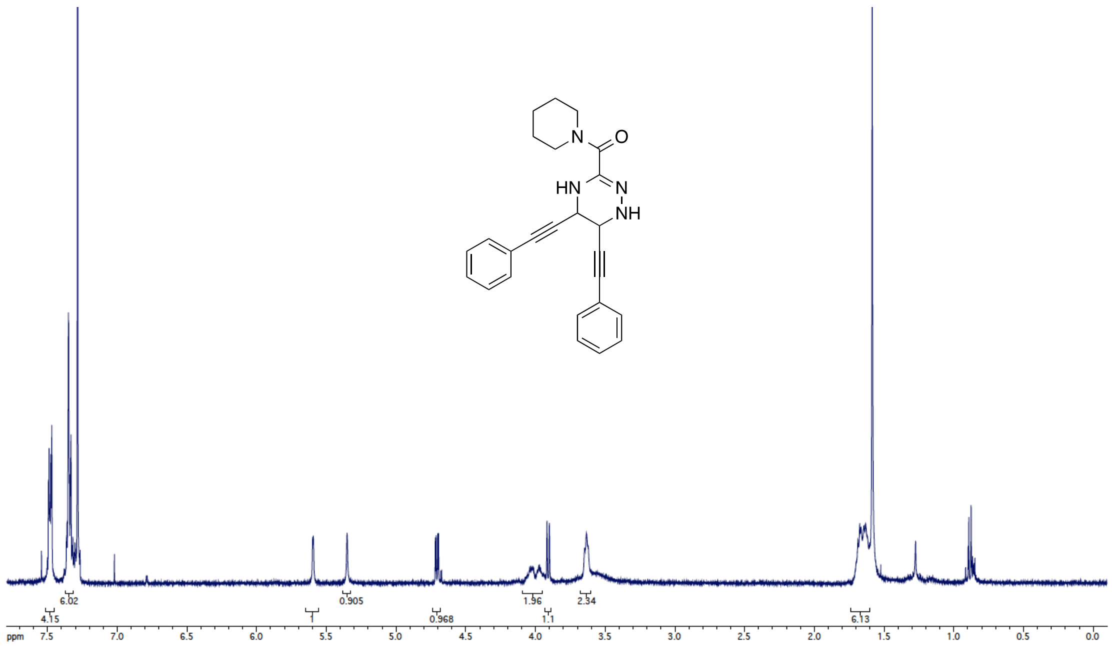
HN
N
NH
N
O
6.02
4.15
1
0.905
0.968
1.96
1.1
2.34
6.13
ppm
7.5
7.0
6.5
6.0
5.5
5.0
4.5
4.0
3.5
3.0
2.5
2.0
1.5
1.0
0.5
0.0

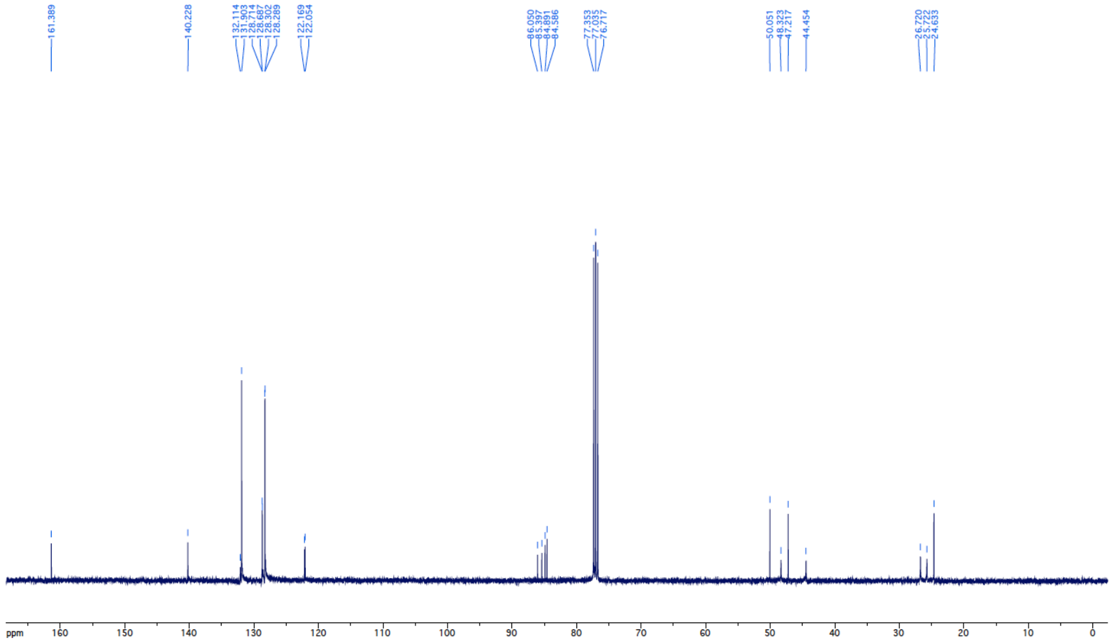
161.389
140.228
132.114
131.903
128.714
128.687
128.302
128.289
122.169
122.054
86.050
85.397
84.891
84.586
77.353
77.035
76.717
50.051
48.323
47.217
44.454
26.720
25.722
24.633
ppm
160
150
140
130
120
110
100
90
80
70
60
50
40
30
20
10
0

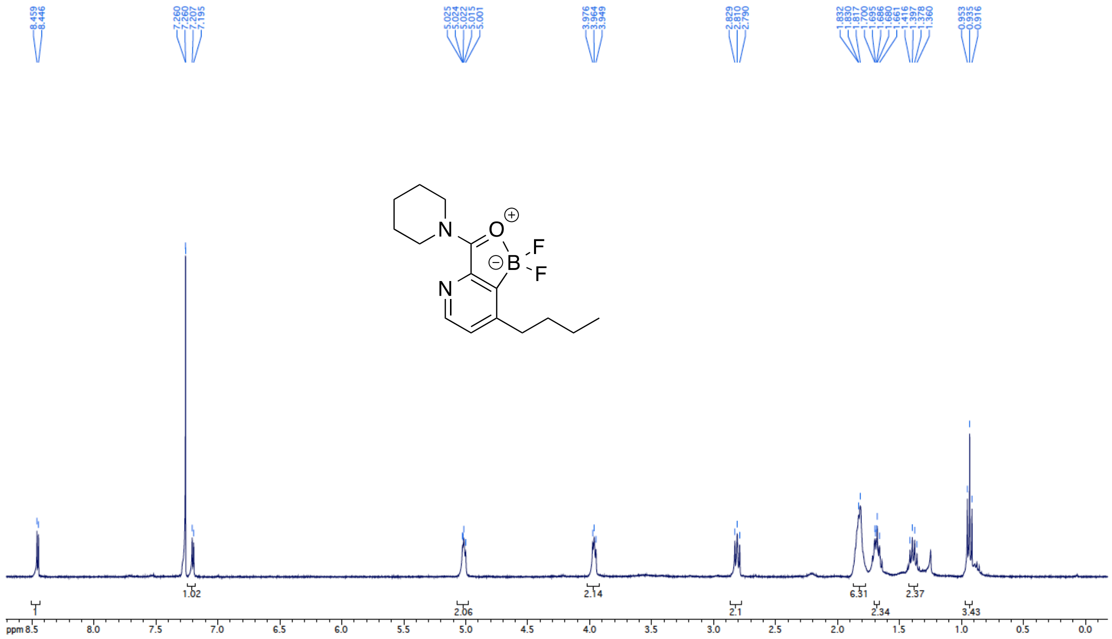
8.459
8.446
7.260
7.260
7.207
7.195
5.025
5.024
5.022
5.015
5.001
3.976
3.964
3.949
2.829
2.810
2.790
1.832
1.830
1.817
1.700
1.695
1.686
1.680
1.661
1.416
1.397
1.378
1.360
0.953
0.935
0.916
N
O
B
F
F
N
1
1.02
2.06
2.14
2.1
6.31
2.34
2.37
3.43
ppm 8.5 8.0 7.5 7.0 6.5 6.0 5.5 5.0 4.5 4.0 3.5 3.0 2.5 2.0 1.5 1.0 0.5 0.0

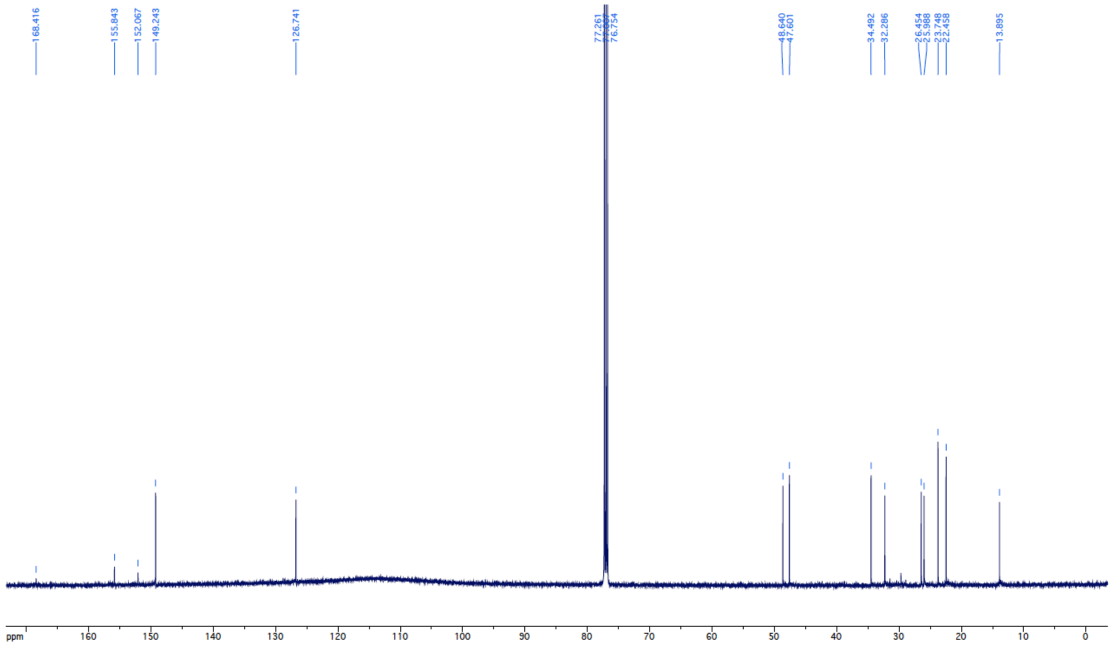
168.416
155.843
152.067
149.243
126.741
77.261
76.754
48.640
47.601
34.492
32.286
26.454
25.988
23.748
22.458
13.895
ppm 160 150 140 130 120 110 100 90 80 70 60 50 40 30 20 10 0

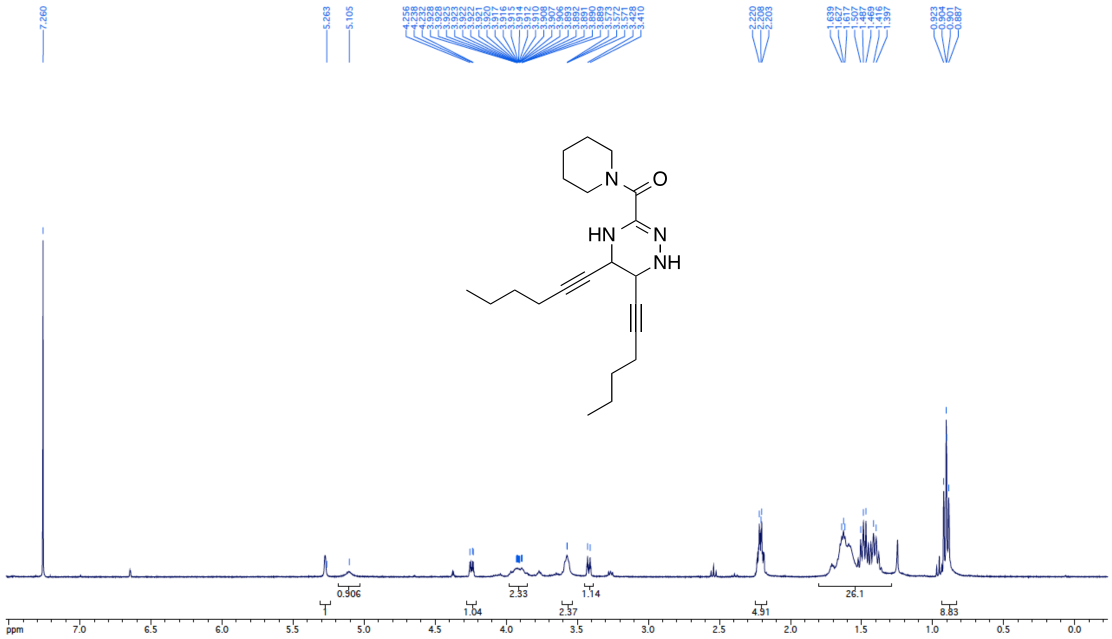

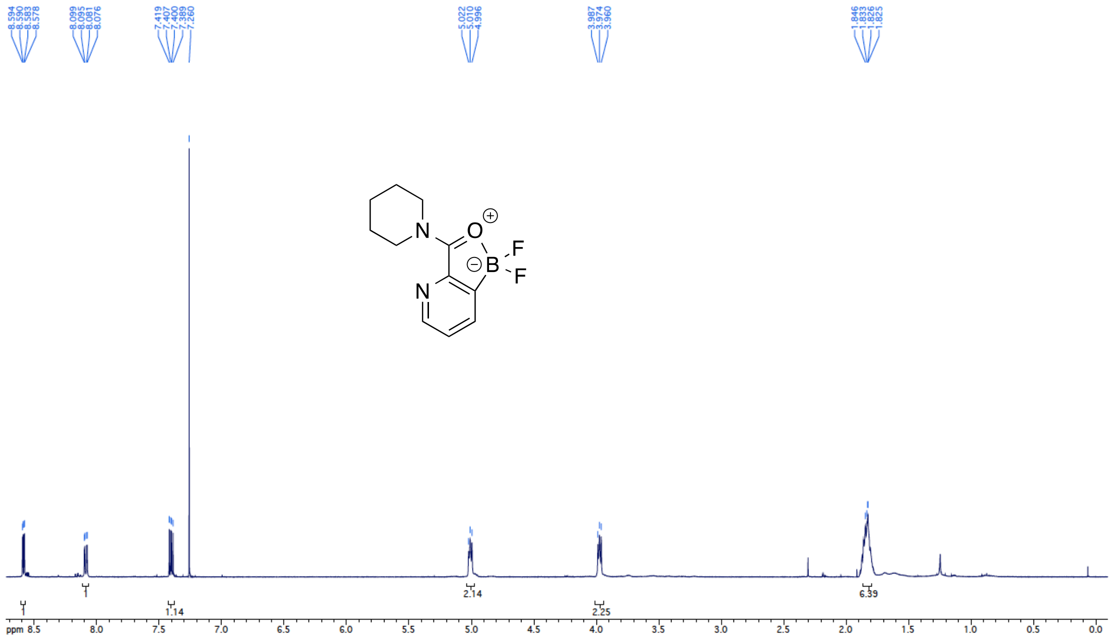
8.594
8.590
8.583
8.578
8.099
8.095
8.081
8.076
7.419
7.407
7.400
7.389
7.260
5.022
5.010
4.996
3.987
3.974
3.960
1.846
1.833
1.826
1.825
N
O
B
F
F
N
1
1
1.14
2.14
2.25
6.39
ppm

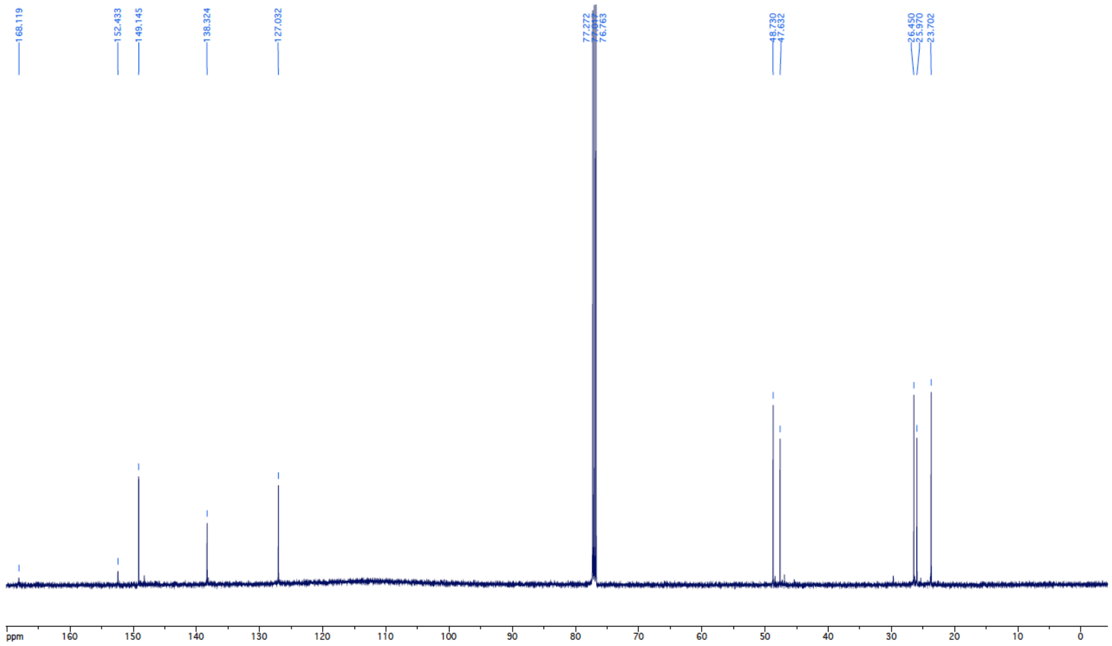
168.119
152.433
149.145
138.324
127.032
77.272
77.017
76.763
48.730
47.632
26.450
25.970
23.702
ppm

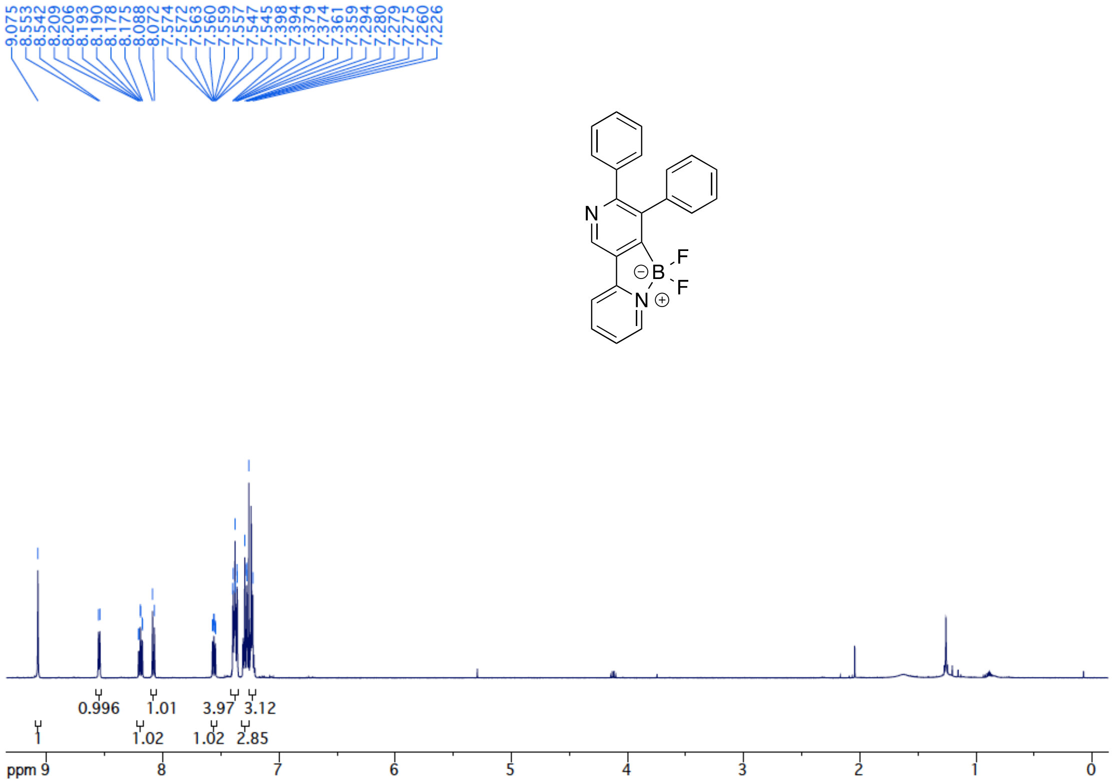
9.075
8.553
8.542
8.209
8.206
8.193
8.190
8.178
8.175
8.088
8.072
7.574
7.572
7.563
7.560
7.559
7.557
7.547
7.545
7.398
7.394
7.379
7.374
7.361
7.359
7.294
7.280
7.279
7.275
7.260
7.226
0.996
1.01
3.97
3.12
1
1.02
1.02
2.85
ppm
9
8
7
6
5
4
3
2
1
0
N
B
F
F
N

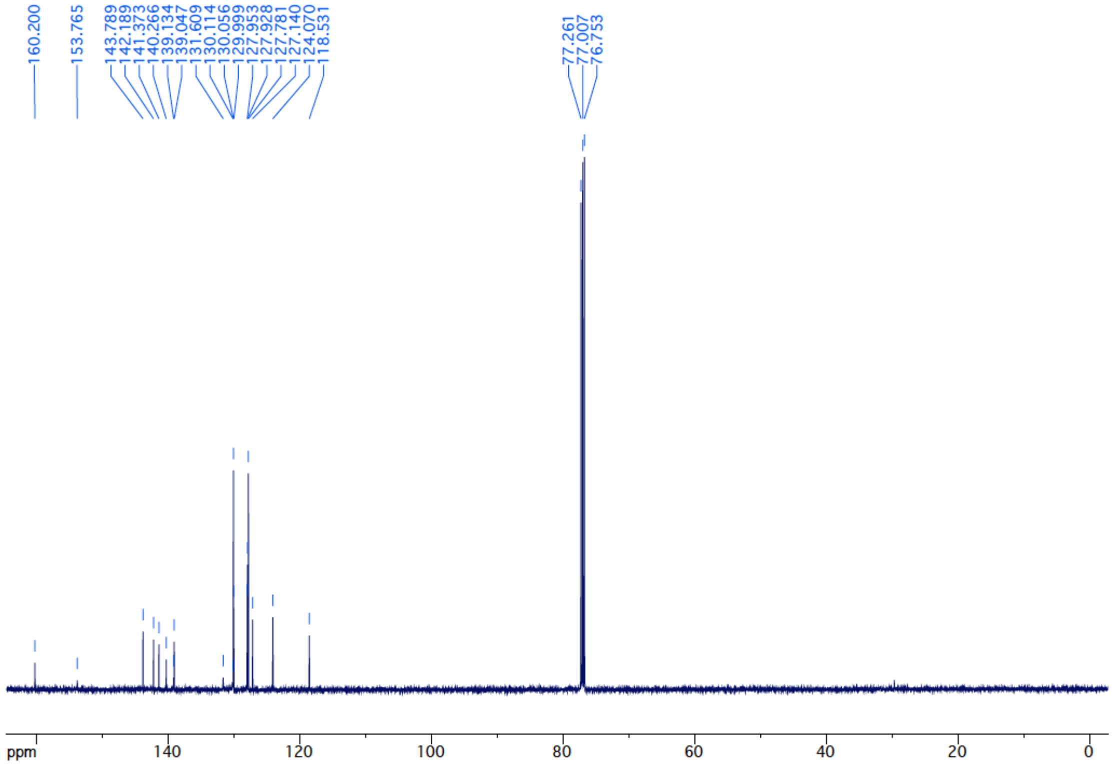
160.200
153.765
143.789
142.189
141.373
140.266
139.134
139.047
131.609
130.114
130.056
129.999
127.953
127.928
127.781
127.140
124.070
118.531
77.261
77.007
76.753
ppm
140
120
100
80
60
40
20
0

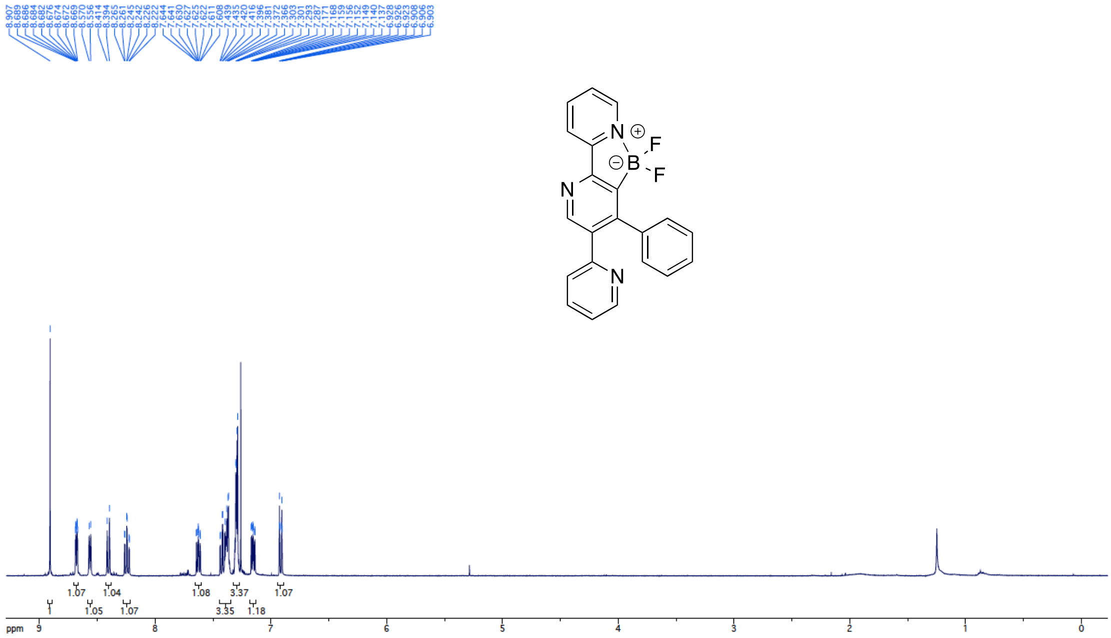

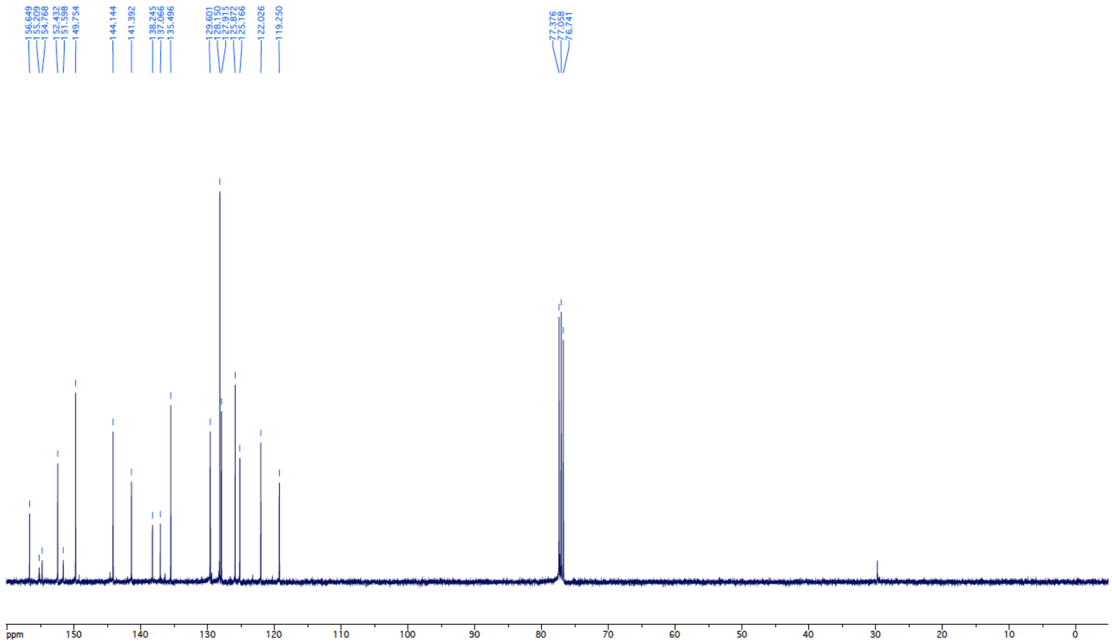

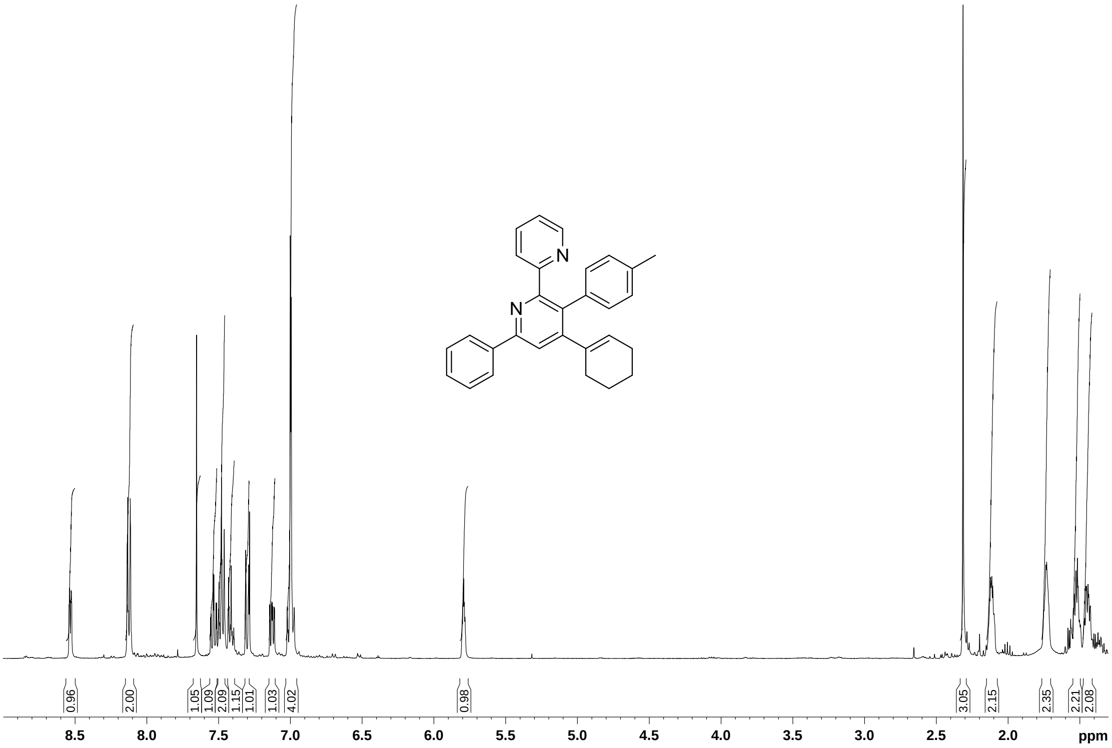
N
N
0.96
2.00
1.05
1.09
2.09
1.15
1.01
1.03
4.02
0.98
3.05
2.15
2.35
2.21
2.08
8.5 8.0 7.5 7.0 6.5 6.0 5.5 5.0 4.5 4.0 3.5 3.0 2.5 2.0 ppm

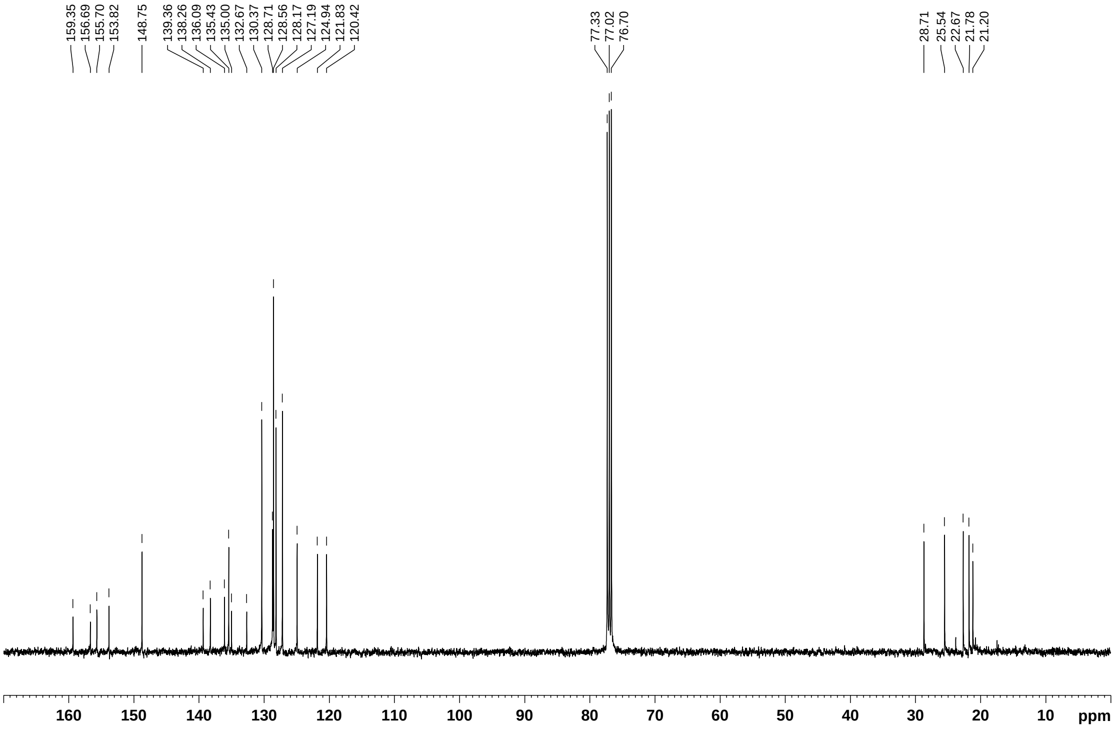
159.35
156.69
155.70
153.82
148.75
139.36
138.26
136.09
135.43
135.00
132.67
130.37
128.71
128.56
128.17
127.19
124.94
121.83
120.42
77.33
77.02
76.70
28.71
25.54
22.67
21.78
21.20
160 150 140 130 120 110 100 90 80 70 60 50 40 30 20 10 ppm

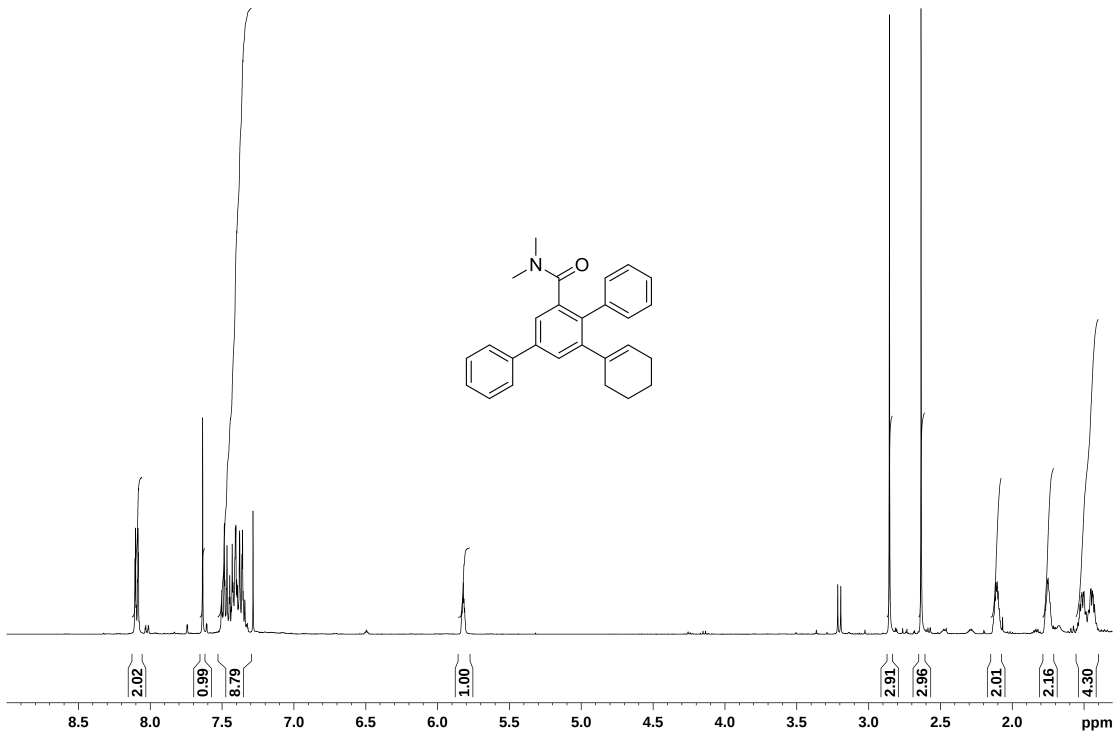
N
O
2.02
0.99
8.79
1.00
2.91
2.96
2.01
2.16
4.30
8.5
8.0
7.5
7.0
6.5
6.0
5.5
5.0
4.5
4.0
3.5
3.0
2.5
2.0
ppm

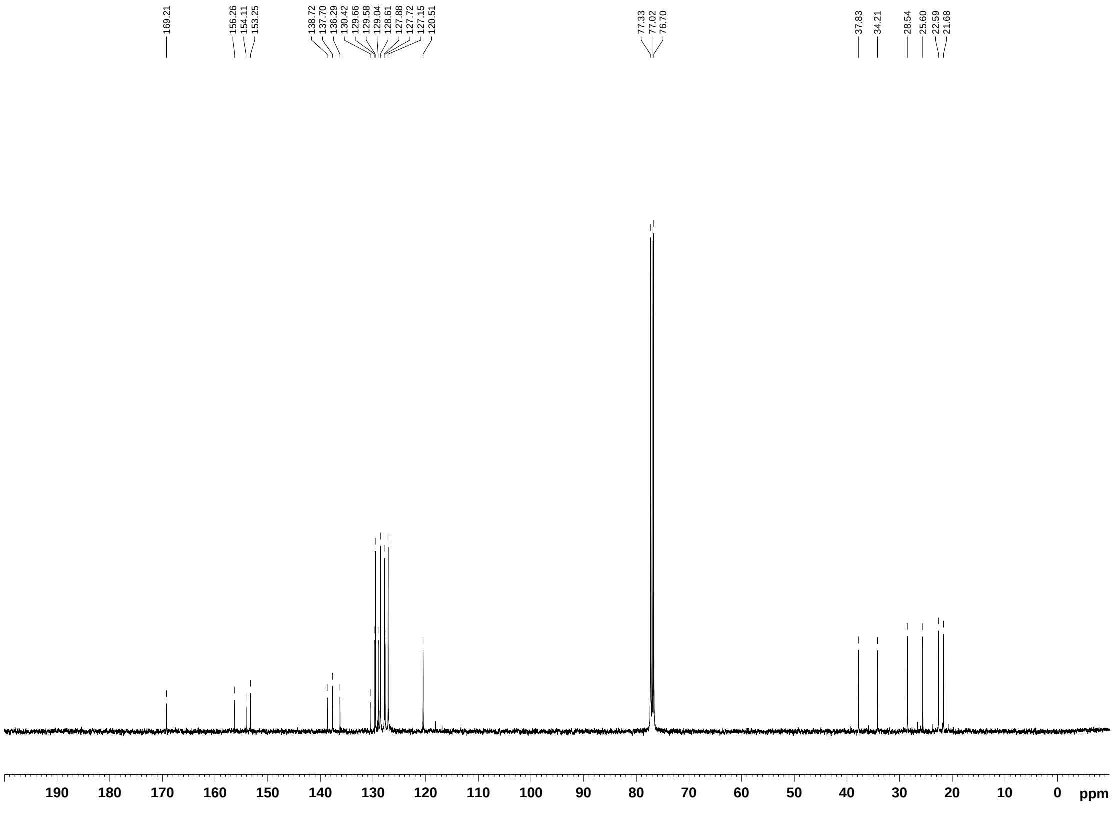
169.21
156.26
154.11
153.25
138.72
137.70
136.29
130.42
129.66
129.58
129.04
128.61
127.88
127.72
127.15
120.51
77.33
77.02
76.70
37.83
34.21
28.54
25.60
22.59
21.68
190
180
170
160
150
140
130
120
110
100
90
80
70
60
50
40
30
20
10
0
ppm

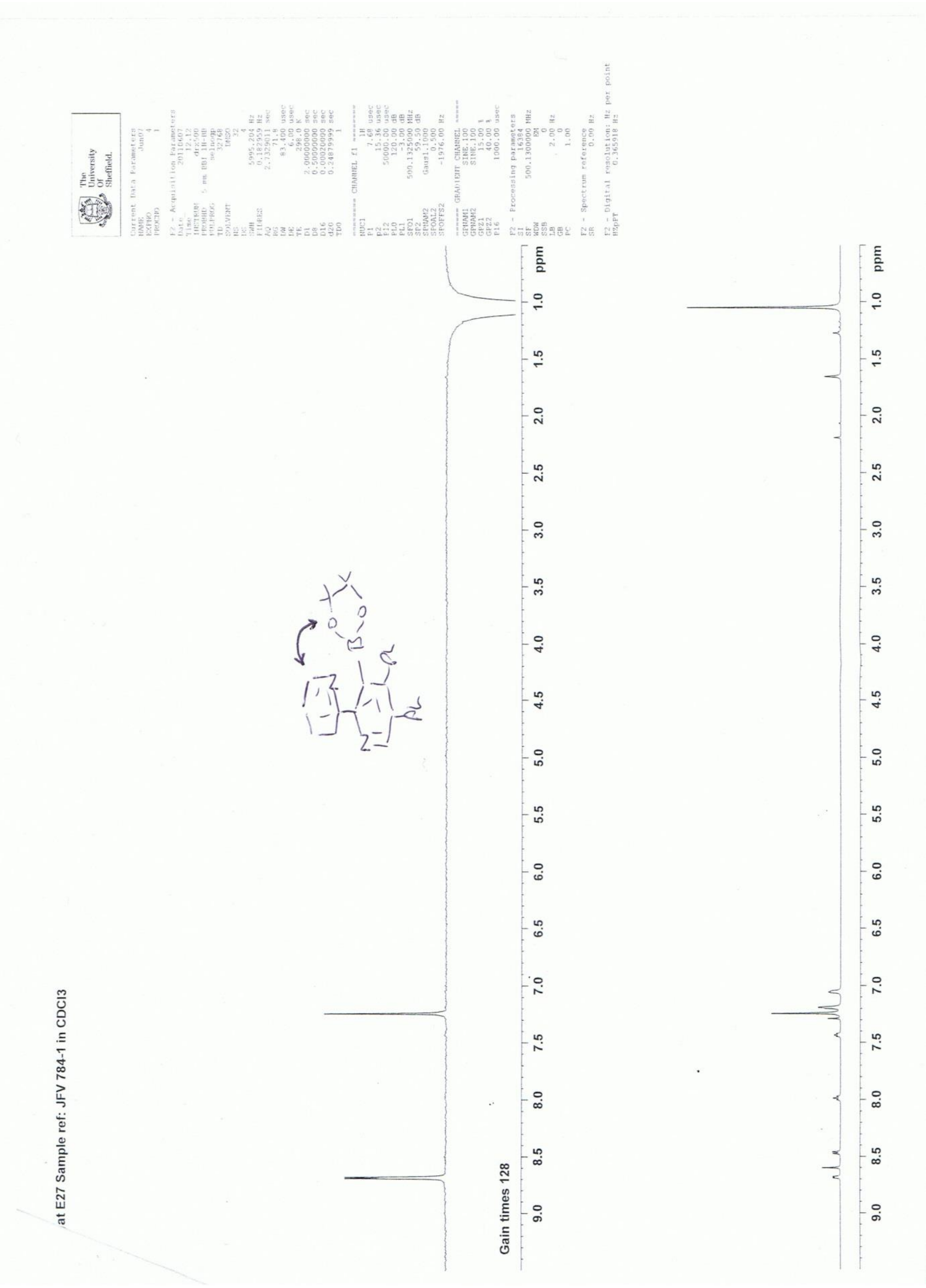
at E27 Sample ref: JFV 784-1 in CDCl3
Gain times 128
The University Of Sheffield.
ppm

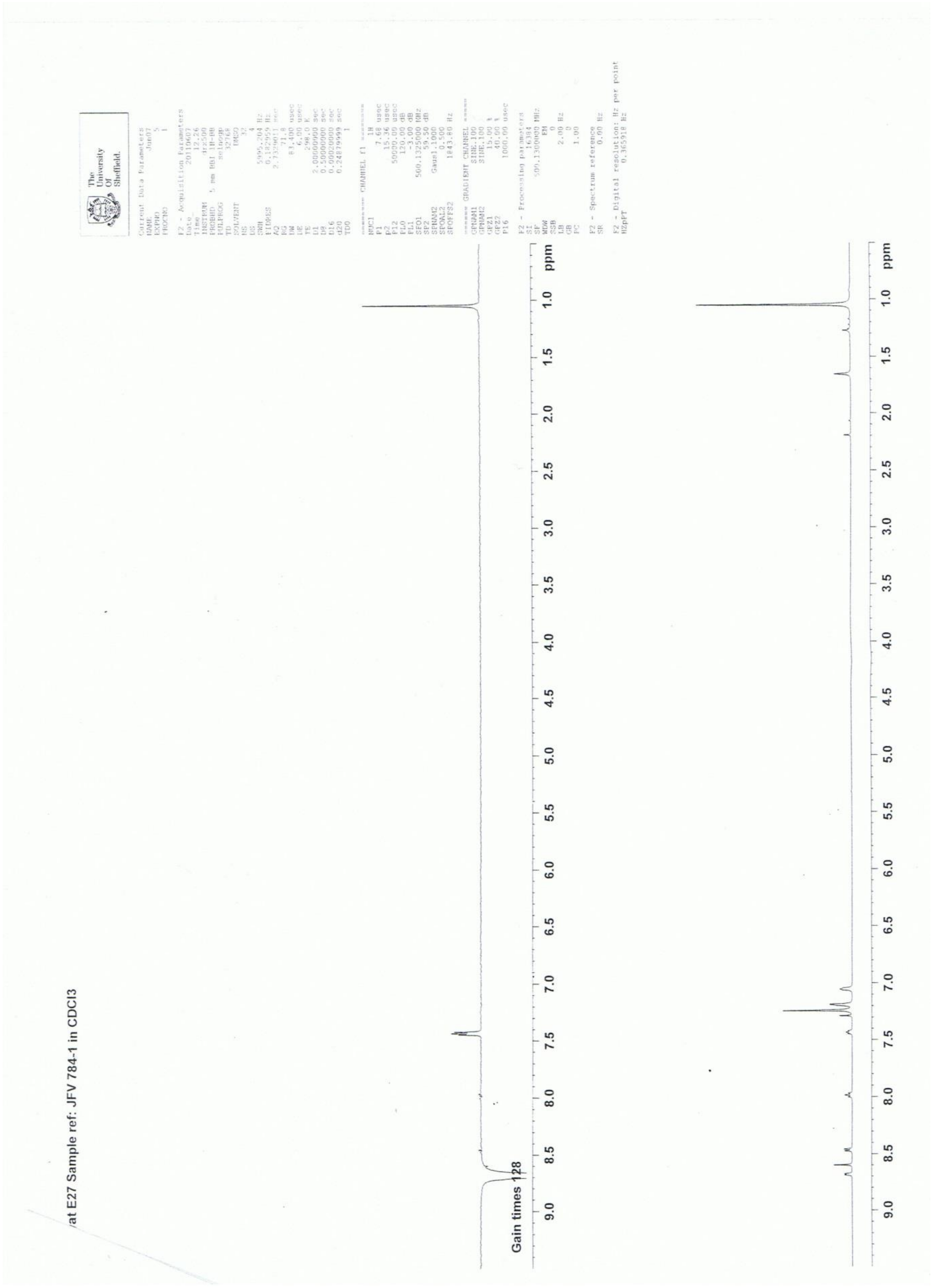
at E27 Sample ref: JFV 784-1 in CDCl3
Gain times 128
ppm
The University Of Sheffield.

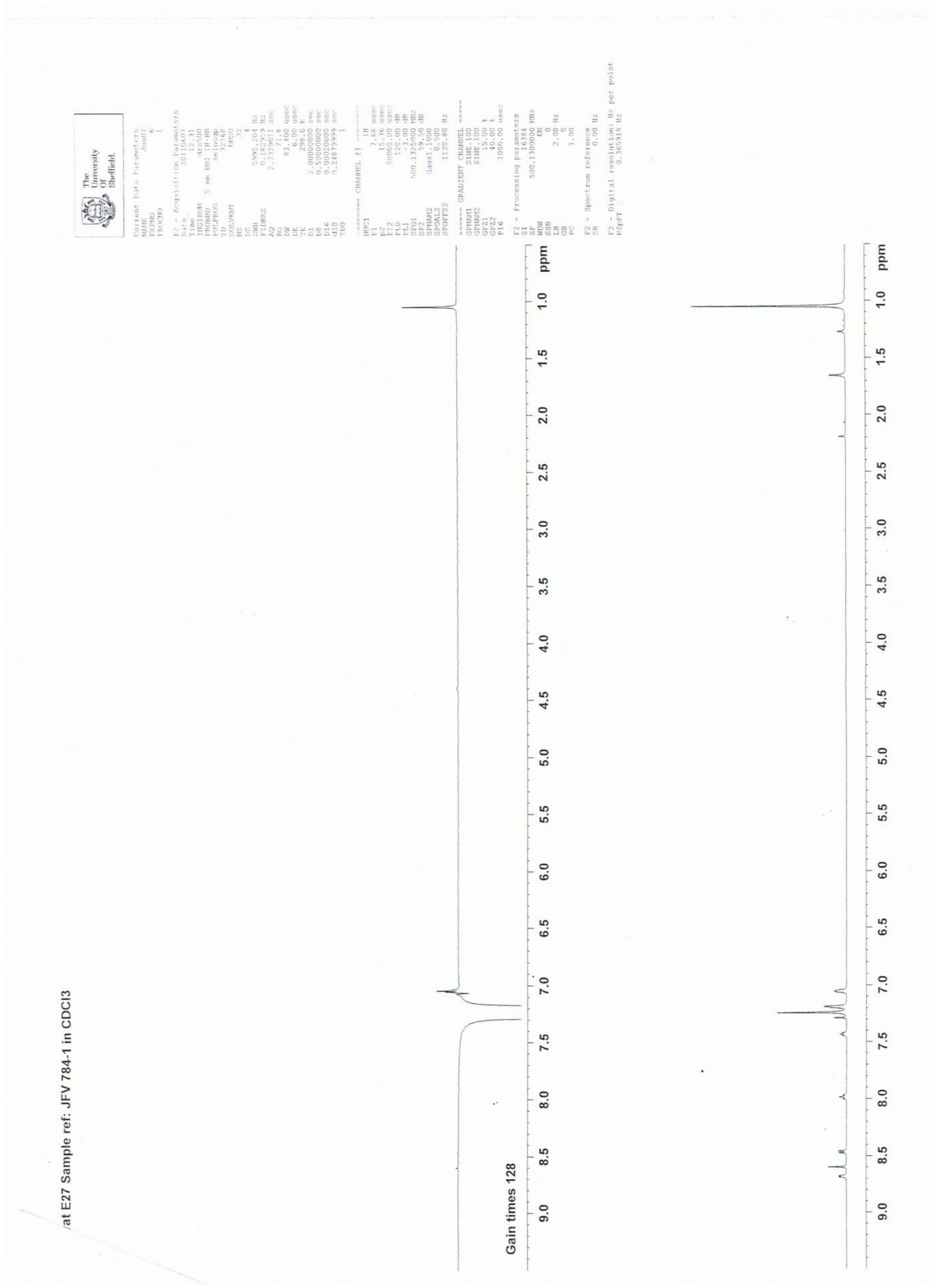
at E27 Sample ref: JFV 784-1 in CDCl3
Gain times 128
The University Of Sheffield
ppm
9.0 8.5 8.0 7.5 7.0 6.5 6.0 5.5 5.0 4.5 4.0 3.5 3.0 2.5 2.0 1.5 1.0

X Ray structures

The structures of compounds **4**, **17**, **3** and **28a** were confirmed by X-ray crystallography. CCDC 1005645-1005648 contain the supplementary crystallographic data. These data can be obtained free of charge from the Cambridge Crystallographic Data Centre via www.ccdc.cam.ac.uk/data_request/cif.
